# Surface-ligand-triggered synthetic control of defects in nanocrystals toward high-efficiency blue electroluminescence

**DOI:** 10.1016/j.xinn.2026.101273

**Published:** 2026-01-20

**Authors:** Qingli Cao, Qiuting Cai, Yifeng Feng, Xinyang Wang, Dingshuo Zhang, Yun Gao, Haoran Zhang, Meiyi Zhu, Yifan He, Haiping He, Zhizhen Ye, Xingliang Dai

**Affiliations:** 1 School of Materials Science and Engineering, State Key Laboratory of Silicon and Advanced Semiconductor Materials, Zhejiang University, Hangzhou 310027, China; 2 Wenzhou Key Laboratory of Novel Optoelectronic and Nano Materials, Engineering Research Centre of Zhejiang Province, Institute of Wenzhou, Zhejiang University, Wenzhou 325006, China; 3 Wenzhou XINXINTAIJING Tech. Co., Ltd., Wenzhou 325006, China

**Keywords:** perovskite nanocrystal, acid ligand, halogen activity regulation, defect passivation, blue electroluminescence

## Abstract

Colloidal nanocrystals have emerged as promising building blocks for optoelectronic applications. Although substantial research has focused on the roles of surface ligands in controlling the crystal structure, morphology, dispersibility, and surface properties of nanocrystals, limited attention has been devoted to ligand-triggered internal defect regulation. Here, we exemplify that the defects of blue-emitting CsPb(Br_x_Cl_1__–__x_)_3_ nanocrystals can be extensively regulated with surface ligands, enabling outstanding optical and electroluminescence performance. The ionization reaction of the strong dodecylbenzenesulfonic acid (DBSA) ligand, combined with its interactions with the precursors, modulates the concentration of halide ions in the reactant and controls activity. The increased DBSA results in a significantly reduced chlorine content in the nanocrystals and thus suppresses the formation of internal chlorine-related defects, evidenced by thermal admittance spectroscopy and optical characterization. Immediate application of this understanding allows defect-less CsPb(Br_x_Cl_1__–__x_)_3_ nanocrystals with efficient radiative recombination to be synthesized by controlling the DBSA dosage, realizing blue light-emitting diodes with an external quantum efficiency of 24.5% and a brightness of over 1,000 cd m^−2^ at 470 nm. This work provides new insights into the underlying mechanisms governing how ligands influence nanocrystal properties beyond those well-known functions, advancing nanocrystal synthesis from empirical exploration to rational design paradigms.

## Public summary

• Strong acid ligands modulate Cl defects, generalizing ligand-triggered synthetic control of holistic defects.
• Pure-blue perovskite LEDs emitting at 470 nm achieve a record external quantum efficiency of 24.5%.
• Mechanism studies of ligand-driven defect suppression advance the rational design of colloidal nanocrystals.

## Graphical abstract

**Figure:**
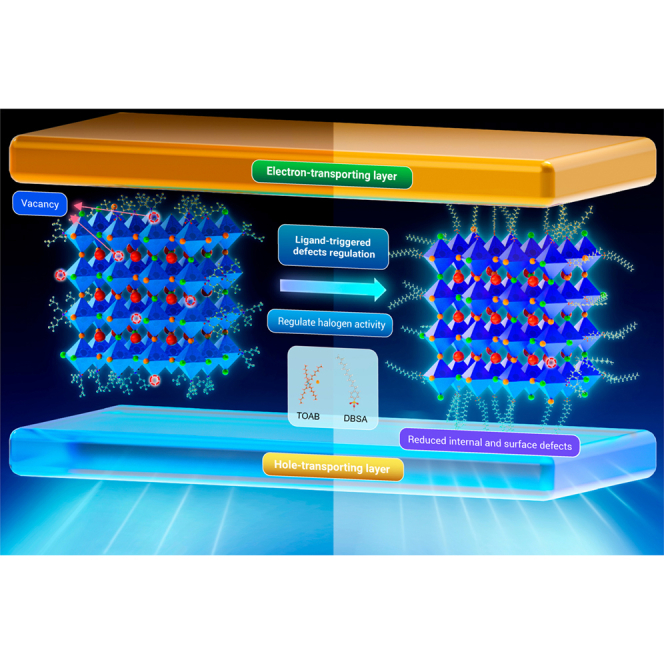

## Introduction

The intriguing features induced by nanosurface and size effects have facilitated the unprecedented rapid development of colloidal nanocrystals in next-generation optoelectronic devices, biomedicine, and quantum computation.^1^^,^^2^^,^^3^ During the past decade, perovskite nanocrystals emerged as a frontier in electroluminescence due to their narrow emissions and feasible color modulation.^4^^,^^5^^,^^6^ Efficient blue electroluminescence becomes the most critical challenge toward further advancement for applications such as full-color displays.^7^^,^^8^^,^^9^ Blue emission could be achieved through strong confinement from the reduced dimensions of the CsPbBr_3_ nanocrystal. However, the synthesis of ultra-small nanocrystals or ultra-thin nanoplates induces great challenges in ensuring size uniformity, accompanied by the delicate control of surface ligands, a trade-off for colloidal stability and carrier transport.^10^^,^^11^^,^^12^^,^^13^^,^^14^ Another approach to realizing blue emission involves introducing chlorine via halogen doping, which allows continuous spectrum adjustment across the green to the deep-blue region.^15^^,^^16^ In this regard, the composition can feasibly adjust the emission, providing great synthesis versatility for perovskite nanocrystals with targeted wavelengths. Nonetheless, mixed halogen perovskite nanocrystals suffer from low photoluminescence quantum yield (PLQY) induced by deep-level chlorine defects.^9^^,^^17^ Controlling the chlorine content and reducing chlorine-related internal defects is key to improving the luminescence efficiency of CsPb(Br_x_Cl_1__–__x_)_3_ nanocrystals.^18^

During the synthesis, two factors synergistically dominate the properties of colloidal nanocrystals, namely precursors (e.g., lead halide and cesium halide for perovskite nanocrystals) and ligands (e.g., oleylamine and oleic acid [OA]), which evolve into the inorganic core and the organic ligand shell, respectively.^19^^,^^20^ Specifically, the concentration and activity of precursors affect the composition and inherent properties of the inorganic core, while organic ligands modulate the crystal structure, morphology, dispersibility, and surface properties.^21^^,^^22^^,^^23^^,^^24^ For example, in mixed-halide blue-emission perovskite systems, precursor components and ion activity are key factors that affect the band gap of nanocrystals, usually altered by controlling the ratio of chloride to bromide in the precursor, while ligands are employed in morphology regulation and surface defect passivation.^25^^,^^26^^,^^27^ In this context, the influence of ligands on the inorganic core of nanocrystals is customarily overlooked; thus, the impact and controllability of ligands on components and defects remain an enigmatic issue and call for intense investigations.

Here, we systematically unraveled the acid-ligand-triggered component regulation and internal defect suppression in synthesizing mixed-halide perovskite nanocrystals. The nontrivial involvement of dodecylbenzenesulfonic acid (DBSA) in controlling the chlorine-bromine ratio of nanocrystals was authenticated, even though it does not contain inorganic perovskite components. This provides an opportunity to suppress internal halogen vacancies in the nanocrystals, whose defects are highly related to the halogen content, and regulate the optical emission by varying the dosage of DBSA. Following this perspective, CsPb(Br_x_Cl_1__–__x_)_3_ nanocrystals with different amounts of DBSA were synthesized, and their optical properties and electroluminescence performance were investigated. Using this typical acid ligand, we elucidate the chemical mechanisms by which ligand design modulates halogen activity and suppresses intrinsic defects in nanocrystals, highlighting the superiority of ligand regulation in controlling the surface and internal defects of nanocrystals.

## Materials and methods

### Materials

Cesium carbonate (Cs_2_CO_3_, 99.9%), PbBr_2_ (99.999%), PbCl_2_ (99.999%), and DBSA (≥95%) were purchased from Sigma-Aldrich. Rubidium carbonate (Rb_2_CO_3_, 99.9%), formamidine acetate (FA(Ac), 99%), bis(2,4,4-trimethylpentyl)phosphinic acid (PA; 90%), didodecyldimethylammonium chloride (DDAC; 98%), didodecyldimethylammonium bromide (DDAB; 98%), tetra-*n*-octylammonium bromide (TOAB; 98%), ethyltributyl phosphonium bromide (ETBPB; ≥99%), ethyl acetate (EA; 99%), nickel acetate tetrahydrate (NiAc_2_·4H_2_O, 99.9%), ethanolamine (≥99.0%), Nafion perfluorinated resin (PFI) solution (5 wt % in a mixture of lower aliphatic alcohols and water, contains 45% water), and LiF (99.99%) were purchased from Shanghai Macklin Biochemical. 1,3,5-tris(1-phenyl-1H-benzimidazol-2-yl) benzene (TPBi), PEDOT:PSS, and PO-T2T were purchased from Xi'an Yuri Solar Co. Ltd. Oleic acid (OA, 90%, Alfa Aesar), n-Octanoic acid (OTAc, 99%, Aladdin), ultradry toluene (≥99.5%, Chengdu Chron Chemical), ultradry octane (97%, J&K Scientific), and PF8Cz (Dongguan Volt-Amp Optoelectronics Technology) were purchased from the respective suppliers and all chemicals were used directly without further purification.

### Synthesis of nanocrystals

PbX_2_ stock solution was prepared by dissolving PbBr_2_ (4 mmol), PbCl_2_ (1 mmol), and TOAB (7.25 mmol) in toluene (50 mL) at room temperature and filtered through a 0.22 μm filter after complete dissolution. The Cs/FA/Rb-PA solution was prepared by mixing Cs_2_CO_3_ (0.85 mmol), Rb_2_CO_3_ (0.085 mmol), and FAAc (0.15 mmol) with PA (10 mL) at 80°C. Nanocrystals were synthesized in a nitrogen glove box, taking 5.3 mL PbX_2_ precursor in a 20 mL vial, and x mL (x = 0, 0.2, 0.4, 0.6, 0.8, or 1.0) DBSA (1 g mL^−1^ in toluene) was added under vigorous stirring. After 1 min of stirring, Cs/FA/Rb-PA precursor (0.5 mL) was injected swiftly. DDAC solution (1 mL, 0.1 g mL^−1^ in toluene) was added after reacting for 2 min and kept under stirring for another 5 min.

The nanocrystals with emission at 470 nm, synthesized with or without DBSA, were obtained by adjusting the amount of DDA^+^. To achieve a similar surface passivation effect, the nanocrystals with emission at 470 nm synthesized without DBSA were treated with a mixture of DDAB and DDAC (see Table S2 for details). To create the pure bromine nanocrystals, all chlorides were replaced by bromides: PbCl_2_ was substituted by PbBr_2_ and DDAB (10 mg mL^−1^) was used in place of DDAC.

The purification process was carried out under air conditions. EA was added to the crude solution at a volume ratio of 2:1 (this value gradually changes from 2:1 to 4:1 as DBSA increases, due to the reduced size of nanocrystals needing more anti-solvents for precipitation), and then centrifuged at 8,000 rpm for 3 min. The precipitate was collected and dispersed in octane. The purification process was repeated two times for device fabrication and characterization. To ensure precise controllability over the emission wavelength, the temperature was maintained at ∼25°C throughout the synthesis process.

### LED fabrication

The indium tin oxide (ITO) substrate was sequentially ultrasonicated in acetone (20 min), deionized water (15 min), and ethanol (15 min), followed by treatment with oxygen plasma for 15 min. Under ambient conditions (∼50% relative humidity [RH]), 50 μL NiO_x_ precursor (0.06 M NiAc_2_·4H_2_O and 0.06 M ethanolamine dissolved in ethanol) was spin coated on the substrate at 5,000 rpm for 45 s and annealed at 280°C for 30 min. PEDOT:PSS solution premixed with PFI (1:1 volume ratio) was then spun onto the NiO_x_ layer at 10,000 rpm  for 15 s, followed by annealing at 150°C for 15 min. The substrates were transferred into a glove box with N_2_ conditions for further fabrication. PF8Cz (8 mg mL^−1^ in chlorobenzene) was spin coated at 3,000 rpm for 35 s and annealed at 150°C for 30 min. The nanocrystal solution was diluted with n-octane, the additive ETBPB (7.5 mM in toluene) was added (10:1 volume ratio) to further passivate surface defects, and the mixture was filtered after being mixed evenly. Subsequently, the blue perovskite nanocrystals were spin coated at 2,000 rpm for 50 s. Later, under a vacuum below 4 × 10^−4^ Pa, TPBi (5 nm, 0.3 Å s^−1^), PO-T2T (45 nm, 0.3 Å s^−1^), and LiF/Al electrodes (1/80 nm, 0.1/1.0 Å s^−1^) were deposited sequentially in a thermal evaporator.

### Optical characterizations

The UV-visible (UV-vis) absorption spectra of the nanocrystals were measured using an Agilent Cary 5000. The PL spectra and time-resolved fluorescence spectra were performed using OmniFluo990 with an excitation source of a xenon flash lamp and a 405-nm pulsed laser diode (EPL405, 58.6-ps pulse width), respectively. The absolute PLQYs were measured using a xenon lamp that was filtered to 405 nm as excitation wavelength and a home-designed integrating sphere coupled to a QE Pro spectrometer.^28^^,^^29^ The temperature-dependent PL measurements were conducted with a Zolix Instruments OmniFluo900 spectrometer equipped with a picosecond pulsed laser diode (405 nm, 58.6 ps) and a liquid nitrogen cooling instrument.

Transient absorption (TA) spectra were measured utilizing a femtosecond pump-probe spectroscopy setup. The output light from a light Conversion Pharos Yb:KGW laser (1,030 nm, 200 fs, 200 μJ per pulse, and a 100 kHz repetition rate) was split into two beams: one passed through an OptoSigma OSMS26-300 optical delay line and then focused onto a barium borate oxide (BBO) crystal to generate a probe beam and the other was introduced to a Light Conversion Orpheus-HP optical parameter amplifier to generate a pump beam at a desired wavelength of 405 nm, passed through a round continuously variable metallic neutral density filter and a chopper working at 40 kHz. During the measurements, the perovskite nanocrystal solution was irradiated by the pump beam. The optical power density of the pump light was attenuated to 3.36 μJ cm^−2^.

The picosecond time-resolved photoluminescence (TRPL) dynamics were obtained through the low-frequency mode of a streak camera (ST-10, Zolix). A femtosecond laser was used as the excitation source with a repetition rate of 1 kHz and an excitation wavelength of 405 nm. The excitation light intensity was 0.35 μJ cm^−2^ focused on the sample. The perovskite nanocrystal solutions were filled into quartz cuvettes with a 1-mm light path, and the sample solutions were under continuous stirring during TA and TRPL measurements.

### Defect-state characterizations

The space-charge-limited current (SCLC) measurement was based on the hole-only device with a multilayer structure of ITO/NiO_x_/PEDOT:PSS:PFI/PF8Cz/nanocrystals/MoO_3_/Al. The J-V curves of devices were collected in a forward scan (0–3 V, step: 0.01 V) using a Keithley 2400 in the dark. For the thermal admittance spectroscopy (TAS) measurement, a Zurich Instruments MFIA5M was utilized. The capacitance (*C*) was measured by applying an AC voltage with an amplitude of *V*_ac_ = 100 mV and varying the angular frequency (ω) from 10 Hz to 5 MHz. The *E*_a_ of the defects and the energetic profile of the trap density of states (tDOS) were calculated using the following equations^30^:

(Equation 1) $$\begin{array}{c} \omega_{T}{=2\pi fe}^{-\frac{E_{a}}{kT}} \end{array}\text{,}$$

(Equation 2) $$\begin{array}{c} E_{\omega }=kTln\frac{2\pi f}{\omega }\; \end{array},\text{and}$$

(Equation 3) $$\begin{array}{c} N_{T}\left(E_{\omega }\right)=-\frac{V_{bi}}{de}\frac{dC}{d\omega }\frac{\omega }{kT}\; \end{array}\text{,}$$

where *f* is the attempt-escape frequency, *k* is the Boltzmann constant, *V*_bi_ is the built-in potential, and *d* is the width of the depletion layer.

### Composition and crystal structure characterizations

Transmission electron microscopy (TEM) observations were conducted using a Hitachi HT-7700 microscope operated at 80 kV. Spherical aberration-corrected high-angle annular dark-field STEM (HAADF-STEM) observations were conducted using an FEI Titan G2 80-200 ChemiSTEM spherical aberration-corrected TEM operated at 200 kV. X-ray photoelectron spectroscopy (XPS) spectra were obtained on a ThermoFisher ESCALAB Xi+ equipment in an ultrahigh vacuum chamber with a vacuum of 8 × 10^−10^ Torr and Al Kα source. Crystal structures were analyzed by X-ray diffraction (XRD; Rigaku D/MAX 2500) operated at 40 keV and 40 mA with Cu Kα radiation (λ = 1.5406 Å). The actual contents of halogens were detected using ion chromatography (AQUION, DIONEX). The perovskite nanocrystal solution was dried with nitrogen, and the obtained solid was dissolved 1,000 times in deionized water for ion chromatography measurements.

### Device characterizations

The current density-luminance-voltage curves, external quantum efficiencies (EQEs), EL spectra, and Commission Internationale de l'Éclairage (CIE) coordinate characterizations of light-emitting diodes (LEDs) were measured with a Keithley 2400 electrometer and an integration sphere coupled with a QE Pro spectrometer.^31^ The EQE (*η*_EQE_) of the device is calculated using the following formula:

$$\eta_{\text{EQE}}=\frac{N_{p}}{N_{e}}=\frac{\int \frac{\Phi_{R}\left(\lambda \right)\cdot \lambda }{h\cdot c}d\lambda }{\frac{J\cdot A}{e}}\text{.}$$

The luminance (*L*) of the device is calculated using the following formula:

$$L=\frac{\Phi_{L}}{\pi \cdot A}=\frac{683\int_{380}^{780}\Phi_{R}\left(\lambda \right)\cdot V\left(\lambda \right)d\lambda }{\pi \cdot A}\text{,}$$

where *N*_p_ is the number of photons emitted by the LED, *N*_e_ is the number of injected electrons, *Φ*_R_(*λ*) is the radiant flux at wavelength *λ*, *h* is Planck’s constant, *c* is the speed of light in vacuum, *J* is the current density, *A* is the effective area of the device, *e* is the elementary charge, *Φ*_L_ is the total luminous flux, and *V*(*λ*) is the photopic vision function at wavelength *λ*.

The devices were swept from zero bias to forward bias, and a segmented voltage application method was adopted to accurately measure the efficiency at low brightness. The swept speed was 0.03/0.1/0.2 V per 100 ms with an additional integration time of spectral acquisition of 50 ms. The operational stability was conducted by applying a constant current density to the devices, and the EL spectra were recorded. The performance of the devices was measured in the glove box filled with nitrogen at room temperature.

## Results and discussion

### Activity modulation of halogens with strong organic acid ligands

**Figure 1:**
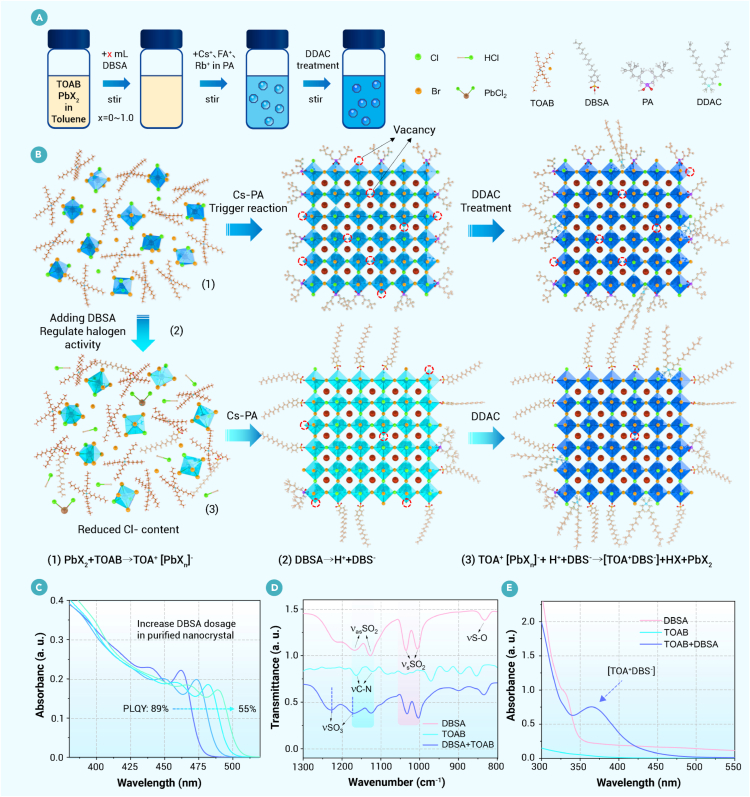
Activity modulation of halogens with strong organic acid ligands in the synthesis of nanocrystals (A) Schematic representation of nanocrystal synthesis and post-treatment processes. (B) Schematic illustration of the nanocrystal synthesis procedures and DBSA-mediated activity regulation of halide ions (some hydrogen atoms are omitted for the sake of simplicity), showing the formation of HCl and the precipitation of PbCl_2_ after adding DBSA to the precursor solution. (C) Absorption spectra transformation of the purified CsPb(Br_x_Cl_1__–__x_)_3_ nanocrystals (470 nm, with DBSA) after adding an increasing amount of DBSA (DBSA added to nanocrystal octane solution). (D) Fourier transform infrared spectra of DBSA, TOAB, and their equimolar mixtures. (E) Absorption spectra of DBSA, TOAB, and their equal mixtures dissolved in toluene.

Mixed-halide CsPb(Br_x_Cl_1__–__x_)_3_ nanocrystals were synthesized using a modified ligand-assisted reprecipitation method (see materials and methods for details),^32^^,^^33^ as shown in Figure 1A. The nucleation process of the nanocrystals is illustrated in Figure 1B, where the existing form of lead halides is represented as octahedra for simplicity. In this methodology, TOAB forms a complex with polar lead halide to enhance the solubility of halide in low-polarity solvent toluene (equation 1 in Figure 1B). The optimized PA was utilized for dissolving the cesium source, and the injection of cesium phosphate triggered the crystallization of CsPb(Br_x_Cl_1__–__x_)_3_ nanocrystals. DDAC post-treatment effectively passivates surface defects and modulates the emission wavelength of nanocrystals (Figure S1A).^34^ Notably, while the DDAC ligand does not inherently introduce Cl defects, an excess of DDAC increases the overall chloride content in the system, thereby making the generation of Cl defects more probable. A preliminary investigation revealed that factors such as the acid ligand species led to significant differences in the emission wavelength of nanocrystals, even under identical feed halogen ratios (Figure S1B). This prompted us to investigate the impact of acid ligands on halogen activity in the system to unveil the underlying mechanisms.

It is well established that the synthesis of perovskite nanocrystals is typically conducted in low-polarity organic solvents, such as 1-octadecene, octane, or toluene, to minimize structural damage. In this reactant, incomplete ionization of HX (X = Br or Cl) makes it not behave as a strong acid, as evidenced by its significantly lower acid dissociation constant (larger pKa) in organic solvents (e.g., toluene) than in aqueous solutions.^35^^,^^36^ The introduction of strong organic acids in the reactant generates numerous hydrogen ions, which could combine with halide ions to form low-reactive hydrogen halides, resulting in undesirable halogen content within the nanocrystals. In this sense, we may regulate nanocrystal emission by adjusting the halogen activity through organic acids.

DBSA has been employed in perovskite nanocrystal synthesis, particularly in room temperature systems, due to its superior surface coordination capability that outperforms traditional OA.^37^^,^^38^^,^^39^ Additional reports have highlighted that DBSA effectively suppresses phase segregation in mixed-halide nanocrystals.^40^ Furthermore, post-synthetic ion-pair engineering based on benzenesulfonate derivatives has been shown to regulate halide distribution and stability within these systems.^36^ Those studies predominantly focus on surface state modification or macroscopic crystallization effects and lack systematic theoretical interpretation of the intrinsic driving forces behind compositional and crystallographic changes. The DBSA ligand possesses strong acidity and undergoes partial ionization into H^+^ and DBS^−^ (equation 2 in Figure 1B) in the solvents for synthesizing nanocrystals, which are capable of adjusting halogen activity. After adding DBSA to the reactant, the pH value of the solution decreases from 6.0 to 1.0 (Figure S2), authenticating the ionization process of DBSA in toluene. The combination of the generated hydrogen ions with halogens could reverse the ionization reaction, forming the less reactive HX. Due to the large electronegativity of chlorine (3.16) compared with bromine (2.96), chloride ions are inclined to deplete from the organic solution to form nonionized HCl.^41^ This would substantially decrease the chlorine-to-bromine ratio in the nanocrystals and change the halogen activity within the reactant. To validate the hypothesis that the chloride ions are preferred to combine with the hydrogen ions, we introduced DBSA in the purified CsPb(Br_x_Cl_1__–__x_)_3_ nanocrystal reactant. The absorption spectra are progressively redshifted, and the absorbance is gradually reduced upon increasing the amount of DBSA (Figure 1C), inferring the reduced chlorine component in the nanocrystals. This process generates massive chlorine vacancies, resulting in a reduction in PLQY. Introducing another strong organic trifluoromethanesulfonic acid (TFMSA) also shows analogous behavior in the CsPb(Br_x_Cl_1__–__x_)_3_ nanocrystal reactant, while for pure CsPbBr_3_ nanocrystals, non-distinguishable spectral shifts are observed (Figure S3). This indicates that strong organic acids exert similar effects on halogen activity in mixed-halide systems; they can modulate halogen activity via their acidity, which in turn regulates nanocrystal composition. However, since TFMSA is not a competent ligand, the corresponding nanocrystals exhibit inferior optical properties, with the PLQY of those emitting at 470 nm only reaching 48%. Collectively, these findings highlight that strong organic acids modulate the composition of nanocrystals beyond their conventional functionality.

Besides the ionization reaction of the DBSA, the interactions between DBSA and other precursors were investigated through Fourier transform infrared (FTIR) spectroscopy and absorption spectra. As shown in Figure 1D, FTIR spectroscopy reveals the stretching vibration peak (833 cm^−1^) of the S–O bond, the symmetric stretching vibration peaks (1,005 and 1,035 cm^−1^), and antisymmetric stretching vibration peaks (1,127 and 1,166 cm^−1^) of the S=O bond in DBSA,^37^ as well as the symmetric stretching vibration peaks (1,121 and 1,163 cm^−1^) of the C–N bond in TOAB. After mixing DBSA and TOAB, the stretching vibrations of the S=O and S–O bonds weakened. In contrast, the symmetric stretching vibration (1,228 cm^−1^) and antisymmetric stretching vibration (1,170 cm^−1^) of SO_3_ intensified, suggesting that the combination of DBSA and TOAB generates sulfonic acid groups. Furthermore, the absorption spectrum displays a new strong absorption peak following the mixing of DBSA with TOAB (Figure 1E). These results suggest that DBSA reacts with the quaternary ammonium salt to form a complex, which disturbs the equilibrium between TOA^+^ and PbX_n_^−^ and results in the precipitation of lead halides. As a verification, we introduced an adequate amount of DBSA into the precursor solution (PbBr_2_, PbCl_2_, and TOAB in toluene), causing the system to become turbid and precipitate. Ion chromatography characterizations were performed on the original precursor solution, supernatant, and precipitate, revealing the highest chlorine content in the precipitate (Figure S4). This verifies that the introduction of DBSA mainly precipitates PbCl_2_, originating from its pronounced molecular polarity.^36^ The precipitate could be redissolved in toluene upon the addition of TOAB. The conclusion underscores that the strongly organic acid ligand additives can regulate the ion concentration of bromide and chloride in the reactant during the synthesis of nanocrystals, enabling the activity modulation of halogens (equation 3 in Figure 1B).

### Size and composition regulation of nanocrystals

**Figure 2:**
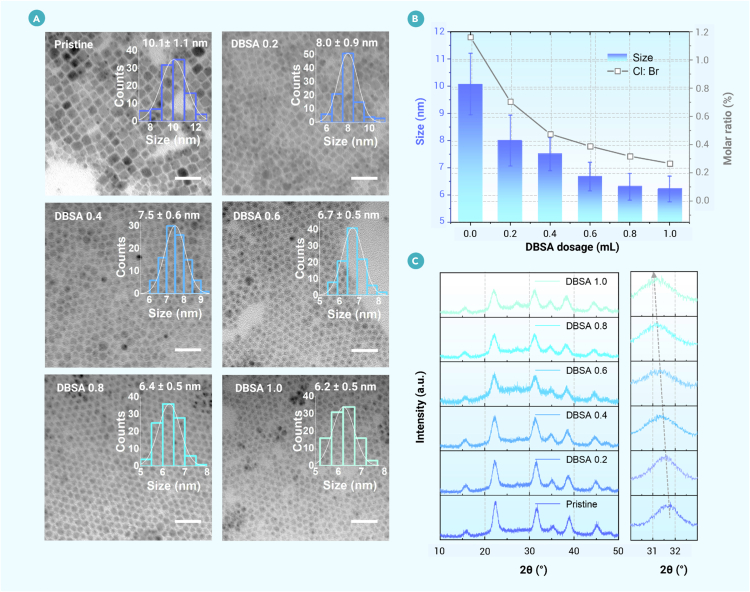
Size and compositional regulation of the CsPb(Br_x_Cl_1__–__x_)_3_ nanocrystals synthesized with different DBSA dosages (A) Transmission electron microscope images of nanocrystals synthesized with different DBSA dosages. Scale bars, 30 nm. The insets show the corresponding size distribution histograms. (B) The dependences of nanocrystal size and chlorine-bromine ratio on DBSA dosage under the identical feeding ratio of other precursors. (C) XRD patterns of nanocrystals synthesized with different DBSA dosages. The right is an enlarged view belonging to the (200) plane.

Aiming to reveal the effects of DBSA on managing halogen activity within the system, a series of nanocrystals was synthesized under controlled conditions, only varying the amount of DBSA additive in the precursor solution. Pristine, DBSA 0.2, DBSA 0.4, DBSA 0.6, DBSA 0.8, and DBSA 1.0 refer to the nanocrystals synthesized without DBSA and with 0.2, 0.4, 0.6, 0.8, and 1.0 mL DBSA-toluene mixture (1 g mL^−1^ for DBSA), respectively. As shown in Figure 2A, the average size of the nanocrystals decreases with the increased amount of DBSA, ranging from approximately 10 to 6 nm on average (Figure 2B). This observation is consistent with the thermodynamic theory for the nucleation of colloidal particles. In this theory, the minimum size of nuclei that are stable to dissolution, known as the critical radius (*r*_c_), is given by^23^^,^^42^

$$r_{c}=\frac{2\gamma V_{m}}{N_{A}k_{B}T\;\ln\;S}\text{,}$$

where *N*_A_ is Avogadro’s number, *k*_B_ is the Boltzmann constant, *V*_m_ is the molar volume, and *T* is the temperature. The size of the formed nuclei is governed by the supersaturation (*S*) of precursor components and the surface energy (*γ*). The ultimate nanoparticle size is governed by the interplay between nucleation (controlled by *r*_c_) and subsequent growth kinetics (influenced by precursor diffusion and concentration). Specifically, a smaller *r*_c_ (high *S* or low *γ*) accelerates nucleation, generating numerous nuclei that exhaust precursors quickly, leading to limited growth and smaller final nanoparticle sizes. Conversely, a larger *r*_c_ (low *S* or high *γ*) reduces nucleation density, allowing abundant precursors to sustain the prolonged growth of individual nuclei, ultimately yielding larger nanoparticles. In this case, an increase in DBSA, which functions as a strong surface-binding ligand, reduces the surface energy and consequently leads to the formation of smaller nanocrystals. From a supersaturation perspective, the interaction between DBS^−^ and TOA^+^ elevates the supersaturation level of lead halide, a critical factor governing nanocrystal dimensions. This mechanism was validated in a pure bromide system, as illustrated in Figure S5. Increasing the amount of TOAB reduces the supersaturation of lead bromide, resulting in a redshift in the emission wavelength (Figures S5A and S5B), which indicates an increase in nanocrystal size. Conversely, when the TOAB concentration is held constant, an increase in DBSA raises the supersaturation level and induces a blueshift in the emission (Figures S5C and S5D), consistent with a reduction in nanocrystal size.

Besides the size regulation, the CsPb(Br_x_Cl_1__–__x_)_3_ nanocrystals exhibit significantly altered composition. The accurate chlorine and bromine content was measured using ion chromatography. Under the identical halogen feed ratio in the precursors, the measured chlorine-to-bromine molar ratio of the nanocrystals decreases from 1.16 to 0.27 when 1.0 mL of DBSA-toluene mixture (1 g mL^−1^ for DBSA) was introduced in the precursor solution. It presents a gradually decreasing trend with increasing DBSA (Figure 2B; Table S1). Spherical aberration-corrected STEM analysis (Figure S6) reveals lattice parameter consistency between core and surface regions at atomic resolution, demonstrating a uniform halogen distribution throughout the nanocrystal structure. Such a significant difference in the chlorine-to-bromine molar ratio undoubtedly stems from variations in the halogen ratio within the nanocrystals rather than alterations in surface composition induced by DBSA. The decreased chlorine content originates from the reduced activity of chloride ions after introducing DBSA, resulting in lattice expansion of the nanocrystal because of the discrepancy in the ionic radius of chlorine and bromine. XRD analysis indicates that nanocrystals are crystalline in the cubic phase. The peak position corresponding to the (200) plane of the nanocrystals shifts toward a lower angle with the increased amount of DBSA (Figure 2C). These results signify the remarkable effect of DBSA ligands on regulating the size and composition of nanocrystals, thereby influencing overall optoelectronic behaviors. Notably, as the nanocrystals exhibit weak quantum confinement, the composition dominates the emission wavelength regulation.

### Suppressing defects of nanocrystals synthesized with DBSA

**Figure 3:**
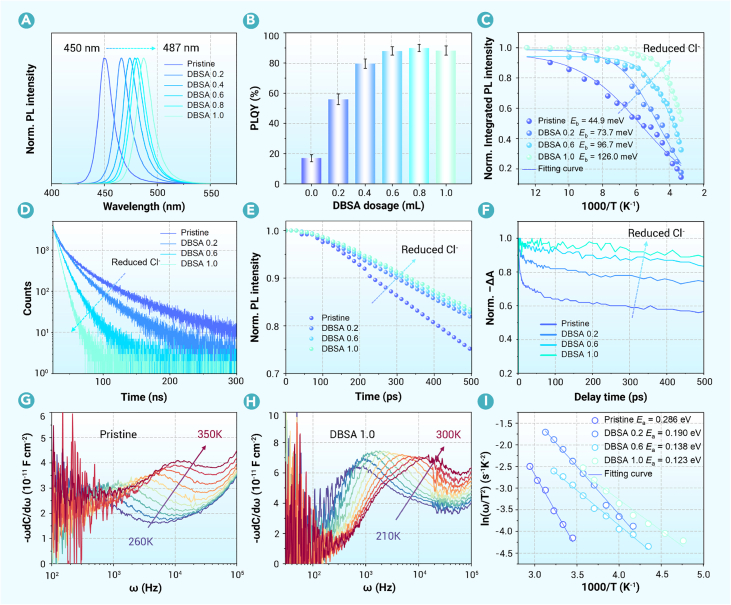
Optical behaviors of nanocrystals synthesized with different DBSA dosages (A) PL spectra. (B) PLQY of the nanocrystals synthesized with different DBSA dosages (DBSA added to toluene reaction precursor during nanocrystal synthesis before cesium precursor injection). (C) Temperature-dependence plots of integrated PL intensity vs. 1,000/T for extracting the *E*_b_ by fitting the Arrhenius equation. (D) Time-resolved PL decay spectra of the nanocrystals. (E) Normalized picosecond-resolution transient PL decay dynamics pumped with a 405 nm laser. (F) Normalized ground-state bleach kinetics extracted from TA spectra. Laser excitation: 405 nm, 100 kHz, 3.36 μJ cm^−2^. (G and H) Derivatives of temperature-dependent capacitance vs. frequency plots for devices based on nanocrystals synthesized with pristine (G) and DBSA 1.0 mL (H). (I) The defect activation energy fitted by the Arrhenius formula extracted from thermal admittance spectra.

Next, we investigated the optical properties of the nanocrystals with different sizes and compositions. While the reduction in nanocrystal size and the decrease in chlorine content exert opposing effects on the optical wavelength, the nanocrystal dimensions remain comparable to the exciton Bohr radius. Therefore, the halogen composition dominates the band-gap regulation. Consequently, with the increasing amount of DBSA additive in the precursors, both PL and UV-vis absorption spectra exhibit a substantial redshift and a strengthening of the first exciton absorption peaks (Figures 3A and S7).^43^ The emission peak shifts from deep blue (450 nm) to sky blue (487 nm), verifying broad-spectrum tunability via DBSA adjustments. Remarkably, the PLQY of the nanocrystals increases from less than 20% to around 90% with the increasing amount of DBSA (Figure 3B). However, excessive DBSA causes critically low halide activity, impairing nanocrystal quality during synthesis. The ligands on the surface of nanocrystals treated with various DBSA dosages were investigated through quantitative FTIR characterizations (Figure S8). The results show that an increase in DBSA dosage has a minor influence on the surface ligands of nanocrystals, excluding the large discrepancy in surface defects between the series of nanocrystals.

To understand the improved PLQY, we first conducted temperature-dependent PL measurements to analyze the exciton binding energy (*E*_b_) of nanocrystals. Four types of nanocrystals, referred to as pristine, DBSA 0.2, DBSA 0.6, and DBSA 1.0, were selected as the research objects to highlight the overall trend. From 80 to 300 K, the PL intensity of all nanocrystals exhibits a decreasing trend (Figure S9), correlating with non-radiative recombination resulting from thermally activated processes, such as exciton dissociation within nanocrystals.^44^ As the amount of DBSA increases, the decline in PL intensity decelerates. The *E*_b_ of nanocrystals can be estimated through fitting with the Arrhenius formula^45^:

$$I_{T}=\frac{I_{0}}{1+Aexp\left(-E_{b}/k_{B}T\right)}\text{,}$$

where *I*_*T*_ and *I*_0_ represent the integrated PL intensities at *T* and 0 K, respectively. *A* is a parameter associated with the cross-section of the binding state, and *k*_B_ stands for the Boltzmann constant. With the increased amount of DBSA, the *E*_b_ of the corresponding nanocrystals gradually increases (Figure 3C), consistent with the increase in the exciton absorption peak (Figure S7). Nevertheless, *E*_b_ is influenced by multiple factors, including nanocrystal sizes, composition, crystal structure, and surface characteristics, making it hard to establish a simple direct correlation between *E*_b_ and PLQY.

On the other hand, we observed a substantial PL redshift that contrasts with the expected size effect, attributed to the significantly reduced chlorine-to-bromine molar ratio in the nanocrystals. Therefore, the effect of reduced chlorine content on optical behaviors was further investigated. Transient PL and absorption experiments were conducted to study the carrier dynamics in CsPb(Br_x_Cl_1__–__x_)_3_ nanocrystals with reduced chlorine content. Transient PL decays at the tens-of-nanoseconds timescale show a shortened average lifetime when reducing the chlorine content (Figure 3D), seemingly contradictory to the improved PLQY. Detailed analysis of transient PL reveals two decay channels, which can be well-fitted by the following bi-exponential decay function:

$$I=I_{0}+A_{1}\;\exp\left(-t/\tau_{1}\right)+A_{2}\;\exp\left(-t/\tau_{2}\right)\text{,}$$

where *I*_0_ is a constant; the extracted decay lifetimes (*τ*_1_ and *τ*_2_) and the corresponding proportionality coefficients (*A*_1_ and *A*_2_) are shown in Figure S10. *τ*_1_ shows a decrease from 12 to 6 ns with the increased DBSA (reduced chlorine content), while *τ*_2_ is found to be largely dependent on the DBSA, decreasing from 61 to 19 ns when mixing 1 mL of DBSA in the reactant. The proportionality coefficients of the fast decay channel (*A*_1_) increase, and the slow decay channel (*A*_2_) decreases with the increased DBSA. The excitation power density-dependence test of PL lifetime (Figure S11) shows that the PL lifetimes (both *τ*_1_ and *τ*_2_ components) remain constant within the tested power range (0.1–2.0 μJ cm^−2^). This independence strongly suggests that the dominant recombination mechanisms are monomolecular processes (exciton radiative recombination and defect-assisted recombination) rather than bimolecular or Auger processes, which would exhibit power-dependent lifetimes. In typical nanocrystals, previous research shows that the fast decay channel is usually considered the intrinsic excitonic emission and the slow one is related to trap-assisted recombination.^46^^,^^47^ This differs from the bulk films, in which no excitonic emission was observed, and a longer decay lifetime is normally associated with a higher PLQY.^48^^,^^49^ The shorter *τ*_2_ and decreased *A*_2_ for the nanocrystals synthesized with increased DBSA infer weaker carrier trapping and de-trapping processes. Transient PL measurements with picosecond resolution were conducted to validate the carrier-trapping process. The nanocrystals with reduced chlorine content exhibit slower PL decay on the hundred-picosecond timescale, namely, a weaker carrier-trapping contribution (Figure 3E). Furthermore, the TA decay kinetics of the nanocrystals show much slower ground-state bleach decay at the several-tens-of-picoseconds timescale under weak excitation conditions (Figures 3F and S12), verifying that more carriers remain at the band edge instead of being trapped by defects.^50^^,^^51^ The excitation power-density-dependent PLQY measurements also support fewer carrier-trapping processes in the nanocrystals with reduced chlorine content (Figure S13).

To further pinpoint defect-state information in the nanocrystal layer, we conducted temperature-dependent admittance spectroscopy (TAS) on devices containing nanocrystals with different DBSA dosages.^30^ The admittance spectra and derivative plots (−ωdC/dω vs. ω) measured at various temperatures are shown in Figures 3G, 3H, and S14. Distinct steps observed in the capacitance spectra across different temperatures correspond to carrier release and capture rates at defect states within the band gap. These characteristic defect-related responses shift toward higher frequencies with increasing temperature. By performing Arrhenius fitting, we determined the activation energy of these defects. The extracted defect-state distribution and activation energies from TAS measurements are presented in Figures 3I and S15. The results demonstrate that increasing DBSA content reduces the defect activation energy (*E*_a_) from 0.286 (pristine) to 0.123 (DBSA 1.0 mL) eV, and the corresponding integrated tDOS decreases from 2.67 × 10^15^ to 6.47 × 10^14^ cm^−3^. SCLC measurements on hole-only devices further support the DBSA-mediated reduction in defect-state density (Figure S16).^52^

It is important to clarify that the defects in perovskite nanocrystals are not exclusively surface localized. The chlorine-to-bromine molar ratio of nanocrystals decreases largely from 0.414 to 0.166 after introducing DBSA in the purified nanocrystals shown in Figure 1C, corresponding to at least a 17.5% chlorine deficiency relative to total halogens. However, the total surface halogen ratio in a nanocrystal with dimensions of 7 nm is calculated to be ∼15%. This confirms that defects in perovskite nanocrystals cannot be exclusively surface localized. Internal halogens can migrate to replenish surface vacancies, thereby generating internal defects. We infer that those nanocrystals contain both surface and internal defects, which are maintained in a dynamic equilibrium via processes such as ion migration. Besides, the DBSA-synthesized nanocrystals show nearly identical ligand coverage (Figure S8) while exhibiting improved PLQY when increasing the DBSA dosage from 0.2 to 0.6 mL, which primarily stems from suppressing chlorine-related internal defects.

### Electroluminescence performance of the nanocrystals

**Figure 4:**
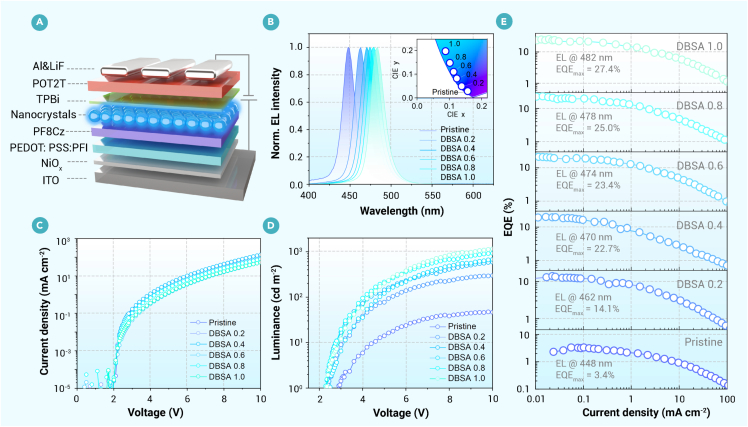
Electroluminescence performance of the LEDs based on nanocrystals synthesized with different DBSA dosages (A) Schematic of the device structure, showing the multilayers of indium tin oxide (ITO; ∼60 nm), NiO_x_ (∼3 nm), PEDOT:PSS:PFI (∼35 nm), PF8Cz (∼25 nm), CsPb(Br_x_Cl_1__–__x_)_3_ nanocrystal monolayer (∼6–10 nm), TPBi (∼5 nm), PO-T2T (∼45 nm), LiF (∼1 nm), and aluminum (Al; ∼100 nm). (B) Normalized EL spectra of the LEDs, with an inset of corresponding CIE color coordinates. The relatively small current density of the LEDs is caused by the insulating PFI. (C and D) Typical current density-voltage (C) and luminance-voltage (D) characteristics of the LEDs. (E) EQE-current density curves of the LEDs.

LEDs consisting of multilayer structures (Figure 4A) were fabricated to investigate the electroluminescence performance of the nanocrystals. In this structure, precisely controlled CsPb(Br_x_Cl_1__–__x_)_3_ nanocrystal monolayers were deposited as the emissive layer to converge carrier injection while avoiding the high-resistance carrier-transporting process in the nanocrystal film. The nickel oxide interlayer, combined with the PFI-doped PEDOT:PSS, ensures efficient hole injection. The multilayer hole transport layer (HTL) and electron transport layer (ETL) are integrated to balance carrier injection and confine excitons, aiming to achieve high-performance blue perovskite nanocrystal LEDs with high EQE.

All the nanocrystals exhibit stable electroluminescence (EL) with emission peaks centered at 448, 462, 470, 474, 478, and 482 nm, with the increased amount of DBSA from 0 to 1 mL (Figure 4B), corresponding to the CIE chromatic coordinates of (0.157, 0.028), (0.138, 0.049), (0.123, 0.082), (0.113, 0.110), (0.102, 0.147), and (0.088, 0.196), respectively. The typical current density-luminance-voltage curves of those devices are shown in Figures 4C and 4D, illustrating similar current density-voltage characteristics and nearly identical turn-on voltages of 2.3–2.5 V. The result suggests analogous carrier injection and transport behaviors, consistent with the comparable band structure of the CsPb(Br_x_Cl_1__–__x_)_3_ nanocrystals measured from the UV photoelectron spectroscopy (UPS) characterizations (Figure S17). Interestingly, the turn-on voltage of the device is lower than the emitted photon energy. This phenomenon originates from the synergistic effects of the unique electron-phonon coupling properties of perovskite and balanced carrier injection in the device.^53^^,^^54^ Notably, the luminance of the LEDs was enhanced significantly under a constant voltage for the nanocrystals synthesized with the increased amount of DBSA, even after considering the luminance calibration with human visual function (Figure S18). Consequently, the maximum EQEs reached 3.4% (448 nm), 14.1% (462 nm), 22.7% (470 nm), 23.4% (474 nm), 24.0% (478 nm), and 27.4% (482 nm), increasing monotonically with the reduced chlorine content (Figure 4E). The results indicate that the electroluminescence performance of nanocrystals is closely related to deep-level defects associated with chlorine. Given that the surface ligands of the nanocrystals are nearly identical, their electroluminescence behavior is primarily governed by the defects modulated by DBSA.

**Figure 5:**
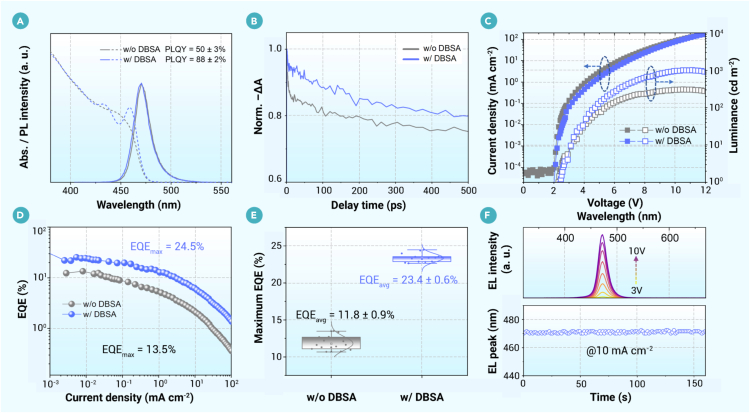
The performance comparison of the nanocrystals at an identical emission (470 nm), synthesized with or without DBSA (A) Absorption and PL spectra of the two nanocrystals. (B) Normalized ground-state bleach kinetics extracted from TA spectra. Laser excitation: 405 nm, 100 kHz, 3.36 μJ cm^−2^. (C and D) Typical current density-voltage-luminance characteristics (C) and EQE-current density curves of the LEDs (D) based on the two nanocrystals. (E) Maximum EQE statistics of LEDs (15 devices each). (F) Voltage-dependent EL spectra and time-dependent EL peak of the LEDs based on DBSA-nanocrystal.

To exclude the influence of varying energy-level alignment resulting from different nanocrystal band gaps and device structures, and thereby highlight the critical role of defect suppression in enhancing device performance, two representative CsPb(Br_x_Cl_1__–__x_)_3_ nanocrystals with the emission peak centered at 470 nm were synthesized with or without the inclusion of DBSA, accompanied by adjusting the feeding amounts of precursors (Table S2). The surface defects of the nanocrystals synthesized without DBSA can be effectively passivated with DDA^+^ and ETBPB additives. The absorption and PL spectra of the two nanocrystals are shown in Figure 5A, where the nanocrystal synthesized with DBSA (DBSA-nanocrystal) exhibits a strong exciton absorption peak, attributed to the reduction in size and the improvement in size uniformity. In terms of optical properties, the DBSA-nanocrystal presents much slower ground-state bleach decay in the TA decay kinetics (Figures 5B and S19) and a shorter average PL lifetime at the tens-of-nanoseconds timescale (Figure S20), verifying fewer defects generated under the DBSA ligand-participating synthesis. In electroluminescence performance, the maximum luminance reaches 1,000 cd m^−2^ for the LEDs based on the DBSA-nanocrystal, much brighter than the device based on the control nanocrystal (Figure 5C). Regarding efficiency, the maximum EQE of the champion LEDs based on the DBSA-nanocrystal reaches 24.5% (Figure 5D), representing the highest value achieved for pure-blue emission in perovskite LEDs to date (Figure S21). In statistics, the histogram of the peak EQEs shows an average EQE of 23.4% with a small deviation of 0.56% (Figure 5E), two times higher than the average EQE of the counterpart devices. A maximum EQE of 24.0% was also certified through third-party certification (Figure S22), comparable to that measured in the laboratory. The corresponding EQE values at 100 and 1,000 cd m^−2^ are approximately 15% and 2%, respectively. Notably, the operational stability of the devices shows significant improvement (Figure S23). Spectral stability is maintained even under extreme conditions, as evidenced by invariant emission peaks at 470 nm when increasing the driving voltage to 10 V or under continuous driving (Figure 5F). Furthermore, reducing Cl content in precursors to mitigate Cl defects during the nucleation process and subsequent Cl supplementation with DDAC constitutes a promising approach for nanocrystal synthesis. The corresponding LEDs also demonstrate high peak EQEs (Figure S24). However, excessive quaternary ammonium ligands (DDA^+^) may induce more pronounced efficiency roll-off in the devices and worse operational stability (Figure S25). The superiority in optical behavior and device performance of the DBSA-nanocrystal compellingly demonstrates the advancement of ligands in modulating the surface and internal defects of nanocrystals.

## Conclusion

In summary, we have demonstrated the surface-ligand-triggered synthetic control of defects in nanocrystals. The strong acid ligand DBSA ionizes to DBS^−^ and H^+^ in low-polarity solvents and interacts with quaternary ammonium and halogens, respectively, thereby modulating the activity of halogen. This unveils a new functionality of acid ligand, which allows for controllable regulation of the nanocrystal composition, and thus suppresses the formation of halogen-related internal defects. Leveraging these insights, blue-emissive nanocrystals centered at 470 nm achieve a PLQY of ∼90% and yield blue perovskite LEDs with champion EQEs of up to 24.5%, setting a new benchmark for pure-blue perovskite LEDs. Further investigations into surface ligands for controlling nanocrystal surfaces and internal defects are expected to offer exciting possibilities in advanced optoelectronic applications.

## Resource availability

### Materials availability

All materials used in this study are commercially available or can be synthesized according to the procedures described in the materials and methods.

### Data and code availability

The authors declare that the main data supporting the findings of this study are available within the paper and its supplemental information. Extra data are available upon reasonable request from the corresponding author.

## Funding and acknowledgments

This work was financially supported by the “Pioneer” and “Leading Goose” R&D Program of Zhejiang (2024C01191, X.D.), the 10.13039/501100012226Fundamental Research Funds for the Central Universities (2024QZJH10, X.D.), and the 10.13039/100014717National Natural Science Foundation of China (U22A20133, Z.Y.). X.D. gratefully acknowledges the support of the Zhejiang University Education Foundation Qizhen Scholar Foundation.

## Author contributions

X.D. and Q. Cao conceived the idea and designed the experiments. X.D. supervised the work. Q. Cao synthesized the nanocrystals, fabricated the device, and performed optical measurements. Y.F. and H.Z. assisted in composition and crystal structure characterizations. Q. Cai and Y.G. assisted in the synthesis of nanocrystals and optical measurements. D.Z. and Y.H. assisted in the device fabrication and characterization. X.W. assisted in the temperature-dependent PL measurements. M.Z., H.H., and Z.Y. provided helpful discussions. Q. Cao wrote the original draft of the manuscript. X.D. revised the manuscript. All authors contributed to the manuscript and approved the final version.

## Declaration of interests

The authors declare no competing interests.

## Supplemental information

Document S1. Figures S1–S25 and Tables S1 and S2

Document S2. Article plus supplemental information
