# Supplementary material for: Surface-ligand-triggered synthetic control of defects in nanocrystals toward high-efficiency blue electroluminescence
Source: Innovation (Camb). 2026 Jan 20;7(6):101273. doi: 10.1016/j.xinn.2026.101273 (PMC13237842; doi:10.1016/j.xinn.2026.101273)
Supplement: Document S1. Figures S1–S25 and Tables S1 and S2 [file mmc1.pdf]

The Innovation, Volume 7

Supplemental Information

Surface-ligand-triggered synthetic control of defects in nanocrystals toward high-efficiency blue electroluminescence

Qingli Cao, Qiuting Cai, Yifeng Feng, Xinyang Wang, Dingshuo Zhang, Yun Gao, Haoran Zhang, Meiyi Zhu, Yifan He, Haiping He, Zhizhen Ye, and Xingliang Dai

SUPPLEMENTAL INFORMATION

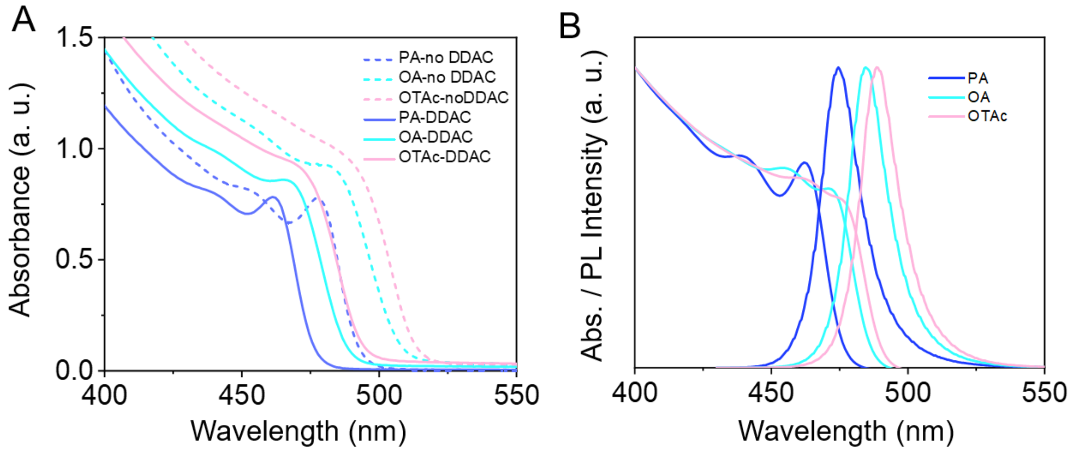

Figure S1. (A) Absorption spectra of nanocrystal crude solution before and after DDAC treatment. The nanocrystals were synthesized using cesium precursors dissolved in different acids (OA = oleic acid, OTAc = octanoic acid). (B) Absorption and PL spectra of purified nanocrystals using different acids under the same chlorine-bromine feeding ratio.

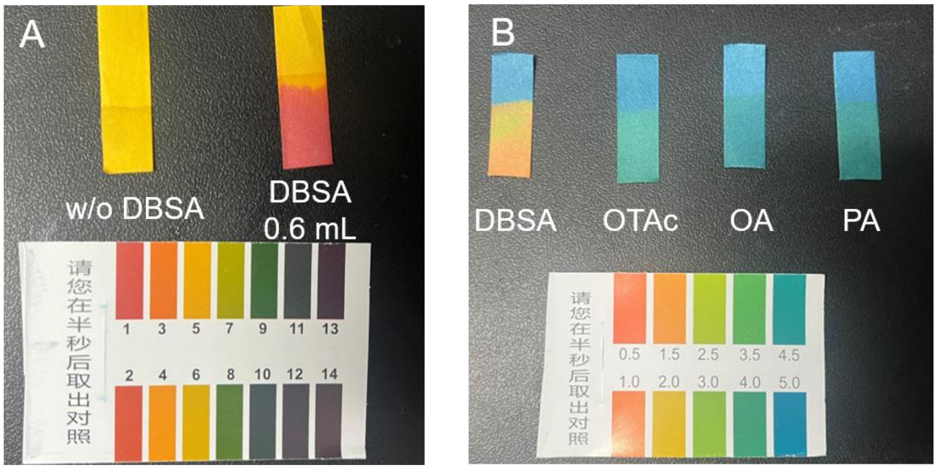

Figure S2. (A) Comparison of pH values before and after adding DBSA (1 g mL^{-1}) to the precursor solution. (B) Comparison of pH values of different acids diluted in toluene (0.2 M).

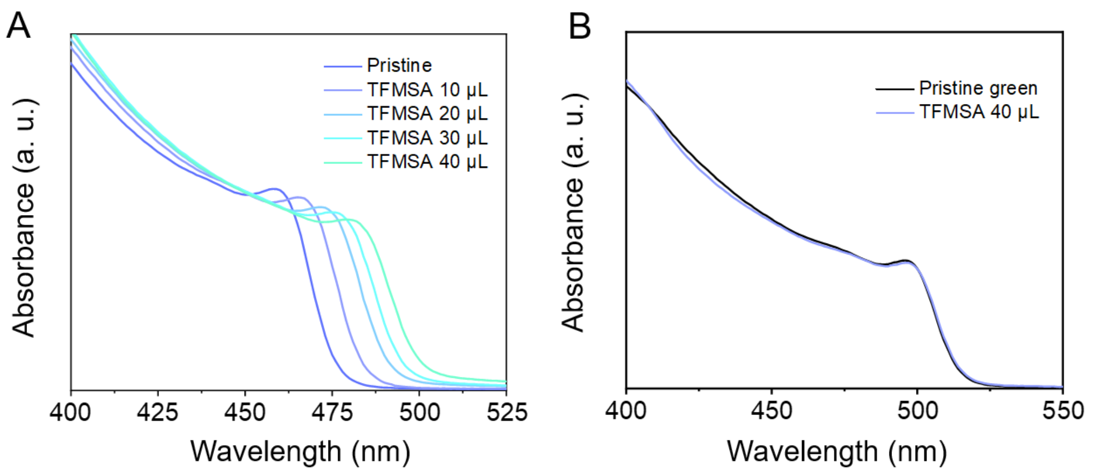

Figure S3. (A) Absorption spectra of different amounts of TFMSA added to the crude $CsPb(Br_xCl_{1-x})_3$ nanocrystal solution without purification. (B) Absorption spectra of crude $CsPbBr_3$ nanocrystal solution without purification before or after inclusion of 40 μL TFMAS.

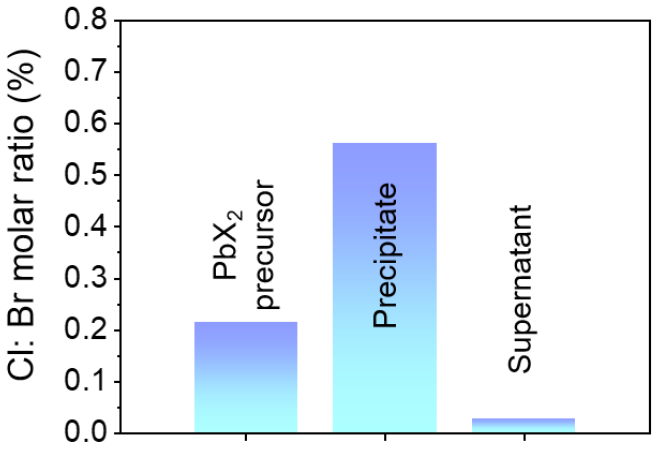

Figure S4. The chlorine-bromine ratio of the precursor solution, precipitate, and supernatant measured by ion chromatography.

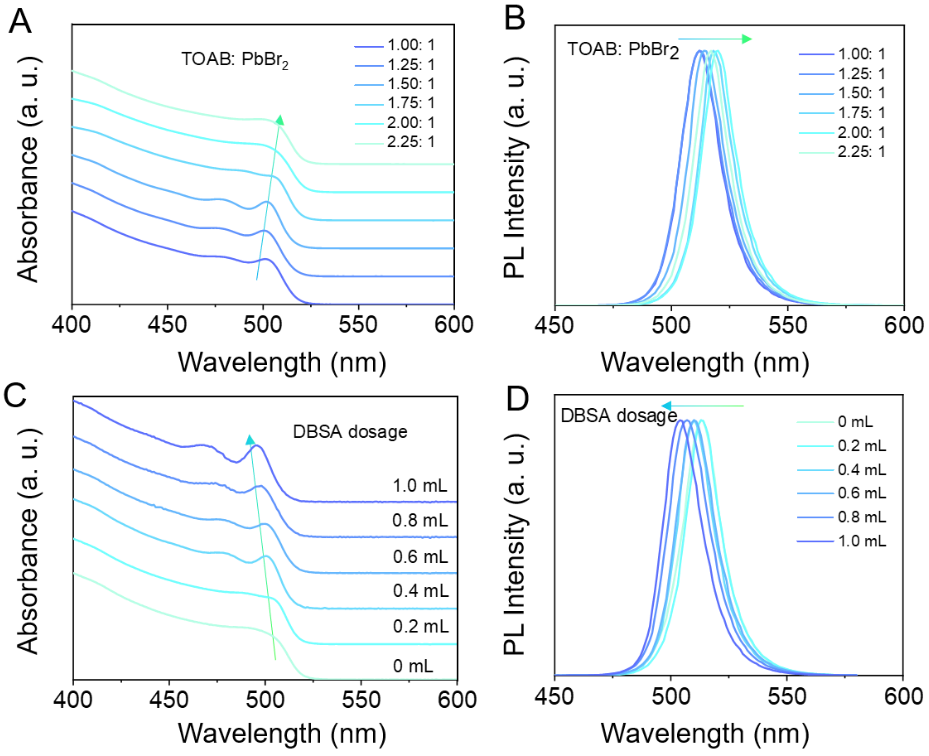

Figure S5. (A, B) Absorption and PL spectra of $CsPbBr_3$ nanocrystals synthesized with different TOAB dosages (DBSA dosage = 0.4 mL). (C, D) Absorption and PL spectra of $CsPbBr_3$ nanocrystals synthesized with different DBSA dosages (TOAB: $PbBr_2$ = 1: 1.5).

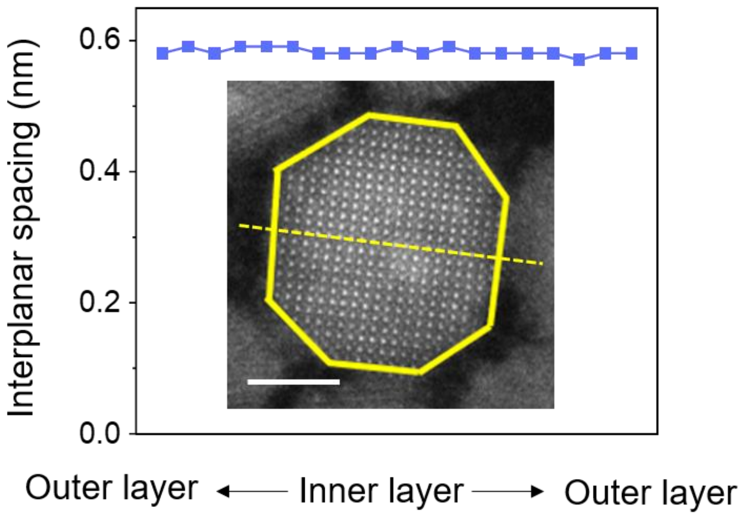

Figure S6. Interplanar spacing as a function of layer position in the crystal lattice of a nanocrystal. The insets correspond to a spherical aberration-corrected HAADF-STEM image of a nanocrystal. Scale bar: 5 nm.

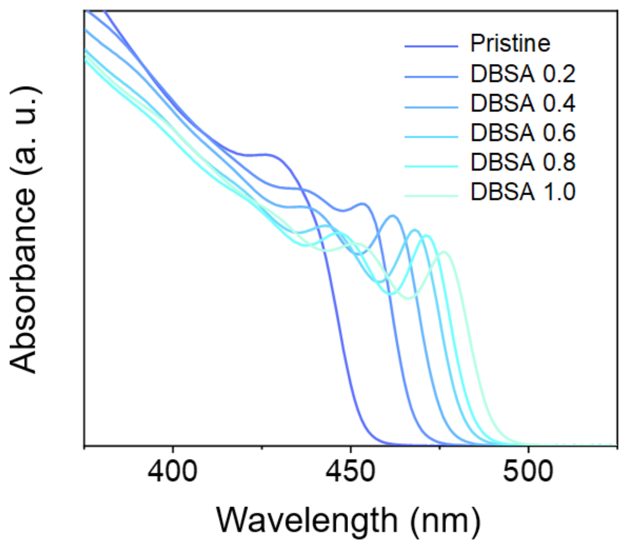

Figure S7. Absorption spectra of nanocrystals synthesized with different DBSA dosages added to the precursor solution before Cs-PA triggered the reaction.

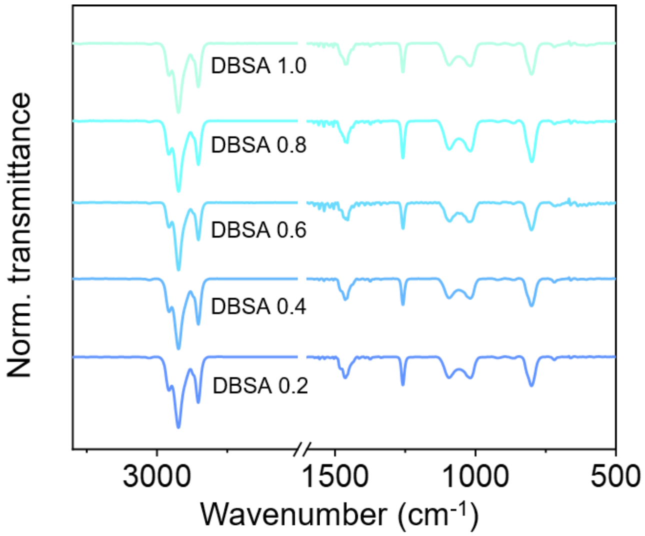

Figure S8. Fourier transform infrared spectra of the nanocrystals synthesized with different DBSA dosages.

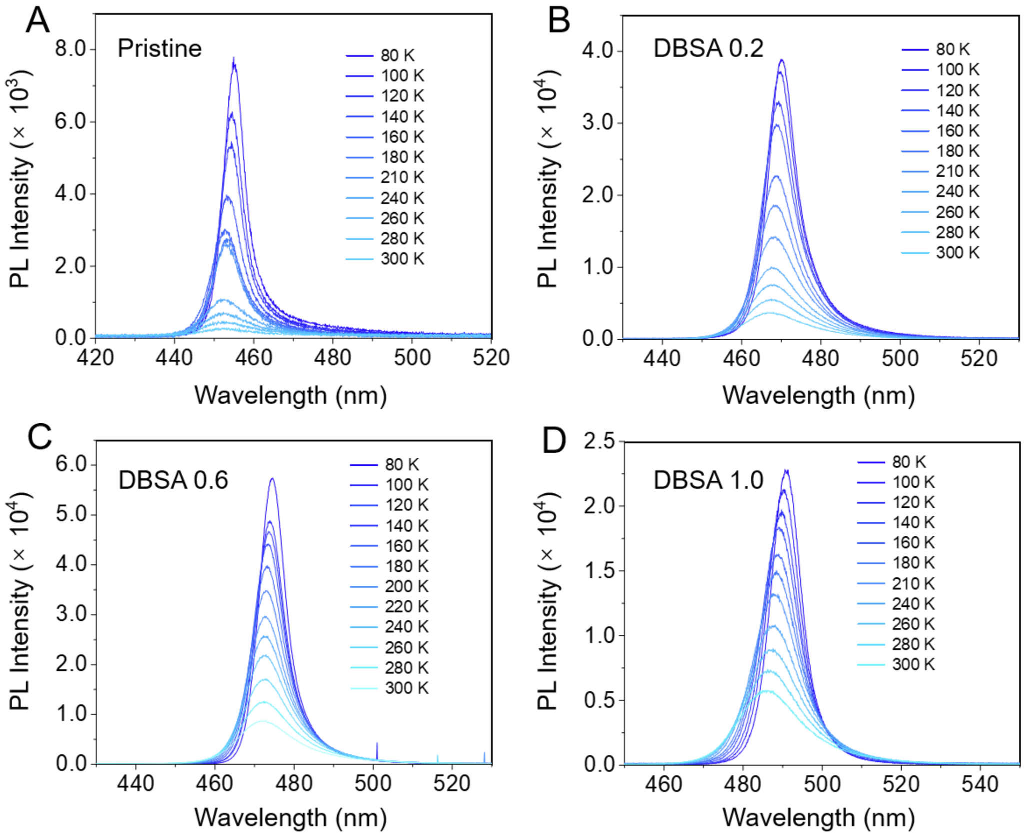

Figure S9. Temperature-dependent PL spectra of nanocrystals synthesized with different DBSA dosages from 80 K to 300 K with an excitation power density of about 5 mW cm^{-1}: pristine (A), DBSA 0.2 ml (B), DBSA 0.6 ml (C), DBSA 1.0 ml (D).

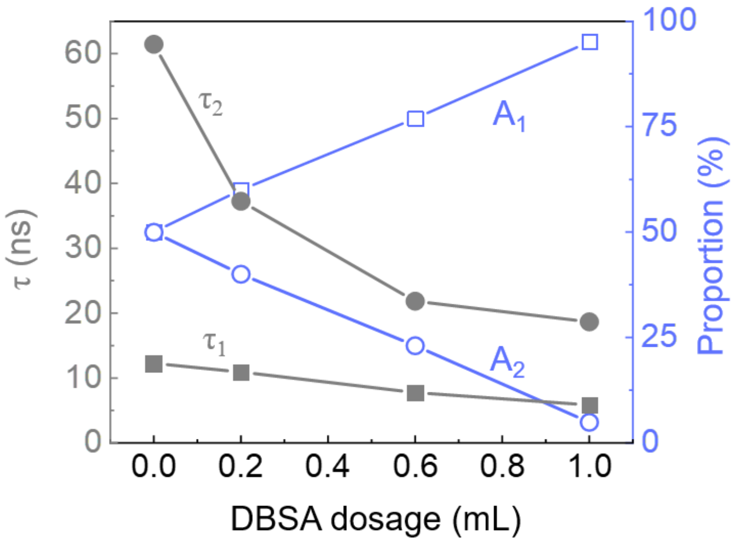

Figure S10. The extracted lifetimes (τ_1, τ_2) and proportions (A_1, A_2) fitted with a double exponential function from time-resolved PL decay spectra of nanocrystals synthesized with different DBSA dosages.

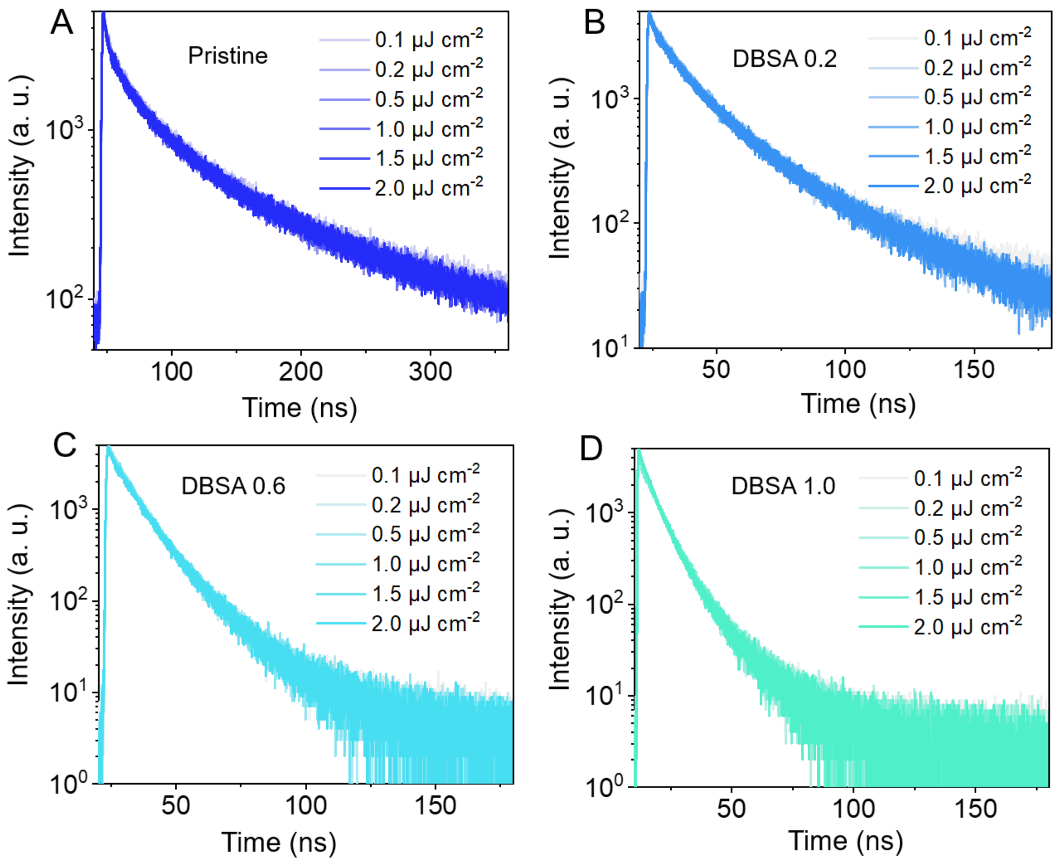

Figure S11. The excitation power density-dependent transient PL decay of the nanocrystals synthesized with different DBSA dosages: pristine (A), DBSA 0.2 mL (B), DBSA 0.6 mL (C), DBSA 1.0 mL (D).

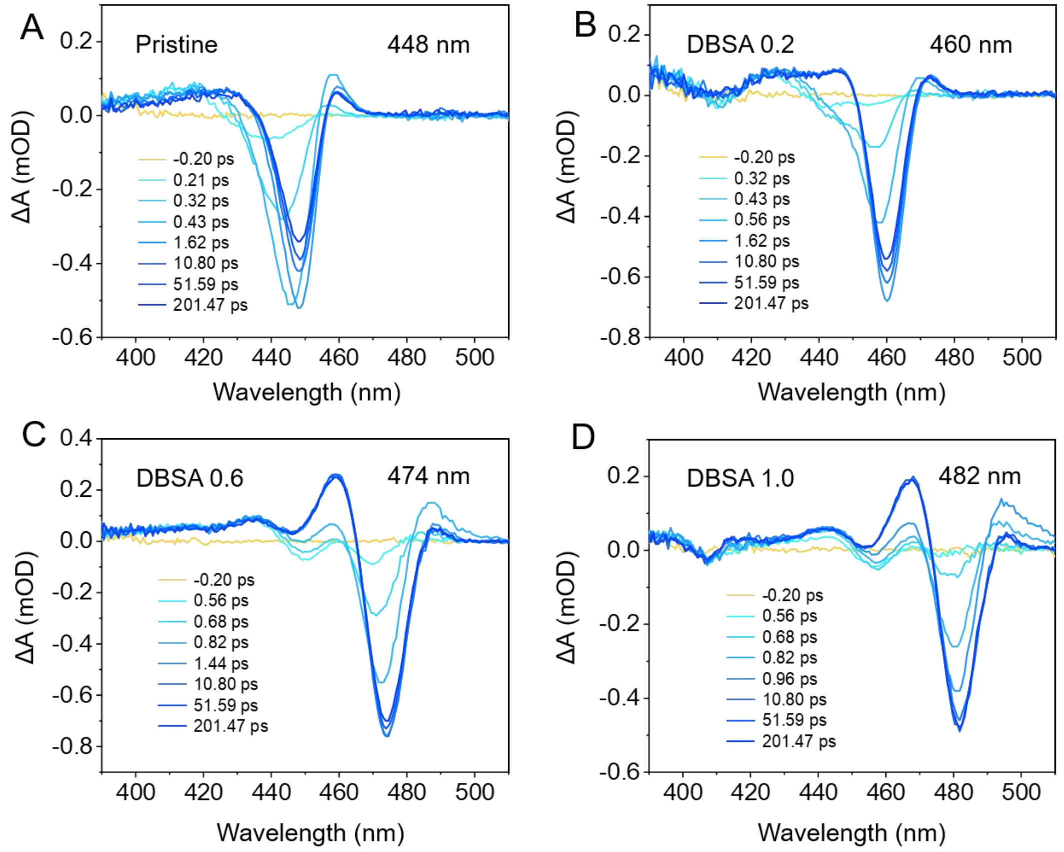

Figure S12. The typical transient absorption spectra of nanocrystals synthesized with different DBSA dosages: pristine (A), DBSA 0.2 mL (B), DBSA 0.6 mL (C), DBSA 1.0 mL (D).

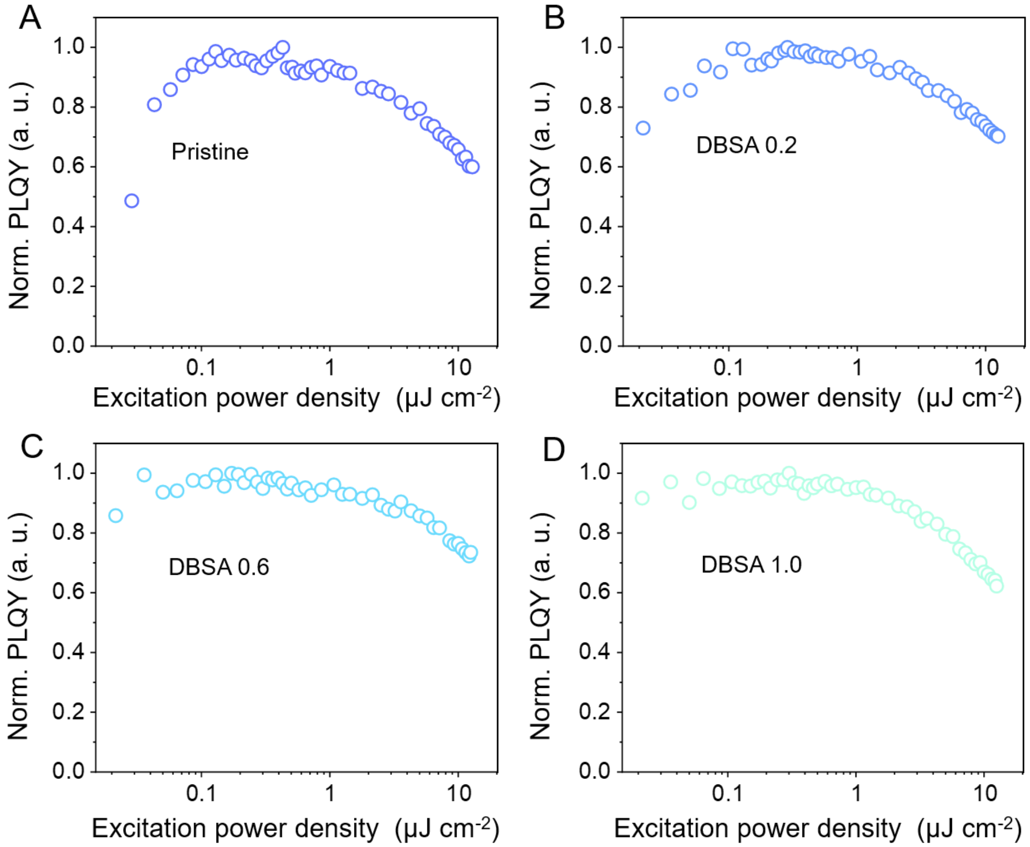

Figure S13. PLQY excitation power density dependence of nanocrystals with varying DBSA dosages: pristine (A), DBSA 0.2 mL (B), DBSA 0.6 mL (C), DBSA 1.0 mL (D). At low excitation power, the PLQY of the pristine sample increases with power density due to defect-state filling. Low carrier concentrations initially lead to defect-assisted recombination dominance (low PLQY), but higher power saturates defects, enhancing excitonic recombination and PLQY, indicating high defect density of pristine nanocrystals. This effect weakens with increased DBSA dosage, confirming defect passivation by DBSA. In intermediate power ranges, stable PLQY independent of excitation power across samples suggests excitonic recombination dominance,[1] aligned with large exciton binding energy of nanocrystals. While at high power, PLQY declines, which reflects Auger recombination dominance.

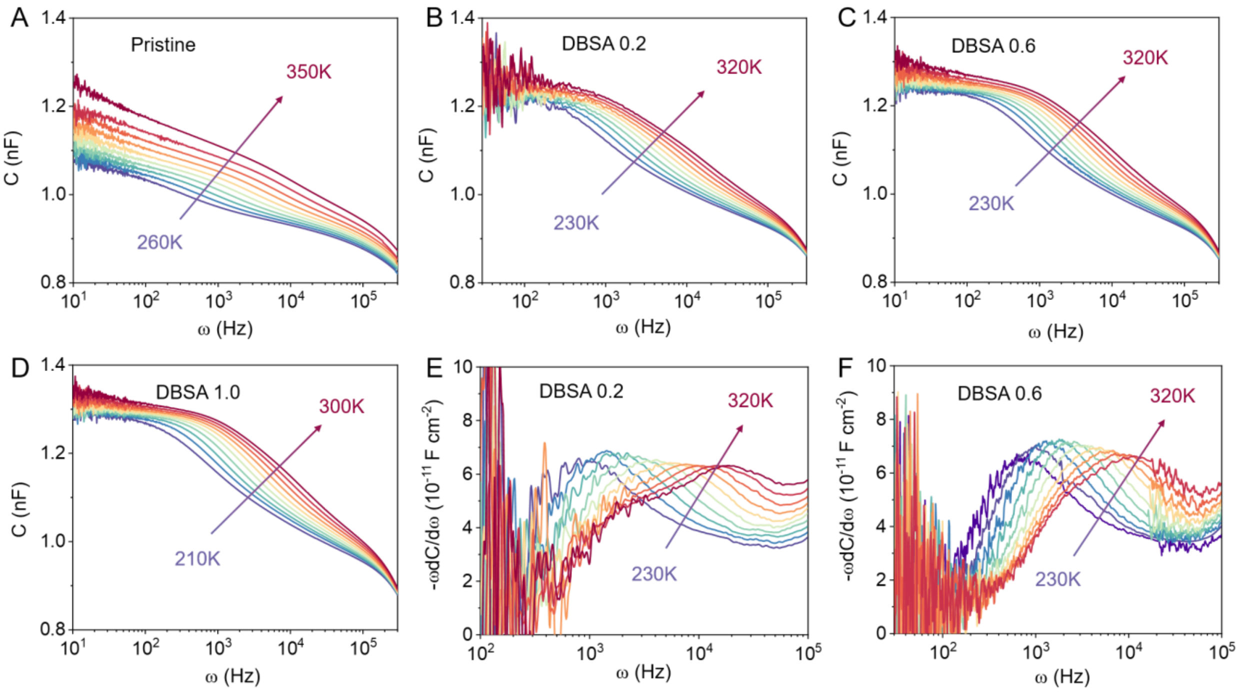

Figure S14. Temperature-dependence of capacitance versus frequency plots for devices based on nanocrystals synthesized with different DBSA dosages: pristine (A), DBSA 0.2 mL (B), DBSA 0.6 mL (C), DBSA 1.0 mL (D). Derivatives of temperature-dependent *C*-*f* plots for devices based on nanocrystals synthesized with DBSA 0.2 mL (E) and 0.6 mL (F).

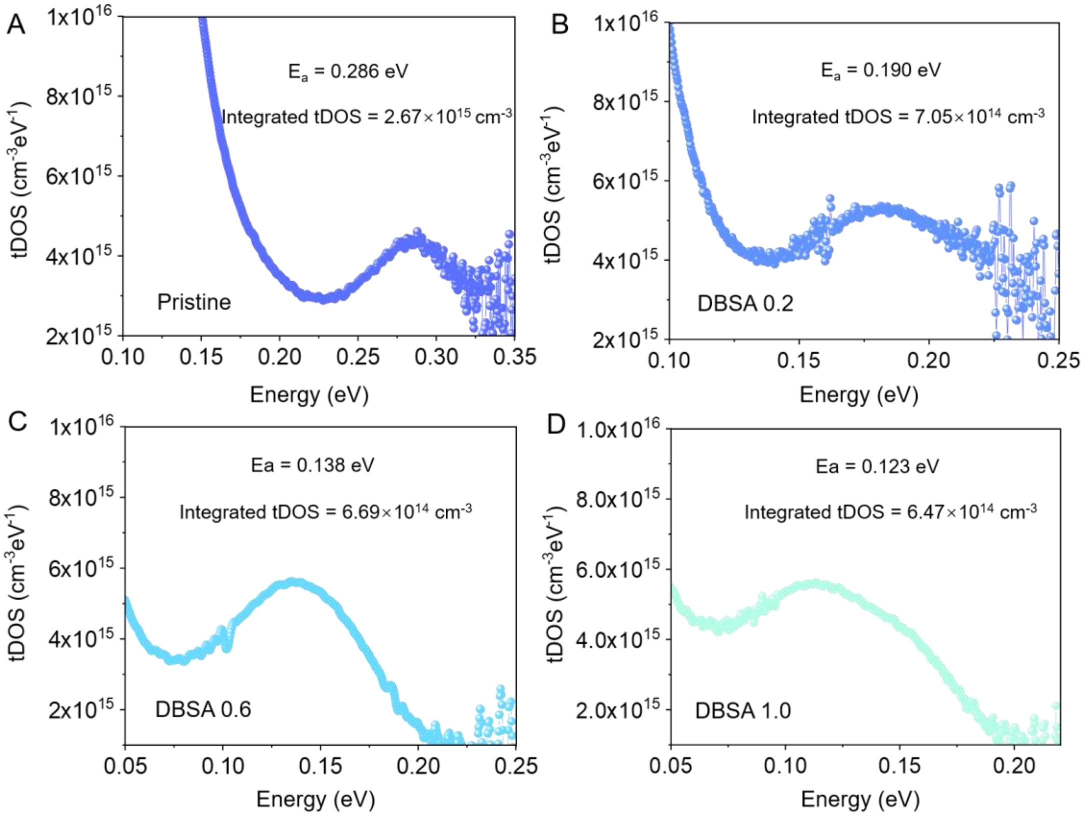

Figure S15. Density of defect states extracted from thermal admittance spectra for nanocrystals synthesized with different DBSA dosages: pristine (A), DBSA 0.2 mL (B), DBSA 0.6 mL (C), DBSA 1.0 mL (D).

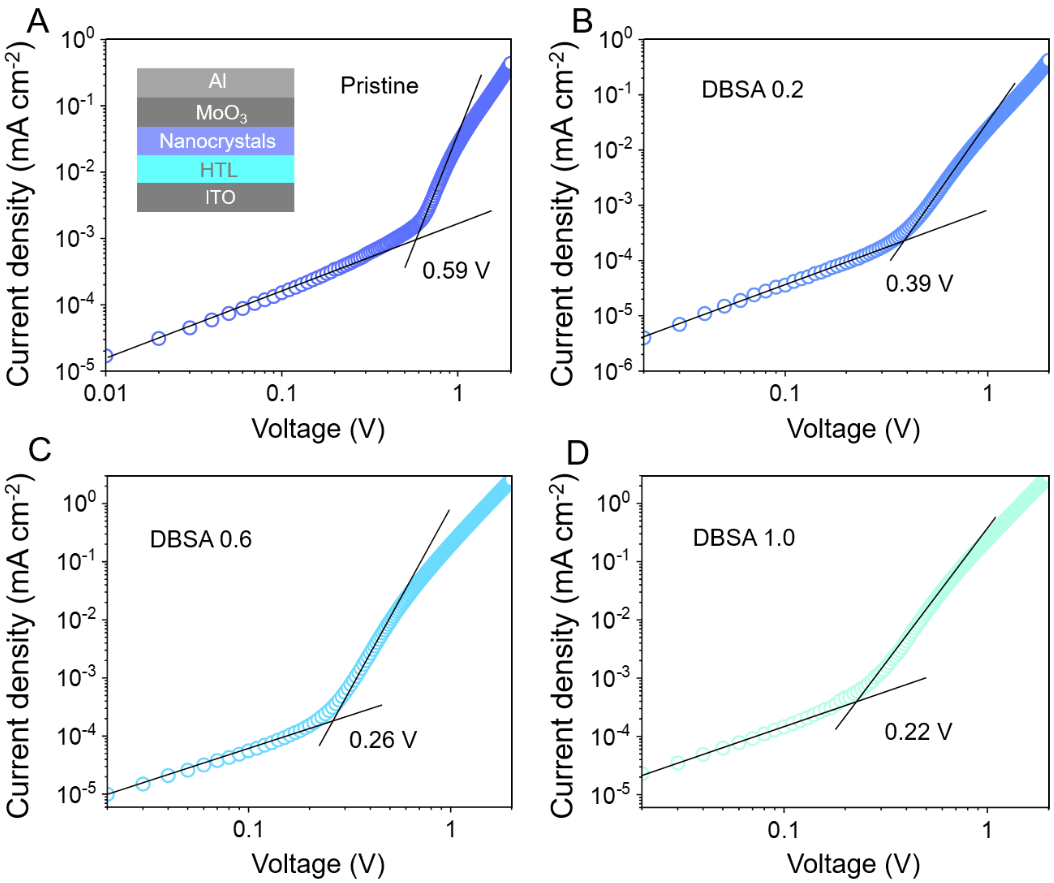

Figure S16. Current density-voltage curves of hole-only devices based on nanocrystals synthesized with different DBSA dosages: pristine (A), DBSA 0.2 mL (B), DBSA 0.6 mL (C), DBSA 1.0 mL (D). The inset shows the corresponding device structures. The extracted trap-filled limit voltages (V_{TFL}) were 0.59 V (Pristine), 0.39 V (DBSA 0.2 mL), 0.26 V (DBSA 0.6 mL), and 0.22 V (DBSA 1.0 mL). The calculated trap state densities derived from these measurements were 2.33×10^{17} cm^{-3}, 1.95×10^{17} cm^{-3}, 1.48×10^{17} cm^{-3}, and 1.25×10^{17} cm^{-3}, respectively.

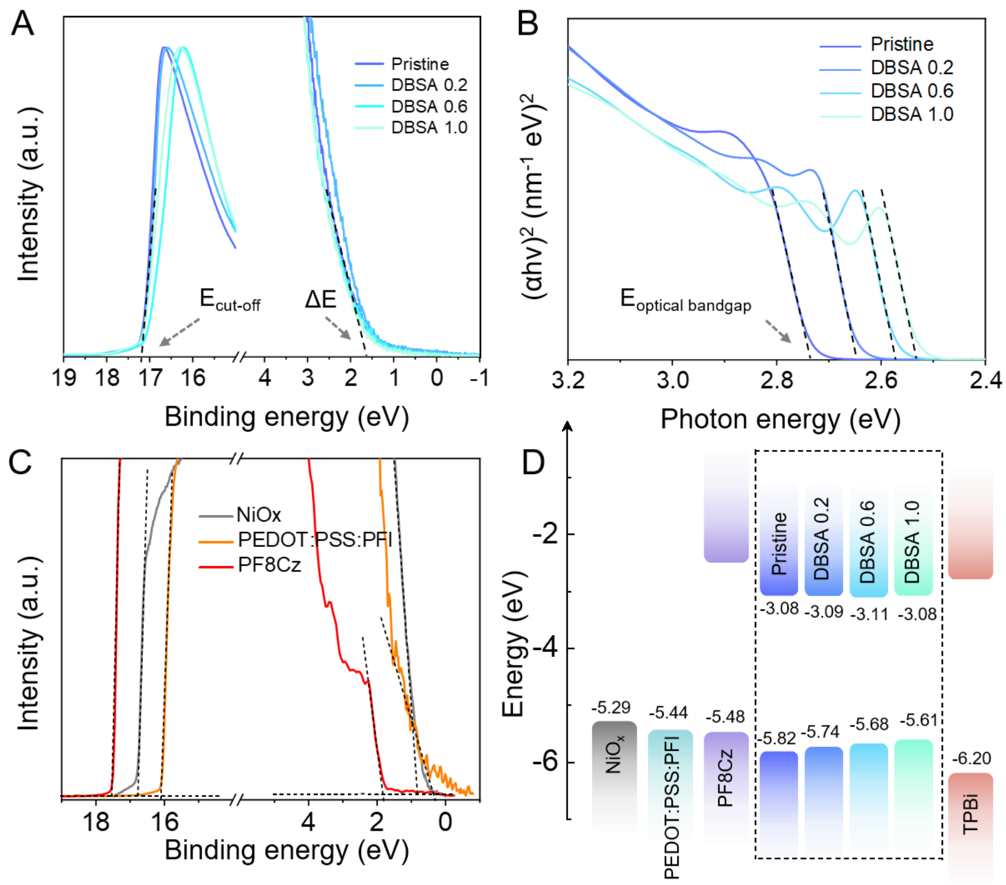

Figure S17. (A) UPS spectra of nanocrystals with different DBSA dosages: Photoemission cut-off region (left) and the valence-band-edge region (right). (B) The plots of $(\alpha h\nu)^2$ versus the photon energy calculated from the absorption measurement. (C) UPS spectra of NiO_x, PEDOT:PSS:PFI, and PF8Cz. (D) Flat-band energy level diagram. The black dashed outline highlights the flat band energy level of nanocrystals with different DBSA dosages. E_{VB} and E_{CB} are calculated with the formula: $E_{VB} = E_{cut\text{-}off} - 21.2 - \Delta E$; $E_{CB} = E_{optical\ bandgap} + E_{VB}$.

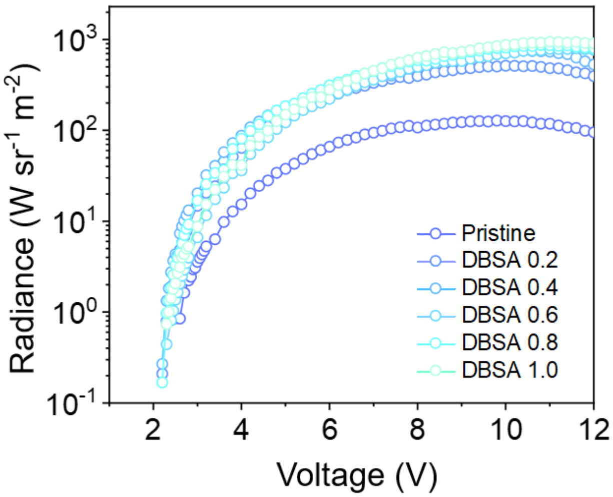

Figure S18. The radiance-voltage curves of the LEDs based on nanocrystals synthesized with different DBSA dosages.

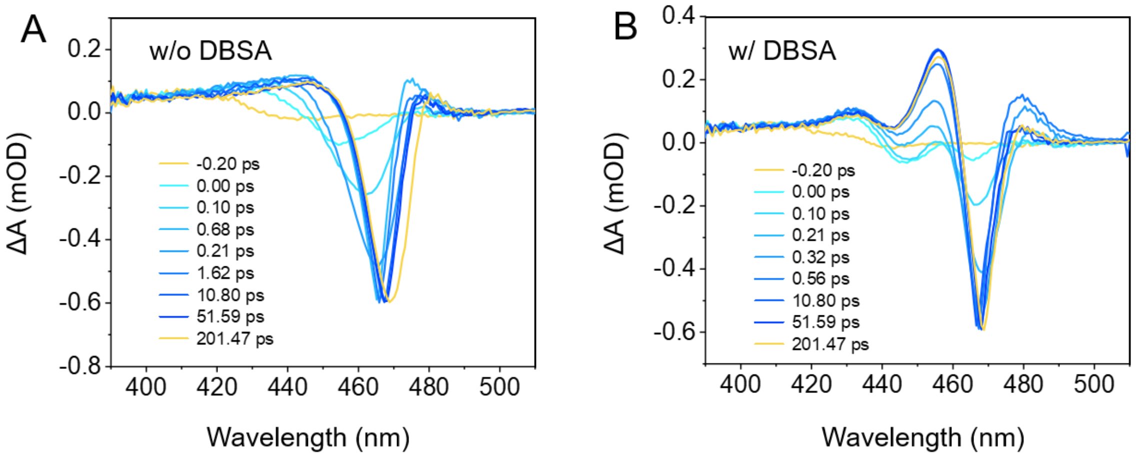

Figure S19. The typical transient absorption spectra of nanocrystals with (A) or without (B) DBSA treatment at the same emission wavelength centred at 470 nm.

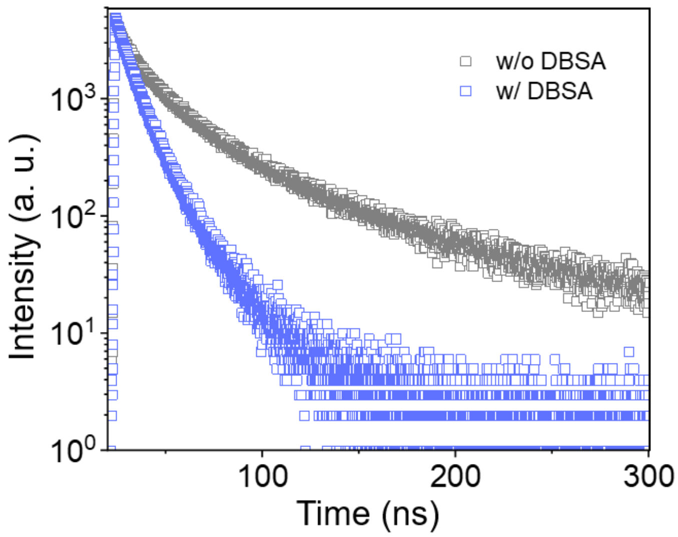

Figure S20. TRPL decay spectra of nanocrystals with or without DBSA treatment at the same emission wavelength centred at 470 nm.

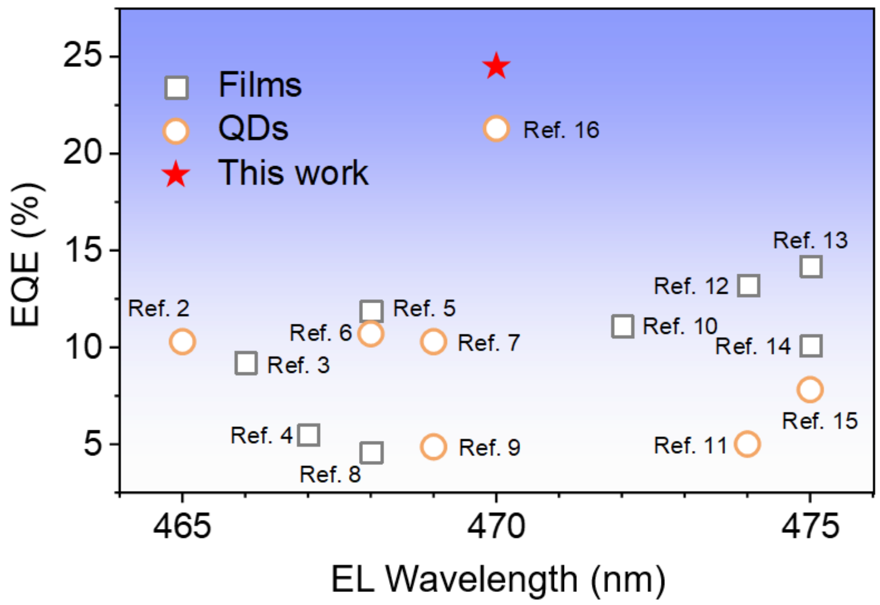

Figure S21. Reported peak EQE and EL wavelength of blue LEDs based on perovskite films or nanocrystals.[2-16]

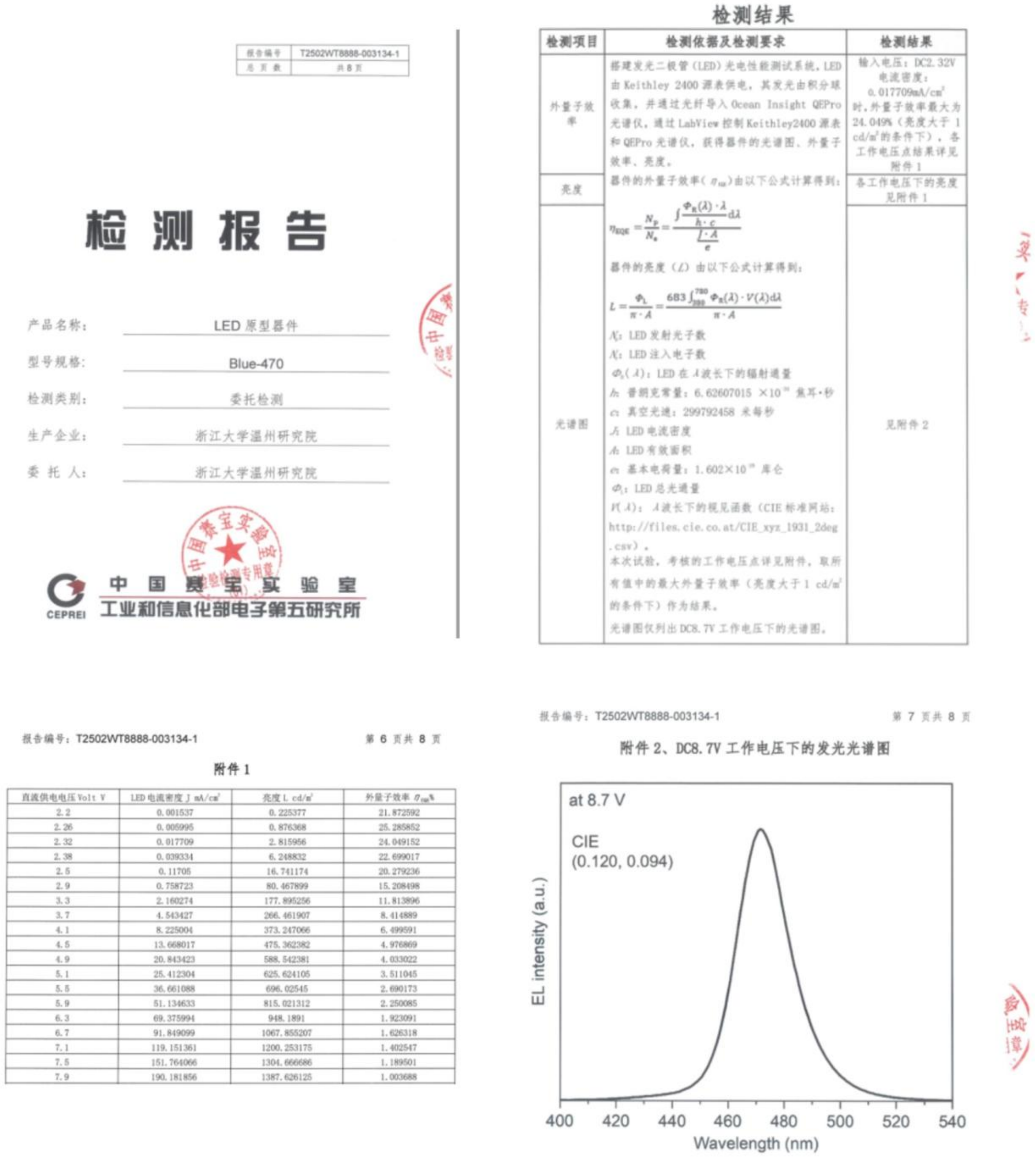

报告编号 T2502WT8888-003134-1
总页数 共 8 页

检 测 报 告

产品名称： LED 原型器件
型号规格： Blue-470
检测类别： 委托检测
生产企业： 浙江大学温州研究院
委 托 人： 浙江大学温州研究院

中国赛宝实验室
CEPREI 工业和信息化部电子第五研究所

检测结果

检测项目	检测依据及检测要求	检测结果
外量子效率	搭建发光二极管（LED）光电性能测试系统，LED 由 Keithley 2400 源表供电，其发光由积分球收集，并通过光纤导入 Ocean Insight QEPro 光谱仪，通过 LabView 控制 Keithley2400 源表和 QEPro 光谱仪，获得器件的光谱图、外量子效率、亮度。	输入电压：DC2.32V 电流密度：0.017709mA/cm² 时，外量子效率最大为 24.049%（亮度大于 1 cd/m² 的条件下），各工作电压点结果详见附件 1
亮度	器件的外量子效率（η_{EQE}）由以下公式计算得到：	各工作电压下的亮度见附件 1
光谱图	$\eta_{EQE}=\frac{N_p}{N_e}=\frac{\int\frac{\Phi_R(\lambda)\cdot\lambda}{h\cdot c}d\lambda}{\frac{J\cdot A}{e}}$ 器件的亮度（L）由以下公式计算得到： $L=\frac{\Phi_L}{\pi\cdot A}=\frac{683\int_{380}^{780}\Phi_R(\lambda)\cdot V(\lambda)d\lambda}{\pi\cdot A}$ N_p：LED 发射光子数 N_e：LED 注入电子数 $\Phi_R(\lambda)$：LED 在 λ 波长下的辐射通量 h：普朗克常量：6.62607015 ×10^[illegible] 焦耳·秒 c：真空光速：299792458 米每秒 J：LED 电流密度 A：LED 有效面积 e：基本电荷量：1.602×10^[illegible] 库仑 Φ_L：LED 总光通量 $V(\lambda)$：λ 波长下的视见函数（CIE 标准网站：http://files.cie.co.at/CIE_xyz_1931_2deg.csv）。 本次试验，考核的工作电压点详见附件，取所有值中的最大外量子效率（亮度大于 1 cd/m² 的条件下）作为结果。 光谱图仅列出 DC8.7V 工作电压下的光谱图。	见附件 2

报告编号：T2502WT8888-003134-1 第 6 页共 8 页

附件 1

直流供电电压 Volt V	LED 电流密度 J mA/cm²	亮度 L cd/m²	外量子效率 η_{EQE}%
2.2	0.001537	0.225377	21.872592
2.26	0.005995	0.876368	25.285852
2.32	0.017709	2.815956	24.049152
2.38	0.039334	6.248832	22.699017
2.5	0.11705	16.741174	20.279236
2.9	0.758723	80.467899	15.208498
3.3	2.160274	177.895256	11.813896
3.7	4.543427	266.461907	8.414889
4.1	8.225004	373.247066	6.499591
4.5	13.668017	475.362382	4.976869
4.9	20.843423	588.542381	4.033022
5.1	25.412304	625.624105	3.511045
5.5	36.661088	696.02545	2.690173
5.9	51.134633	815.021312	2.250085
6.3	69.375994	948.1891	1.923091
6.7	91.849099	1067.855207	1.626318
7.1	119.151361	1200.253175	1.402547
7.5	151.764066	1304.666686	1.189501
7.9	190.181856	1387.626125	1.003688

报告编号：T2502WT8888-003134-1 第 7 页共 8 页

附件 2、DC8.7V 工作电压下的发光光谱图

Figure S22. Certification by CEPREI (China Electronic Product Reliability and Environmental Testing Research Institute) with a certified maximum EQE of 24.0%. To clarify the validity of EQE data at low voltages (preventing accidental jumps or outliers), a segmented voltage application method was adopted for testing. Smaller voltage steps (0.06 V) were used at lower voltages, while larger voltage steps (0.4 V) were applied at higher voltages.

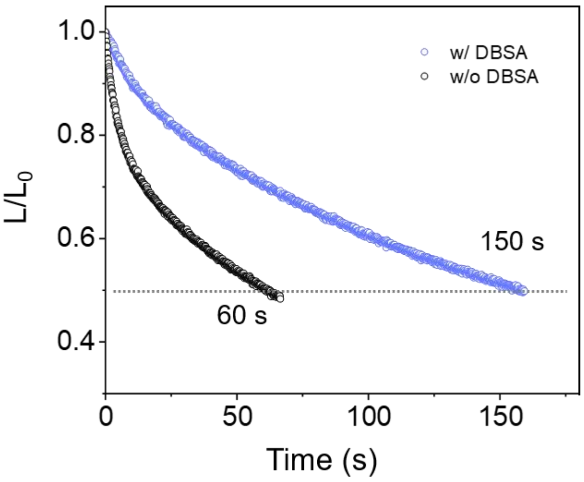

Figure S23. Normalized luminance as a function of working time for LEDs at an initial luminance of 100 cd m^{-2} with and without the DBSA group.

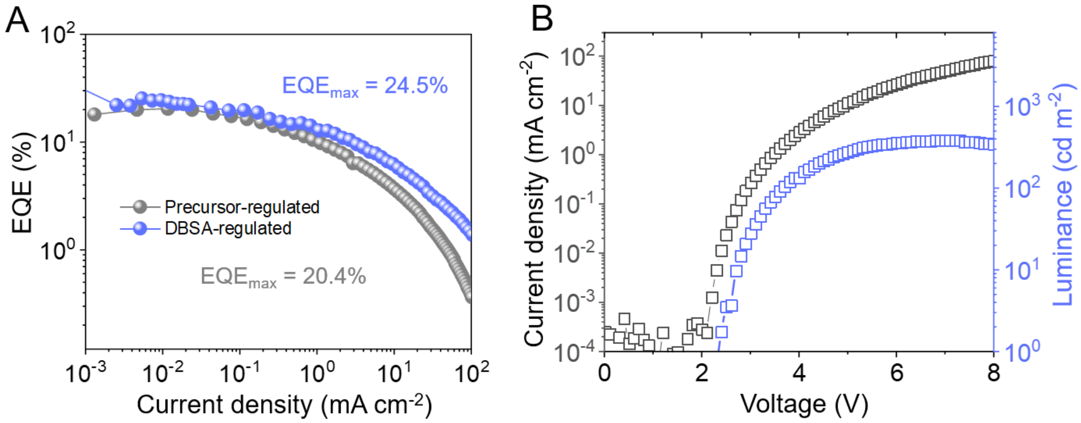

Figure S24 (A) EQE-current density curves of the LEDs based on nanocrystals with Cl/Br ratio regulated by precursor or DBSA. (B) Typical current density–voltage-luminance curves of the LED based on nanocrystals with a Cl/Br ratio regulated by precursor.

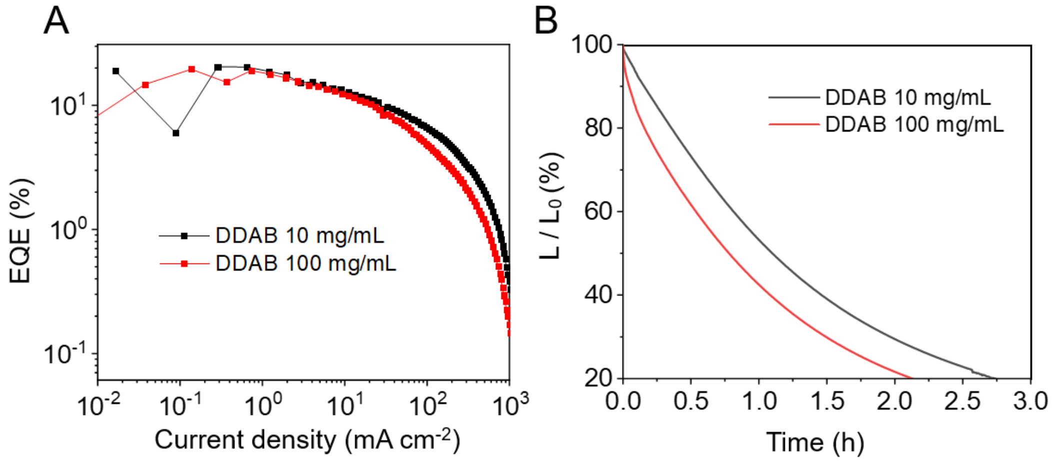

Figure S25 (A) EQE-current density curves of the LEDs based on pure-Br nanocrystals passivated by different dosages of DDAB and (B) normalized luminance as a function of working time for LEDs at an initial luminance of 100 cd m^{-2}.

Table S1. The precise halogen content in nanocrystals synthesized with different DBSA dosages measured by Ion chromatography.

Nanocrystals	Cl^- (mg mL^{-1})	Cl^- (mmol mL^{-1})	Br^- (mg mL^{-1})	Br^- (mmol mL^{-1})	Cl: Br (molar ratio)
Pristine	0.5028	0.01418	0.9773	0.01223	1.159
DBSA 0.2	0.4110	0.01159	1.3158	0.01647	0.7037
DBSA 0.4	0.2964	0.00836	1.4079	0.01762	0.4745
DBSA 0.6	0.2579	0.00728	1.4933	0.01869	0.3895
DBSA 0.8	0.2166	0.00611	1.5374	0.01924	0.3176
DBSA 1.0	0.2072	0.00584	1.7481	0.02188	0.2669

Table S2. Synthesis conditions of nanocrystals with or without DBSA at an emission wavelength of 470 nm (dissolved in 5 mL of toluene, with 0.5 mL of Cs-PA triggering the reaction).

**Nanocrystals**	**$PbBr_2$**	**$PbCl_2$**	**TOAB**	**DBSA (1 g mL^{-1})**	**DDAC (0.1 g mL^{-1})**	**DDAB (0.1 g mL^{-1})**
w/ DBSA	0.4 mmol	0.1 mmol	0.725 mL	0.6 mL	1.2 mL	0 mL
w/o DBSA	0.4 mmol	0.1 mmol	0.725 mL	0 mL	0.2 mL	1.0 mL

REFERENCES

1. Jiang Y., Cui M., Li S., et al. (2021). Reducing the impact of Auger recombination in quasi-2D perovskite light-emitting diodes. *Nat. Commun.* **12**:336. DOI:10.1038/s41467-020-20555-9

2. Jiang Y., Sun C., Xu J., et al. (2022). Synthesis-on-substrate of quantum dot solids. *Nature* **612**:679-684. DOI:10.1038/s41586-022-05486-3

3. Hu J., Li J., Lu G., et al. (2024). Monoammonium Modified Dion-Jacobson Quasi-2D Perovskite for High Efficiency Pure-Blue Light Emitting Diodes. *Small* **20**:2402786. DOI:10.1002/smll.202402786

4. Karlsson M., Yi Z., Reichert S., et al. (2021). Mixed halide perovskites for spectrally stable and high-efficiency blue light-emitting diodes. *Nat. Commun.* **12**:361. DOI:10.1038/s41467-020-20582-6

5. Liu Y., Wang S., Yu Z., et al. (2023). A Multifunctional Additive Strategy Enables Efficient Pure-Blue Perovskite Light-Emitting Diodes. *Adv. Mater.* **35**:2302161. DOI:10.1002/adma.202302161

6. Bi C., Yao Z., Hu J., et al. (2023). Suppressing Auger Recombination of Perovskite Quantum Dots for Efficient Pure-Blue-Light-Emitting Diodes. *ACS Energy Lett.* **8**:731-739. DOI:10.1021/acsenergylett.2c02613

7. Wei S., Hu J., Bi C., et al. (2024). Strongly-Confined CsPbBr3 Perovskite Quantum Dots with Ultralow Trap Density and Narrow Size Distribution for Efficient Pure-Blue Light-Emitting Diodes. *Small* **20**:2400885. DOI:10.1002/smll.202400885

8. Tong Y., Bi X., Xu S., et al. (2023). In Situ Halide Exchange of Cesium Lead Halide Perovskites for Blue Light-Emitting Diodes. *Adv. Mater.* **35**:2207111. DOI:10.1002/adma.202207111

9. Chen F., Liu Y., Zhang D., et al. (2023). Bilayer phosphine oxide modification toward efficient and large-area pure-blue perovskite quantum dot light-emitting diodes. *Sci. Bull.* **68**:2354-2361. DOI:10.1016/j.scib.2023.09.014

10. Jiang M., Zhang X. and Wang F. (2024). Efficient Perovskite Nanograin Light-Emitting Diodes in Green-to-Blue Gamut with Co-Additive Engineering. *Adv. Mater.* **36**:2400565. DOI:10.1002/adma.202400565

11. Gao L., Zhang Y., Gou L., et al. (2022). High efficiency pure blue perovskite quantum dot light-emitting diodes based on formamidinium manipulating carrier dynamics and electron state filling. *Light-Sci. Appl.* **11**:346. DOI:10.1038/s41377-022-00992-5

12. Yuan S., Dai L., Sun Y., et al. (2024). Efficient blue electroluminescence from reduced-dimensional perovskites. *Nat. Photon.* **18**:425-431. DOI:10.1038/s41566-024-01382-6

13. Zhang L., Jiang Y., Feng Y., et al. (2023). Manipulating Local Lattice Distortion for Spectrally Stable and Efficient Mixed-halide Blue Perovskite LEDs. *Angew. Chem. Int. Ed.* **62**:e202302184. DOI:10.1002/anie.202302184

14. Yang Y., Xu S., Ni Z., et al. (2021). Highly Efficient Pure-Blue Light-Emitting Diodes Based on Rubidium and Chlorine Alloyed Metal Halide Perovskite. *Adv. Mater.* **33**:2100783. DOI:10.1002/adma.202100783

15. Ma L., Li X., Li X., et al. (2024). Eliminating Chlorine Vacancies of Perovskite Nanocrystals Using Hydrazine Cations Enables Efficient Pure Blue Light-Emitting Diodes. *ACS Energy Lett.* **9**:1210-1218. DOI:10.1021/acsenergylett.4c00109

16. Gao Y., Cai Q., He Y., et al. (2024). Highly efficient blue light-emitting diodes based on mixed-halide perovskites with reduced chlorine defects. *Sci. Adv.* **10**:eado5645. DOI:10.1126/sciadv.ado5645
