# Supplementary material for: Surface-ligand-triggered synthetic control of defects in nanocrystals toward high-efficiency blue electroluminescence
Source: Innovation (Camb). 2026 Jan 20;7(6):101273. doi: 10.1016/j.xinn.2026.101273 (PMC13237842; doi:10.1016/j.xinn.2026.101273)
Supplement: Document S2. Article plus supplemental information [file mmc2.pdf]

REPORT Materials Chemistry

OPEN ACCESS

Surface-ligand-triggered synthetic control of defects in nanocrystals toward high-efficiency blue electroluminescence

Qingli Cao,[1] Qiuting Cai,[1] Yifeng Feng,[1] Xinyang Wang,[1] Dingshuo Zhang,[1] Yun Gao,[1] Haoran Zhang,[1] Meiyi Zhu,[2] Yifan He,[3] Haiping He,[1,2] Zhizhen Ye,[1,2] and Xingliang Dai[1,2,*]
*Correspondence: shanfeng@zju.edu.cn

Received: May 28, 2025; Accepted: January 16, 2026; Published Online: January 20, 2026; https://doi.org/10.1016/j.xinn.2026.101273

© 2026 The Author(s). Published by Elsevier Inc. on behalf of Youth Innovation Co., Ltd. This is an open access article under the CC BY license (http://creativecommons.org/licenses/by/4.0/).

GRAPHICAL ABSTRACT

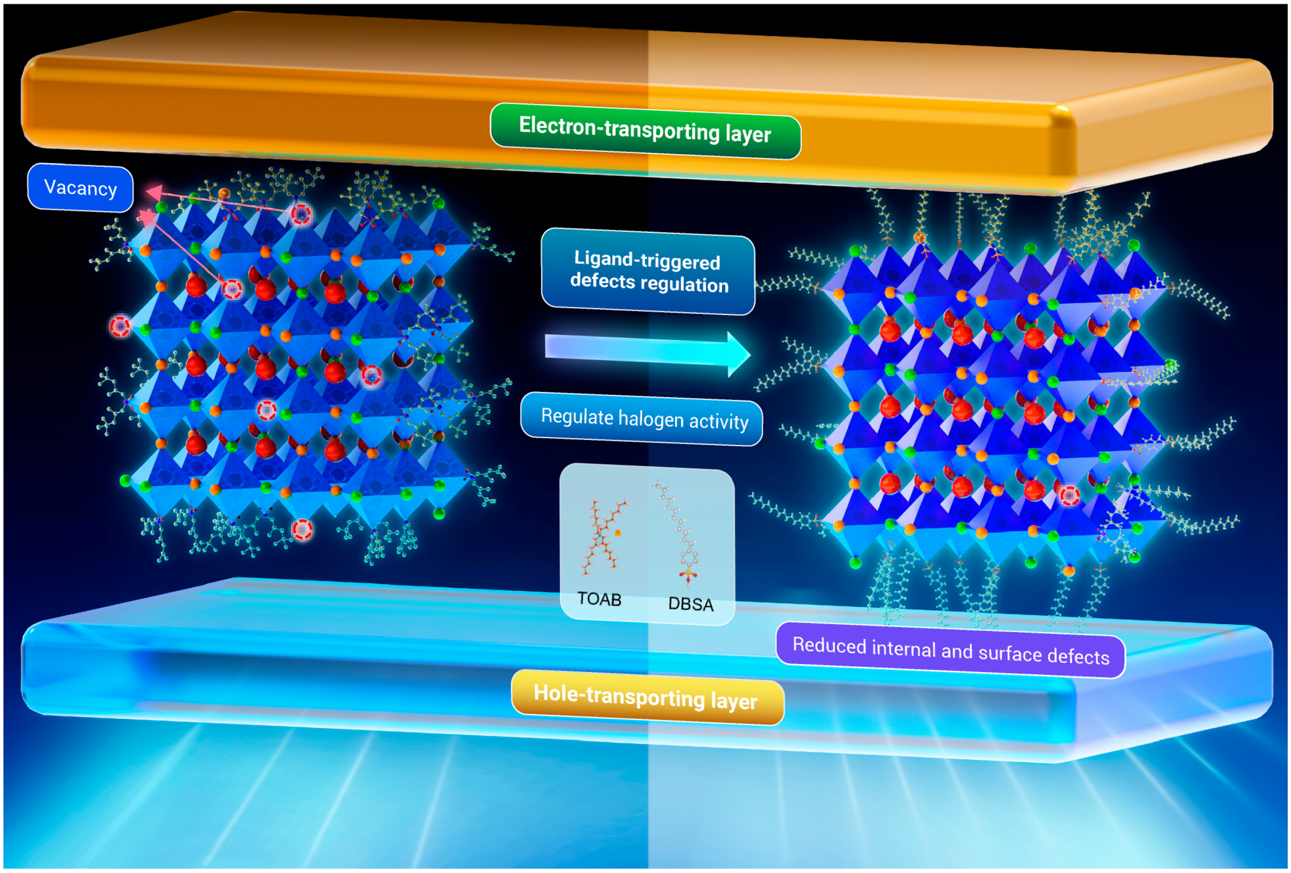

PUBLIC SUMMARY

- Strong acid ligands modulate Cl defects, generalizing ligand-triggered synthetic control of holistic defects.
- Pure-blue perovskite LEDs emitting at 470 nm achieve a record external quantum efficiency of 24.5%.
- Mechanism studies of ligand-driven defect suppression advance the rational design of colloidal nanocrystals.

Surface-ligand-triggered synthetic control of defects in nanocrystals toward high-efficiency blue electroluminescence

Qingli Cao,[1] Qiuting Cai,[1] Yifeng Feng,[1] Xinyang Wang,[1] Dingshuo Zhang,[1] Yun Gao,[1] Haoran Zhang,[1] Meiyi Zhu,[2] Yifan He,[3] Haiping He,[1,2] Zhizhen Ye,[1,2] and Xingliang Dai[1,2,*]

[1]School of Materials Science and Engineering, State Key Laboratory of Silicon and Advanced Semiconductor Materials, Zhejiang University, Hangzhou 310027, China
[2]Wenzhou Key Laboratory of Novel Optoelectronic and Nano Materials, Engineering Research Centre of Zhejiang Province, Institute of Wenzhou, Zhejiang University, Wenzhou 325006, China
[3]Wenzhou XINXINTAIJING Tech. Co., Ltd., Wenzhou 325006, China
*Correspondence: shanfeng@zju.edu.cn

Received: May 28, 2025; Accepted: January 16, 2026; Published Online: January 20, 2026; https://doi.org/10.1016/j.xinn.2026.101273
© 2026 The Author(s). Published by Elsevier Inc. on behalf of Youth Innovation Co., Ltd. This is an open access article under the CC BY license (http://creativecommons.org/licenses/by/4.0/).
Citation: Cao Q., Cai Q., Feng Y., et al., (2026). Surface-ligand-triggered synthetic control of defects in nanocrystals toward high-efficiency blue electroluminescence. The Innovation **7(6)**, 101273.

Colloidal nanocrystals have emerged as promising building blocks for optoelectronic applications. Although substantial research has focused on the roles of surface ligands in controlling the crystal structure, morphology, dispersibility, and surface properties of nanocrystals, limited attention has been devoted to ligand-triggered internal defect regulation. Here, we exemplify that the defects of blue-emitting $CsPb(Br_xCl_{1-x})_3$ nanocrystals can be extensively regulated with surface ligands, enabling outstanding optical and electroluminescence performance. The ionization reaction of the strong dodecylbenzenesulfonic acid (DBSA) ligand, combined with its interactions with the precursors, modulates the concentration of halide ions in the reactant and controls activity. The increased DBSA results in a significantly reduced chlorine content in the nanocrystals and thus suppresses the formation of internal chlorine-related defects, evidenced by thermal admittance spectroscopy and optical characterization. Immediate application of this understanding allows defect-less $CsPb(Br_xCl_{1-x})_3$ nanocrystals with efficient radiative recombination to be synthesized by controlling the DBSA dosage, realizing blue light-emitting diodes with an external quantum efficiency of 24.5% and a brightness of over 1,000 cd m^{-2} at 470 nm. This work provides new insights into the underlying mechanisms governing how ligands influence nanocrystal properties beyond those well-known functions, advancing nanocrystal synthesis from empirical exploration to rational design paradigms.

INTRODUCTION

The intriguing features induced by nanosurface and size effects have facilitated the unprecedented rapid development of colloidal nanocrystals in next-generation optoelectronic devices, biomedicine, and quantum computation.[1–3] During the past decade, perovskite nanocrystals emerged as a frontier in electroluminescence due to their narrow emissions and feasible color modulation.[4–6] Efficient blue electroluminescence becomes the most critical challenge toward further advancement for applications such as full-color displays.[7–9] Blue emission could be achieved through strong confinement from the reduced dimensions of the $CsPbBr_3$ nanocrystal. However, the synthesis of ultra-small nanocrystals or ultra-thin nanoplates induces great challenges in ensuring size uniformity, accompanied by the delicate control of surface ligands, a trade-off for colloidal stability and carrier transport.[10–14] Another approach to realizing blue emission involves introducing chlorine via halogen doping, which allows continuous spectrum adjustment across the green to the deep-blue region.[15,16] In this regard, the composition can feasibly adjust the emission, providing great synthesis versatility for perovskite nanocrystals with targeted wavelengths. Nonetheless, mixed halogen perovskite nanocrystals suffer from low photoluminescence quantum yield (PLQY) induced by deep-level chlorine defects.[9,17] Controlling the chlorine content and reducing chlorine-related internal defects is key to improving the luminescence efficiency of $CsPb(Br_xCl_{1-x})_3$ nanocrystals.[18]

During the synthesis, two factors synergistically dominate the properties of colloidal nanocrystals, namely precursors (e.g., lead halide and cesium halide for perovskite nanocrystals) and ligands (e.g., oleylamine and oleic acid [OA]), which evolve into the inorganic core and the organic ligand shell, respectively.[19,20] Specifically, the concentration and activity of precursors affect the composition and inherent properties of the inorganic core, while organic ligands modulate the crystal structure, morphology, dispersibility, and surface properties.[21–24] For example, in mixed-halide blue-emission perovskite systems, precursor components and ion activity are key factors that affect the band gap of nanocrystals, usually altered by controlling the ratio of chloride to bromide in the precursor, while ligands are employed in morphology regulation and surface defect passivation.[25–27] In this context, the influence of ligands on the inorganic core of nanocrystals is customarily overlooked; thus, the impact and controllability of ligands on components and defects remain an enigmatic issue and call for intense investigations.

Here, we systematically unraveled the acid-ligand-triggered component regulation and internal defect suppression in synthesizing mixed-halide perovskite nanocrystals. The nontrivial involvement of dodecylbenzenesulfonic acid (DBSA) in controlling the chlorine-bromine ratio of nanocrystals was authenticated, even though it does not contain inorganic perovskite components. This provides an opportunity to suppress internal halogen vacancies in the nanocrystals, whose defects are highly related to the halogen content, and regulate the optical emission by varying the dosage of DBSA. Following this perspective, $CsPb(Br_xCl_{1-x})_3$ nanocrystals with different amounts of DBSA were synthesized, and their optical properties and electroluminescence performance were investigated. Using this typical acid ligand, we elucidate the chemical mechanisms by which ligand design modulates halogen activity and suppresses intrinsic defects in nanocrystals, highlighting the superiority of ligand regulation in controlling the surface and internal defects of nanocrystals.

MATERIALS AND METHODS

Materials

Cesium carbonate (Cs_2CO_3, 99.9%), $PbBr_2$ (99.999%), $PbCl_2$ (99.999%), and DBSA (≥95%) were purchased from Sigma-Aldrich. Rubidium carbonate (Rb_2CO_3, 99.9%), formamidine acetate (FA(Ac), 99%), bis(2,4,4-trimethylpentyl)phosphinic acid (PA; 90%), didodecyldimethylammonium chloride (DDAC; 98%), didodecyldimethylammonium bromide (DDAB; 98%), tetra-*n*-octylammonium bromide (TOAB; 98%), ethyltributyl phosphonium bromide (ETBPB; ≥99%), ethyl acetate (EA; 99%), nickel acetate tetrahydrate ($NiAc_2·4H_2O$, 99.9%), ethanolamine (≥99.0%), Nafion perfluorinated resin (PFI) solution (5 wt % in a mixture of lower aliphatic alcohols and water, contains 45% water), and LiF (99.99%) were purchased from Shanghai Macklin Biochemical. 1,3,5-tris(1-phenyl-1H-benzimidazol-2-yl) benzene (TPBi), PEDOT:PSS, and PO-T2T were purchased from Xi'an Yuri Solar Co. Ltd. Oleic acid (OA, 90%, Alfa Aesar), n-Octanoic acid (OTAc, 99%, Aladdin), ultradry toluene (≥99.5%, Chengdu Chron Chemical), ultradry octane (97%, J&K Scientific), and PF8Cz (Dongguan Volt-Amp Optoelectronics Technology) were purchased from the respective suppliers and all chemicals were used directly without further purification.

Synthesis of nanocrystals

PbX_2 stock solution was prepared by dissolving $PbBr_2$ (4 mmol), $PbCl_2$ (1 mmol), and TOAB (7.25 mmol) in toluene (50 mL) at room temperature and filtered through a 0.22 μm filter after complete dissolution. The Cs/FA/Rb-PA solution was prepared by mixing Cs_2CO_3 (0.85 mmol), Rb_2CO_3 (0.085 mmol), and FAAc (0.15 mmol) with PA (10 mL) at 80°C. Nanocrystals were synthesized in a nitrogen glove box, taking 5.3 mL PbX_2 precursor in a 20 mL vial, and x mL (x = 0, 0.2, 0.4, 0.6, 0.8, or 1.0) DBSA (1 g mL^{-1} in toluene) was added under vigorous stirring. After 1 min of stirring, Cs/FA/Rb-PA precursor (0.5 mL) was injected swiftly. DDAC solution (1 mL, 0.1 g mL^{-1} in toluene) was added after reacting for 2 min and kept under stirring for another 5 min.

The nanocrystals with emission at 470 nm, synthesized with or without DBSA, were obtained by adjusting the amount of DDA^+. To achieve a similar surface passivation effect, the

nanocrystals with emission at 470 nm synthesized without DBSA were treated with a mixture of DDAB and DDAC (see Table S2 for details). To create the pure bromine nanocrystals, all chlorides were replaced by bromides: $PbCl_2$ was substituted by $PbBr_2$ and DDAB (10 mg mL^{-1}) was used in place of DDAC.

The purification process was carried out under air conditions. EA was added to the crude solution at a volume ratio of 2:1 (this value gradually changes from 2:1 to 4:1 as DBSA increases, due to the reduced size of nanocrystals needing more anti-solvents for precipitation), and then centrifuged at 8,000 rpm for 3 min. The precipitate was collected and dispersed in octane. The purification process was repeated two times for device fabrication and characterization. To ensure precise controllability over the emission wavelength, the temperature was maintained at ~25°C throughout the synthesis process.

LED fabrication

The indium tin oxide (ITO) substrate was sequentially ultrasonicated in acetone (20 min), deionized water (15 min), and ethanol (15 min), followed by treatment with oxygen plasma for 15 min. Under ambient conditions (~50% relative humidity [RH]), 50 μL NiO_x precursor (0.06 M $NiAc_2·4H_2O$ and 0.06 M ethanolamine dissolved in ethanol) was spin coated on the substrate at 5,000 rpm for 45 s and annealed at 280°C for 30 min. PEDOT:PSS solution premixed with PFI (1:1 volume ratio) was then spun onto the NiO_x layer at 10,000 rpm for 15 s, followed by annealing at 150°C for 15 min. The substrates were transferred into a glove box with N_2 conditions for further fabrication. PF8Cz (8 mg mL^{-1} in chlorobenzene) was spin coated at 3,000 rpm for 35 s and annealed at 150°C for 30 min. The nanocrystal solution was diluted with n-octane, the additive ETBPB (7.5 mM in toluene) was added (10:1 volume ratio) to further passivate surface defects, and the mixture was filtered after being mixed evenly. Subsequently, the blue perovskite nanocrystals were spin coated at 2,000 rpm for 50 s. Later, under a vacuum below 4×10^{-4} Pa, TPBi (5 nm, 0.3 Å s^{-1}), PO-T2T (45 nm, 0.3 Å s^{-1}), and LiF/Al electrodes (1/80 nm, 0.1/1.0 Å s^{-1}) were deposited sequentially in a thermal evaporator.

Optical characterizations

The UV-visible (UV-vis) absorption spectra of the nanocrystals were measured using an Agilent Cary 5000. The PL spectra and time-resolved fluorescence spectra were performed using OmniFluo990 with an excitation source of a xenon flash lamp and a 405-nm pulsed laser diode (EPL405, 58.6-ps pulse width), respectively. The absolute PLQYs were measured using a xenon lamp that was filtered to 405 nm as excitation wavelength and a home-designed integrating sphere coupled to a QE Pro spectrometer.[28,29] The temperature-dependent PL measurements were conducted with a Zolix Instruments OmniFluo900 spectrometer equipped with a picosecond pulsed laser diode (405 nm, 58.6 ps) and a liquid nitrogen cooling instrument.

Transient absorption (TA) spectra were measured utilizing a femtosecond pump-probe spectroscopy setup. The output light from a light Conversion Pharos Yb:KGW laser (1,030 nm, 200 fs, 200 μJ per pulse, and a 100 kHz repetition rate) was split into two beams: one passed through an OptoSigma OSMS26-300 optical delay line and then focused onto a barium borate oxide (BBO) crystal to generate a probe beam and the other was introduced to a Light Conversion Orpheus-HP optical parameter amplifier to generate a pump beam at a desired wavelength of 405 nm, passed through a round continuously variable metallic neutral density filter and a chopper working at 40 kHz. During the measurements, the perovskite nanocrystal solution was irradiated by the pump beam. The optical power density of the pump light was attenuated to 3.36 μJ cm^{-2}.

The picosecond time-resolved photoluminescence (TRPL) dynamics were obtained through the low-frequency mode of a streak camera (ST-10, Zolix). A femtosecond laser was used as the excitation source with a repetition rate of 1 kHz and an excitation wavelength of 405 nm. The excitation light intensity was 0.35 μJ cm^{-2} focused on the sample. The perovskite nanocrystal solutions were filled into quartz cuvettes with a 1-mm light path, and the sample solutions were under continuous stirring during TA and TRPL measurements.

Defect-state characterizations

The space-charge-limited current (SCLC) measurement was based on the hole-only device with a multilayer structure of ITO/NiO_x/PEDOT:PSS:PFI/PF8Cz/nanocrystals/MoO_3/Al. The J-V curves of devices were collected in a forward scan (0–3 V, step: 0.01 V) using a Keithley 2400 in the dark. For the thermal admittance spectroscopy (TAS) measurement, a Zurich Instruments MFIA5M was utilized. The capacitance (C) was measured by applying an AC voltage with an amplitude of V_{ac} = 100 mV and varying the angular frequency (ω) from 10 Hz to 5 MHz. The E_a of the defects and the energetic profile of the trap density of states (tDOS) were calculated using the following equations[30]:

$$\omega_T = 2\pi f e^{-\frac{E_a}{kT}}, \quad \text{(Equation 1)}$$

$$E_\omega = kT\ln\frac{2\pi f}{\omega}, \text{ and} \quad \text{(Equation 2)}$$

$$N_T(E_\omega) = -\frac{V_{bi}}{de}\frac{dC}{d\omega}\frac{\omega}{kT}, \quad \text{(Equation 3)}$$

where f is the attempt-escape frequency, k is the Boltzmann constant, V_{bi} is the built-in potential, and d is the width of the depletion layer.

Composition and crystal structure characterizations

Transmission electron microscopy (TEM) observations were conducted using a Hitachi HT-7700 microscope operated at 80 kV. Spherical aberration-corrected high-angle annular dark-field STEM (HAADF-STEM) observations were conducted using an FEI Titan G2 80-200 ChemiSTEM spherical aberration-corrected TEM operated at 200 kV. X-ray photoelectron spectroscopy (XPS) spectra were obtained on a ThermoFisher ESCALAB Xi+ equipment in an ultrahigh vacuum chamber with a vacuum of 8×10^{-10} Torr and Al Kα source. Crystal structures were analyzed by X-ray diffraction (XRD; Rigaku D/MAX 2500) operated at 40 keV and 40 mA with Cu Kα radiation (λ = 1.5406 Å). The actual contents of halogens were detected using ion chromatography (AQUION, DIONEX). The perovskite nanocrystal solution was dried with nitrogen, and the obtained solid was dissolved 1,000 times in deionized water for ion chromatography measurements.

Device characterizations

The current density-luminance-voltage curves, external quantum efficiencies (EQEs), EL spectra, and Commission Internationale de l'Éclairage (CIE) coordinate characterizations of light-emitting diodes (LEDs) were measured with a Keithley 2400 electrometer and an integration sphere coupled with a QE Pro spectrometer.[31] The EQE (η_{EQE}) of the device is calculated using the following formula:

$$\eta_{EQE} = \frac{N_p}{N_e} = \frac{\int \frac{\Phi_R(\lambda)\cdot\lambda}{h\cdot c}d\lambda}{\frac{J\cdot A}{e}}.$$

The luminance (L) of the device is calculated using the following formula:

$$L = \frac{\Phi_L}{\pi\cdot A} = \frac{683\int_{380}^{780}\Phi_R(\lambda)\cdot V(\lambda)d\lambda}{\pi\cdot A},$$

where N_p is the number of photons emitted by the LED, N_e is the number of injected electrons, $\Phi_R(\lambda)$ is the radiant flux at wavelength λ, h is Planck's constant, c is the speed of light in vacuum, J is the current density, A is the effective area of the device, e is the elementary charge, Φ_L is the total luminous flux, and $V(\lambda)$ is the photopic vision function at wavelength λ.

The devices were swept from zero bias to forward bias, and a segmented voltage application method was adopted to accurately measure the efficiency at low brightness. The swept speed was 0.03/0.1/0.2 V per 100 ms with an additional integration time of spectral acquisition of 50 ms. The operational stability was conducted by applying a constant current density to the devices, and the EL spectra were recorded. The performance of the devices was measured in the glove box filled with nitrogen at room temperature.

RESULTS AND DISCUSSION

Activity modulation of halogens with strong organic acid ligands

Mixed-halide $CsPb(Br_xCl_{1-x})_3$ nanocrystals were synthesized using a modified ligand-assisted reprecipitation method (see materials and methods for details),[32,33] as shown in Figure 1A. The nucleation process of the nanocrystals is illustrated in Figure 1B, where the existing form of lead halides is represented as octahedra for simplicity. In this methodology, TOAB forms a complex with polar lead halide to enhance the solubility of halide in low-polarity solvent toluene (equation 1 in Figure 1B). The optimized PA was utilized for dissolving the cesium source, and the injection of cesium phosphate triggered the crystallization of $CsPb(Br_xCl_{1-x})_3$ nanocrystals. DDAC post-treatment effectively passivates surface defects and modulates the emission wavelength of nanocrystals (Figure S1A).[34] Notably, while the DDAC ligand does not inherently introduce Cl defects, an excess of DDAC increases the overall chloride content in the

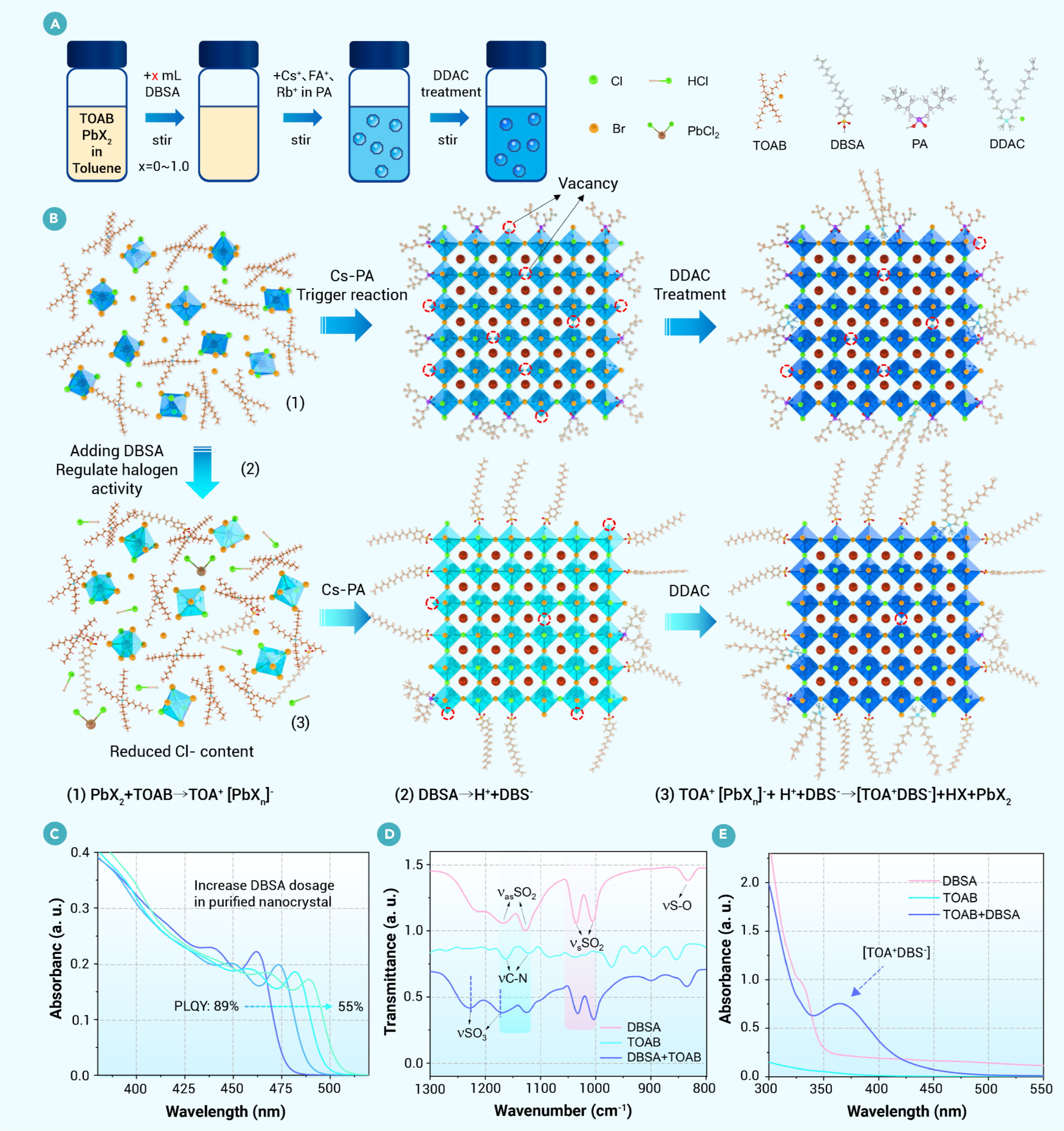

Figure 1. Activity modulation of halogens with strong organic acid ligands in the synthesis of nanocrystals (A) Schematic representation of nanocrystal synthesis and post-treatment processes. (B) Schematic illustration of the nanocrystal synthesis procedures and DBSA-mediated activity regulation of halide ions (some hydrogen atoms are omitted for the sake of simplicity), showing the formation of HCl and the precipitation of $PbCl_2$ after adding DBSA to the precursor solution. (C) Absorption spectra transformation of the purified $CsPb(Br_xCl_{1-x})_3$ nanocrystals (470 nm, with DBSA) after adding an increasing amount of DBSA (DBSA added to nanocrystal octane solution). (D) Fourier transform infrared spectra of DBSA, TOAB, and their equimolar mixtures. (E) Absorption spectra of DBSA, TOAB, and their equal mixtures dissolved in toluene.

system, thereby making the generation of Cl defects more probable. A preliminary investigation revealed that factors such as the acid ligand species led to significant differences in the emission wavelength of nanocrystals, even under identical feed halogen ratios (Figure S1B). This prompted us to investigate the impact of acid ligands on halogen activity in the system to unveil the underlying mechanisms.

It is well established that the synthesis of perovskite nanocrystals is typically conducted in low-polarity organic solvents, such as 1-octadecene, octane, or toluene, to minimize structural damage. In this reactant, incomplete ionization of HX (X = Br or Cl) makes it not behave as a strong acid, as evidenced by its significantly lower acid dissociation constant (larger pKa) in organic solvents (e.g., toluene) than in aqueous solutions.[35,36] The introduction of strong organic

acids in the reactant generates numerous hydrogen ions, which could combine with halide ions to form low-reactive hydrogen halides, resulting in undesirable halogen content within the nanocrystals. In this sense, we may regulate nanocrystal emission by adjusting the halogen activity through organic acids.

DBSA has been employed in perovskite nanocrystal synthesis, particularly in room temperature systems, due to its superior surface coordination capability that outperforms traditional OA.[37–39] Additional reports have highlighted that DBSA effectively suppresses phase segregation in mixed-halide nanocrystals.[40] Furthermore, post-synthetic ion-pair engineering based on benzenesulfonate derivatives has been shown to regulate halide distribution and stability within these systems.[36] Those studies predominantly focus on surface state modification or macroscopic crystallization effects and lack systematic theoretical interpretation of the intrinsic driving forces behind compositional and crystallographic changes. The DBSA ligand possesses strong acidity and undergoes partial ionization into H^+ and DBS^- (equation 2 in Figure 1B) in the solvents for synthesizing nanocrystals, which are capable of adjusting halogen activity. After adding DBSA to the reactant, the pH value of the solution decreases from 6.0 to 1.0 (Figure S2), authenticating the ionization process of DBSA in toluene. The combination of the generated hydrogen ions with halogens could reverse the ionization reaction, forming the less reactive HX. Due to the large electronegativity of chlorine (3.16) compared with bromine (2.96), chloride ions are inclined to deplete from the organic solution to form nonionized HCl.[41] This would substantially decrease the chlorine-to-bromine ratio in the nanocrystals and change the halogen activity within the reactant. To validate the hypothesis that the chloride ions are preferred to combine with the hydrogen ions, we introduced DBSA in the purified $CsPb(Br_xCl_{1-x})_3$ nanocrystal reactant. The absorption spectra are progressively redshifted, and the absorbance is gradually reduced upon increasing the amount of DBSA (Figure 1C), inferring the reduced chlorine component in the nanocrystals. This process generates massive chlorine vacancies, resulting in a reduction in PLQY. Introducing another strong organic trifluoromethanesulfonic acid (TFMSA) also shows analogous behavior in the $CsPb(Br_xCl_{1-x})_3$ nanocrystal reactant, while for pure $CsPbBr_3$ nanocrystals, non-distinguishable spectral shifts are observed (Figure S3). This indicates that strong organic acids exert similar effects on halogen activity in mixed-halide systems; they can modulate halogen activity via their acidity, which in turn regulates nanocrystal composition. However, since TFMSA is not a competent ligand, the corresponding nanocrystals exhibit inferior optical properties, with the PLQY of those emitting at 470 nm only reaching 48%. Collectively, these findings highlight that strong organic acids modulate the composition of nanocrystals beyond their conventional functionality.

Besides the ionization reaction of the DBSA, the interactions between DBSA and other precursors were investigated through Fourier transform infrared (FTIR) spectroscopy and absorption spectra. As shown in Figure 1D, FTIR spectroscopy reveals the stretching vibration peak (833 cm^{-1}) of the S–O bond, the symmetric stretching vibration peaks (1,005 and 1,035 cm^{-1}), and antisymmetric stretching vibration peaks (1,127 and 1,166 cm^{-1}) of the S=O bond in DBSA,[37] as well as the symmetric stretching vibration peaks (1,121 and 1,163 cm^{-1}) of the C–N bond in TOAB. After mixing DBSA and TOAB, the stretching vibrations of the S=O and S–O bonds weakened. In contrast, the symmetric stretching vibration (1,228 cm^{-1}) and antisymmetric stretching vibration (1,170 cm^{-1}) of SO_3 intensified, suggesting that the combination of DBSA and TOAB generates sulfonic acid groups. Furthermore, the absorption spectrum displays a new strong absorption peak following the mixing of DBSA with TOAB (Figure 1E). These results suggest that DBSA reacts with the quaternary ammonium salt to form a complex, which disturbs the equilibrium between TOA^+ and PbX_n^- and results in the precipitation of lead halides. As a verification, we introduced an adequate amount of DBSA into the precursor solution ($PbBr_2$, $PbCl_2$, and TOAB in toluene), causing the system to become turbid and precipitate. Ion chromatography characterizations were performed on the original precursor solution, supernatant, and precipitate, revealing the highest chlorine content in the precipitate (Figure S4). This verifies that the introduction of DBSA mainly precipitates $PbCl_2$, originating from its pronounced molecular polarity.[36] The precipitate could be redissolved in toluene upon the addition of TOAB. The conclusion underscores that the strongly organic acid ligand additives can regulate the ion concentration of bromide and chloride in the reactant during the synthesis of nanocrystals, enabling the activity modulation of halogens (equation 3 in Figure 1B).

Size and composition regulation of nanocrystals

Aiming to reveal the effects of DBSA on managing halogen activity within the system, a series of nanocrystals was synthesized under controlled conditions, only varying the amount of DBSA additive in the precursor solution. Pristine, DBSA 0.2, DBSA 0.4, DBSA 0.6, DBSA 0.8, and DBSA 1.0 refer to the nanocrystals synthesized without DBSA and with 0.2, 0.4, 0.6, 0.8, and 1.0 mL DBSA-toluene mixture (1 g mL^{-1} for DBSA), respectively. As shown in Figure 2A, the average size of the nanocrystals decreases with the increased amount of DBSA, ranging from approximately 10 to 6 nm on average (Figure 2B). This observation is consistent with the thermodynamic theory for the nucleation of colloidal particles. In this theory, the minimum size of nuclei that are stable to dissolution, known as the critical radius (r_c), is given by[23,42]

$$r_c = \frac{2\gamma V_m}{N_A k_B T \ln S},$$

where N_A is Avogadro's number, k_B is the Boltzmann constant, V_m is the molar volume, and T is the temperature. The size of the formed nuclei is governed by the supersaturation (S) of precursor components and the surface energy (γ). The ultimate nanoparticle size is governed by the interplay between nucleation (controlled by r_c) and subsequent growth kinetics (influenced by precursor diffusion and concentration). Specifically, a smaller r_c (high S or low γ) accelerates nucleation, generating numerous nuclei that exhaust precursors quickly, leading to limited growth and smaller final nanoparticle sizes. Conversely, a larger r_c (low S or high γ) reduces nucleation density, allowing abundant precursors to sustain the prolonged growth of individual nuclei, ultimately yielding larger nanoparticles. In this case, an increase in DBSA, which functions as a strong surface-binding ligand, reduces the surface energy and consequently leads to the formation of smaller nanocrystals. From a supersaturation perspective, the interaction between DBS^- and TOA^+ elevates the supersaturation level of lead halide, a critical factor governing nanocrystal dimensions. This mechanism was validated in a pure bromide system, as illustrated in Figure S5. Increasing the amount of TOAB reduces the supersaturation of lead bromide, resulting in a redshift in the emission wavelength (Figures S5A and S5B), which indicates an increase in nanocrystal size. Conversely, when the TOAB concentration is held constant, an increase in DBSA raises the supersaturation level and induces a blueshift in the emission (Figures S5C and S5D), consistent with a reduction in nanocrystal size.

Besides the size regulation, the $CsPb(Br_xCl_{1-x})_3$ nanocrystals exhibit significantly altered composition. The accurate chlorine and bromine content was measured using ion chromatography. Under the identical halogen feed ratio in the precursors, the measured chlorine-to-bromine molar ratio of the nanocrystals decreases from 1.16 to 0.27 when 1.0 mL of DBSA-toluene mixture (1 g mL^{-1} for DBSA) was introduced in the precursor solution. It presents a gradually decreasing trend with increasing DBSA (Figure 2B; Table S1). Spherical aberration-corrected STEM analysis (Figure S6) reveals lattice parameter consistency between core and surface regions at atomic resolution, demonstrating a uniform halogen distribution throughout the nanocrystal structure. Such a significant difference in the chlorine-to-bromine molar ratio undoubtedly stems from variations in the halogen ratio within the nanocrystals rather than alterations in surface composition induced by DBSA. The decreased chlorine content originates from the reduced activity of chloride ions after introducing DBSA, resulting in lattice expansion of the nanocrystal because of the discrepancy in the ionic radius of chlorine and bromine. XRD analysis indicates that nanocrystals are crystalline in the cubic phase. The peak position corresponding to the (200) plane of the nanocrystals shifts toward a lower angle with the increased amount of DBSA (Figure 2C). These results signify the remarkable effect of DBSA ligands on regulating the size and composition of nanocrystals, thereby influencing overall optoelectronic behaviors. Notably, as the nanocrystals exhibit weak quantum confinement, the composition dominates the emission wavelength regulation.

Suppressing defects of nanocrystals synthesized with DBSA

Next, we investigated the optical properties of the nanocrystals with different sizes and compositions. While the reduction in nanocrystal size and the decrease in chlorine content exert opposing effects on the optical wavelength, the nanocrystal dimensions remain comparable to the exciton Bohr radius.

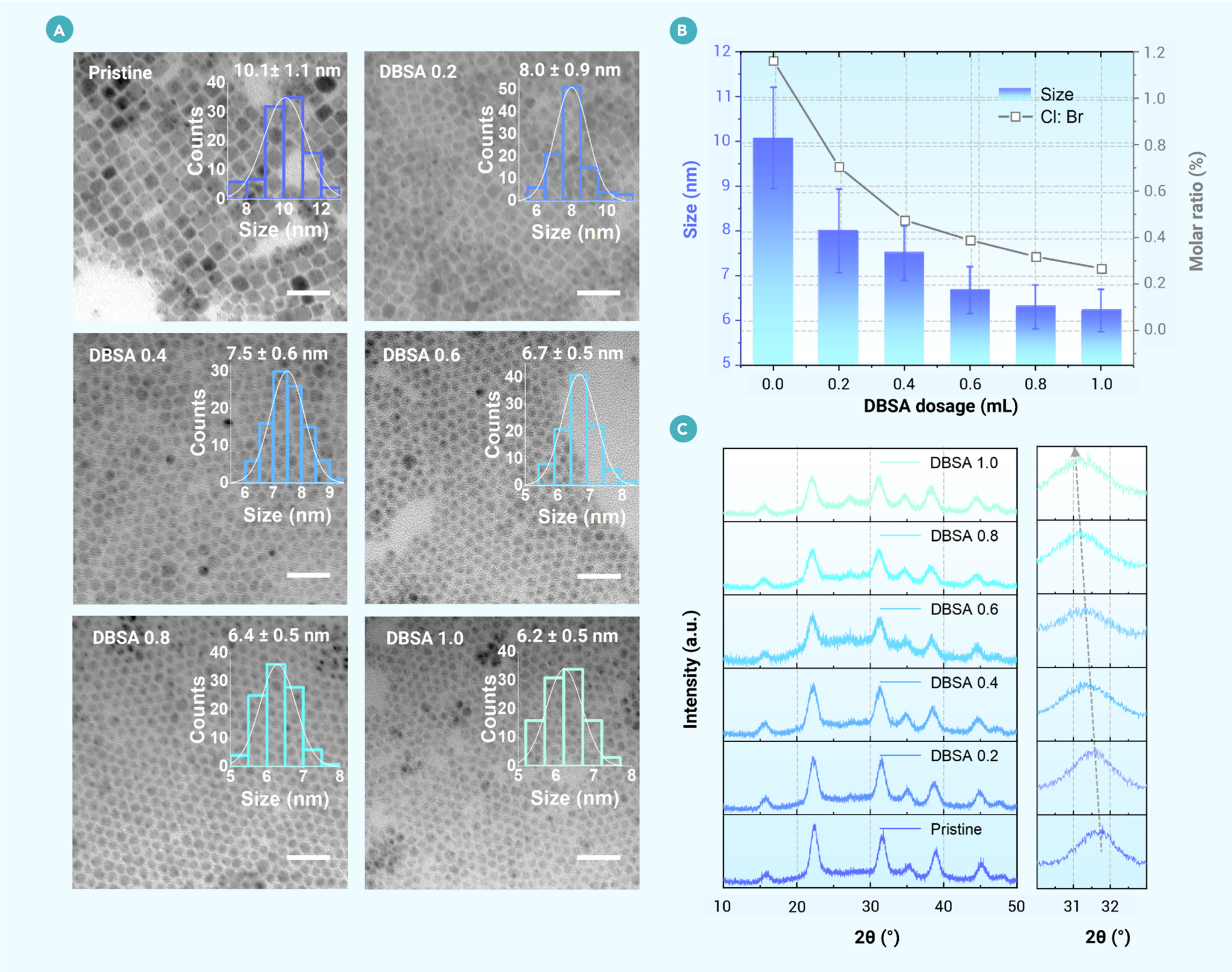

Figure 2. Size and compositional regulation of the $CsPb(Br_xCl_{1-x})_3$ nanocrystals synthesized with different DBSA dosages (A) Transmission electron microscope images of nanocrystals synthesized with different DBSA dosages. Scale bars, 30 nm. The insets show the corresponding size distribution histograms. (B) The dependences of nanocrystal size and chlorine-bromine ratio on DBSA dosage under the identical feeding ratio of other precursors. (C) XRD patterns of nanocrystals synthesized with different DBSA dosages. The right is an enlarged view belonging to the (200) plane.

Therefore, the halogen composition dominates the band-gap regulation. Consequently, with the increasing amount of DBSA additive in the precursors, both PL and UV-vis absorption spectra exhibit a substantial redshift and a strengthening of the first exciton absorption peaks (Figures 3A and S7).[43] The emission peak shifts from deep blue (450 nm) to sky blue (487 nm), verifying broad-spectrum tunability via DBSA adjustments. Remarkably, the PLQY of the nanocrystals increases from less than 20% to around 90% with the increasing amount of DBSA (Figure 3B). However, excessive DBSA causes critically low halide activity, impairing nanocrystal quality during synthesis. The ligands on the surface of nanocrystals treated with various DBSA dosages were investigated through quantitative FTIR characterizations (Figure S8). The results show that an increase in DBSA dosage has a minor influence on the surface ligands of nanocrystals, excluding the large discrepancy in surface defects between the series of nanocrystals.

To understand the improved PLQY, we first conducted temperature-dependent PL measurements to analyze the exciton binding energy (E_b) of nanocrystals. Four types of nanocrystals, referred to as pristine, DBSA 0.2, DBSA 0.6, and DBSA 1.0, were selected as the research objects to highlight the overall trend. From 80 to 300 K, the PL intensity of all nanocrystals exhibits a decreasing trend (Figure S9), correlating with non-radiative recombination resulting from thermally activated processes, such as exciton dissociation within nanocrystals.[44] As the amount of DBSA increases, the decline in PL intensity decelerates. The E_b of nanocrystals can be estimated through fitting with the Arrhenius formula[45]:

$$I_T = \frac{I_0}{1 + A\exp(-E_b/k_B T)},$$

where I_T and I_0 represent the integrated PL intensities at T and 0 K, respectively. A is a parameter associated with the cross-section of the binding state, and k_B stands for the Boltzmann constant. With the increased amount of DBSA, the E_b of the corresponding nanocrystals gradually increases (Figure 3C), consistent with the increase in the exciton absorption peak (Figure S7). Nevertheless, E_b is influenced by multiple factors, including nanocrystal sizes, composition, crystal structure, and surface characteristics, making it hard to establish a simple direct correlation between E_b and PLQY.

On the other hand, we observed a substantial PL redshift that contrasts with the expected size effect, attributed to the significantly reduced chlorine-to-bromine molar ratio in the nanocrystals. Therefore, the effect of reduced chlorine content on optical behaviors was further investigated. Transient PL and absorption experiments were conducted to study the carrier dynamics in $CsPb(Br_xCl_{1-x})_3$ nanocrystals with reduced chlorine content. Transient PL

www.the-innovation.org

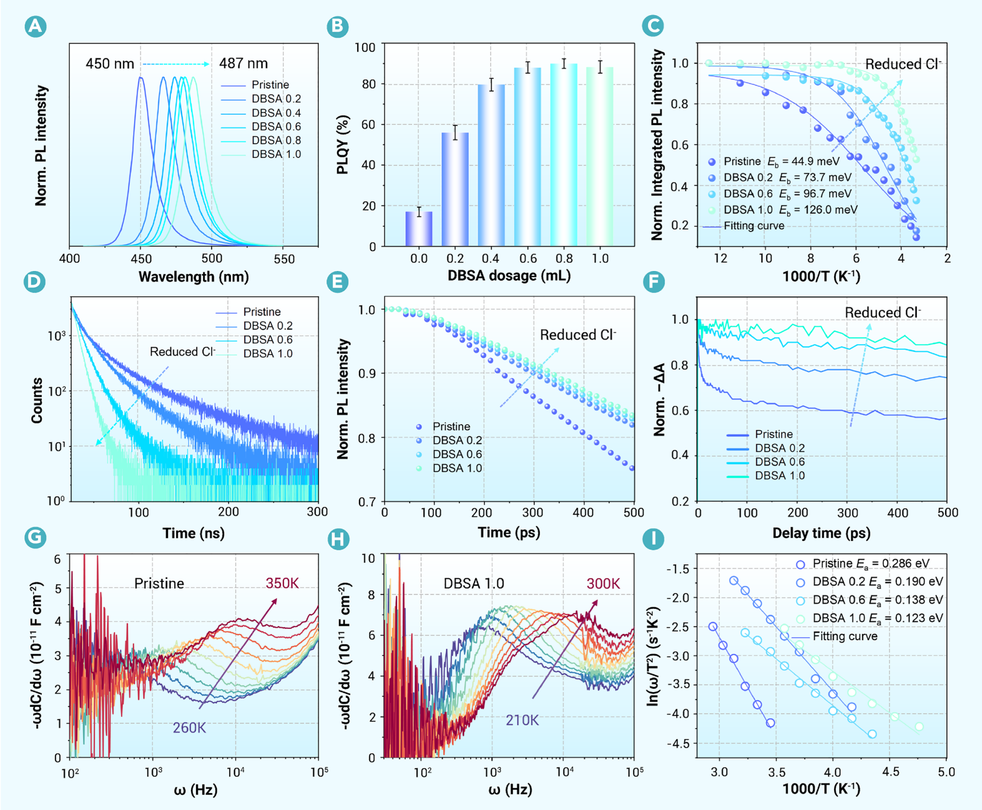

Figure 3. Optical behaviors of nanocrystals synthesized with different DBSA dosages (A) PL spectra. (B) PLQY of the nanocrystals synthesized with different DBSA dosages (DBSA added to toluene reaction precursor during nanocrystal synthesis before cesium precursor injection). (C) Temperature-dependence plots of integrated PL intensity vs. 1,000/T for extracting the E_b by fitting the Arrhenius equation. (D) Time-resolved PL decay spectra of the nanocrystals. (E) Normalized picosecond-resolution transient PL decay dynamics pumped with a 405 nm laser. (F) Normalized ground-state bleach kinetics extracted from TA spectra. Laser excitation: 405 nm, 100 kHz, 3.36 μJ cm^{-2}. (G and H) Derivatives of temperature-dependent capacitance vs. frequency plots for devices based on nanocrystals synthesized with pristine (G) and DBSA 1.0 mL (H). (I) The defect activation energy fitted by the Arrhenius formula extracted from thermal admittance spectra.

decays at the tens-of-nanoseconds timescale show a shortened average lifetime when reducing the chlorine content (Figure 3D), seemingly contradictory to the improved PLQY. Detailed analysis of transient PL reveals two decay channels, which can be well-fitted by the following bi-exponential decay function:

$$I = I_0 + A_1 \exp(-t/\tau_1) + A_2 \exp(-t/\tau_2),$$

where I_0 is a constant; the extracted decay lifetimes (τ_1 and τ_2) and the corresponding proportionality coefficients (A_1 and A_2) are shown in Figure S10. τ_1 shows a decrease from 12 to 6 ns with the increased DBSA (reduced chlorine content), while τ_2 is found to be largely dependent on the DBSA, decreasing from 61 to 19 ns when mixing 1 mL of DBSA in the reactant. The proportionality coefficients of the fast decay channel (A_1) increase, and the slow decay channel (A_2) decreases with the increased DBSA. The excitation power density-dependence test of PL lifetime (Figure S11) shows that the PL lifetimes (both τ_1 and τ_2 components) remain constant within the tested power range (0.1–2.0 μJ cm^{-2}). This independence strongly suggests that the dominant recombination mechanisms are monomolecular processes (exciton radiative recombination and defect-assisted recombination) rather than bimolecular or Auger processes, which would exhibit power-dependent lifetimes. In typical nanocrystals, previous research shows that the fast decay channel is usually considered the intrinsic excitonic emission and the slow one is related to trap-assisted recombination.[46,47] This differs from the bulk films, in which no excitonic emission was observed, and a longer decay lifetime is normally associated with a higher PLQY.[48,49] The shorter τ_2 and decreased A_2 for the nanocrystals synthesized with increased DBSA infer weaker carrier trapping and de-trapping processes. Transient PL measurements with picosecond resolution were conducted to validate the carrier-trapping process. The nanocrystals with reduced chlorine content exhibit slower PL decay on the hundred-picosecond timescale, namely, a weaker carrier-trapping contribution (Figure 3E). Furthermore, the TA decay kinetics of the nanocrystals show much slower ground-state bleach decay at the several-tens-of-picoseconds timescale under weak excitation conditions (Figures 3F and S12), verifying that more carriers remain at the band edge instead of being trapped by defects.[50,51] The excitation power-density-dependent PLQY measurements also support fewer carrier-trapping processes in the nanocrystals with reduced chlorine content (Figure S13).

To further pinpoint defect-state information in the nanocrystal layer, we conducted temperature-dependent admittance spectroscopy (TAS) on devices containing nanocrystals with different DBSA dosages.[30] The admittance spectra and derivative plots (−ωdC/dω vs. ω) measured at various temperatures are shown in Figures 3G, 3H, and S14. Distinct steps observed in the capacitance spectra across different temperatures correspond to carrier release and capture rates at defect states within the band gap. These characteristic defect-related responses shift toward higher frequencies with increasing temperature. By performing Arrhenius fitting, we determined the activation energy of these defects. The extracted defect-state distribution and activation energies from TAS measurements are presented in Figures 3I and S15. The results demonstrate that increasing DBSA content reduces the defect activation energy (E_a) from 0.286 (pristine) to 0.123 (DBSA 1.0 mL) eV, and the corresponding integrated tDOS decreases from 2.67 × 10^{15} to 6.47 × 10^{14} cm^{-3}. SCLC measurements on hole-only devices further support the DBSA-mediated reduction in defect-state density (Figure S16).[52]

It is important to clarify that the defects in perovskite nanocrystals are not exclusively surface localized. The chlorine-to-bromine molar ratio of nanocrystals decreases largely from 0.414 to 0.166 after introducing DBSA in the purified nanocrystals shown in Figure 1C, corresponding to at least a 17.5% chlorine deficiency relative to total halogens. However, the total surface halogen ratio in a nanocrystal with dimensions of 7 nm is calculated to be ∼15%. This confirms that defects in perovskite nanocrystals cannot be exclusively surface localized. Internal halogens can migrate to replenish surface vacancies, thereby generating internal defects. We infer that those nanocrystals contain both surface and internal defects, which are maintained in a dynamic equilibrium via processes such as ion migration. Besides, the DBSA-synthesized nanocrystals show nearly identical ligand coverage (Figure S8) while exhibiting improved PLQY when increasing the DBSA dosage from 0.2 to 0.6 mL, which primarily stems from suppressing chlorine-related internal defects.

Electroluminescence performance of the nanocrystals

LEDs consisting of multilayer structures (Figure 4A) were fabricated to investigate the electroluminescence performance of the nanocrystals. In this structure, precisely controlled $CsPb(Br_xCl_{1-x})_3$ nanocrystal monolayers were deposited as the emissive layer to converge carrier injection while avoiding the high-resistance carrier-transporting process in the nanocrystal film. The nickel oxide interlayer, combined with the PFI-doped PEDOT:PSS, ensures efficient hole injection. The multilayer hole transport layer (HTL) and electron transport layer (ETL) are integrated to balance carrier injection and confine excitons, aiming to achieve high-performance blue perovskite nanocrystal LEDs with high EQE.

All the nanocrystals exhibit stable electroluminescence (EL) with emission peaks centered at 448, 462, 470, 474, 478, and 482 nm, with the increased

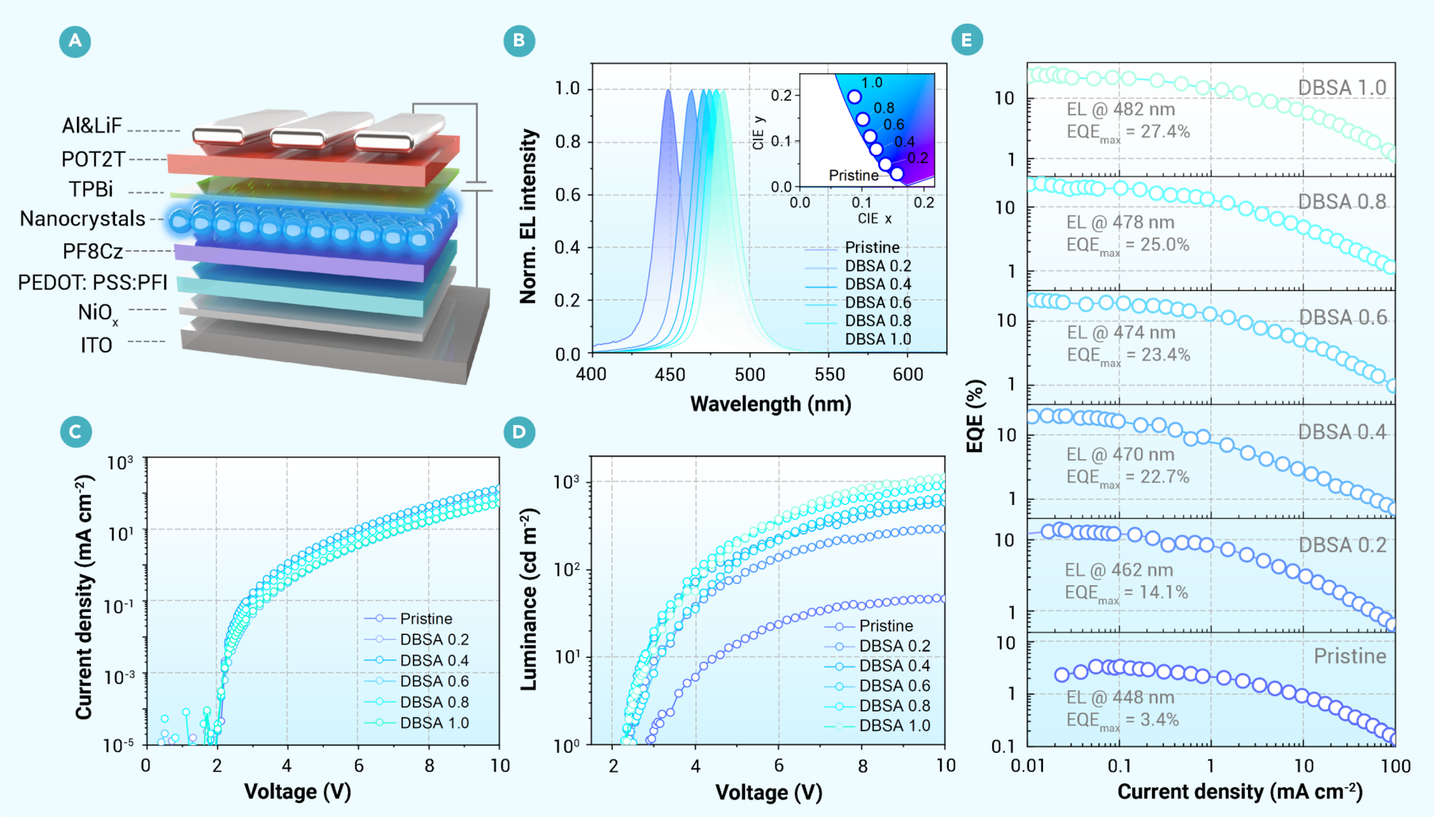

Figure 4. Electroluminescence performance of the LEDs based on nanocrystals synthesized with different DBSA dosages (A) Schematic of the device structure, showing the multilayers of indium tin oxide (ITO; ~60 nm), NiO_x (~3 nm), PEDOT:PSS:PFI (~35 nm), PF8Cz (~25 nm), $CsPb(Br_xCl_{1-x})_3$ nanocrystal monolayer (~6–10 nm), TPBi (~5 nm), PO-T2T (~45 nm), LiF (~1 nm), and aluminum (Al; ~100 nm). (B) Normalized EL spectra of the LEDs, with an inset of corresponding CIE color coordinates. The relatively small current density of the LEDs is caused by the insulating PFI. (C and D) Typical current density-voltage (C) and luminance-voltage (D) characteristics of the LEDs. (E) EQE-current density curves of the LEDs.

amount of DBSA from 0 to 1 mL (Figure 4B), corresponding to the CIE chromatic coordinates of (0.157, 0.028), (0.138, 0.049), (0.123, 0.082), (0.113, 0.110), (0.102, 0.147), and (0.088, 0.196), respectively. The typical current density-luminance-voltage curves of those devices are shown in Figures 4C and 4D, illustrating similar current density-voltage characteristics and nearly identical turn-on voltages of 2.3–2.5 V. The result suggests analogous carrier injection and transport behaviors, consistent with the comparable band structure of the $CsPb(Br_xCl_{1-x})_3$ nanocrystals measured from the UV photoelectron spectroscopy (UPS) characterizations (Figure S17). Interestingly, the turn-on voltage of the device is lower than the emitted photon energy. This phenomenon originates from the synergistic effects of the unique electron-phonon coupling properties of perovskite and balanced carrier injection in the device.[53,54] Notably, the luminance of the LEDs was enhanced significantly under a constant voltage for the nanocrystals synthesized with the increased amount of DBSA, even after considering the luminance calibration with human visual function (Figure S18). Consequently, the maximum EQEs reached 3.4% (448 nm), 14.1% (462 nm), 22.7% (470 nm), 23.4% (474 nm), 24.0% (478 nm), and 27.4% (482 nm), increasing monotonically with the reduced chlorine content (Figure 4E). The results indicate that the electroluminescence performance of nanocrystals is closely related to deep-level defects associated with chlorine. Given that the surface ligands of the nanocrystals are nearly identical, their electroluminescence behavior is primarily governed by the defects modulated by DBSA.

To exclude the influence of varying energy-level alignment resulting from different nanocrystal band gaps and device structures, and thereby highlight the critical role of defect suppression in enhancing device performance, two representative $CsPb(Br_xCl_{1-x})_3$ nanocrystals with the emission peak centered at 470 nm were synthesized with or without the inclusion of DBSA, accompanied by adjusting the feeding amounts of precursors (Table S2). The surface defects of the nanocrystals synthesized without DBSA can be effectively passivated with DDA^+ and ETBPB additives. The absorption and PL spectra of the two nanocrystals are shown in Figure 5A, where the nanocrystal synthesized with DBSA (DBSA-nanocrystal) exhibits a strong exciton absorption peak, attributed to the reduction in size and the improvement in size uniformity. In terms of optical properties, the DBSA-nanocrystal presents much slower ground-state bleach decay in the TA decay kinetics (Figures 5B and S19) and a shorter average PL lifetime at the tens-of-nanoseconds timescale (Figure S20), verifying fewer defects generated under the DBSA ligand-participating synthesis. In electroluminescence performance, the maximum luminance reaches 1,000 cd m^{-2} for the LEDs based on the DBSA-nanocrystal, much brighter than the device based on the control nanocrystal (Figure 5C). Regarding efficiency, the maximum EQE of the champion LEDs based on the DBSA-nanocrystal reaches 24.5% (Figure 5D), representing the highest value achieved for pure-blue emission in perovskite LEDs to date (Figure S21). In statistics, the histogram of the peak EQEs shows an average EQE of 23.4% with a small deviation of 0.56% (Figure 5E), two times higher than the average EQE of the counterpart devices. A maximum EQE of 24.0% was also certified through third-party certification (Figure S22), comparable to that measured in the laboratory. The corresponding EQE values at 100 and 1,000 cd m^{-2} are approximately 15% and 2%, respectively. Notably, the operational stability of the devices shows significant improvement (Figure S23). Spectral stability is maintained even under extreme conditions, as evidenced by invariant emission peaks at 470 nm when increasing the driving voltage to 10 V or under continuous driving (Figure 5F). Furthermore, reducing Cl content in precursors to mitigate Cl defects during the nucleation process and subsequent Cl supplementation with DDAC constitutes a promising approach for nanocrystal synthesis. The corresponding LEDs also demonstrate high peak EQEs (Figure S24). However, excessive quaternary ammonium ligands (DDA^+) may induce more pronounced efficiency roll-off in the devices and worse operational stability (Figure S25). The superiority in optical behavior and device performance of the DBSA-nanocrystal compellingly demonstrates the advancement of ligands in modulating the surface and internal defects of nanocrystals.

www.the-innovation.org

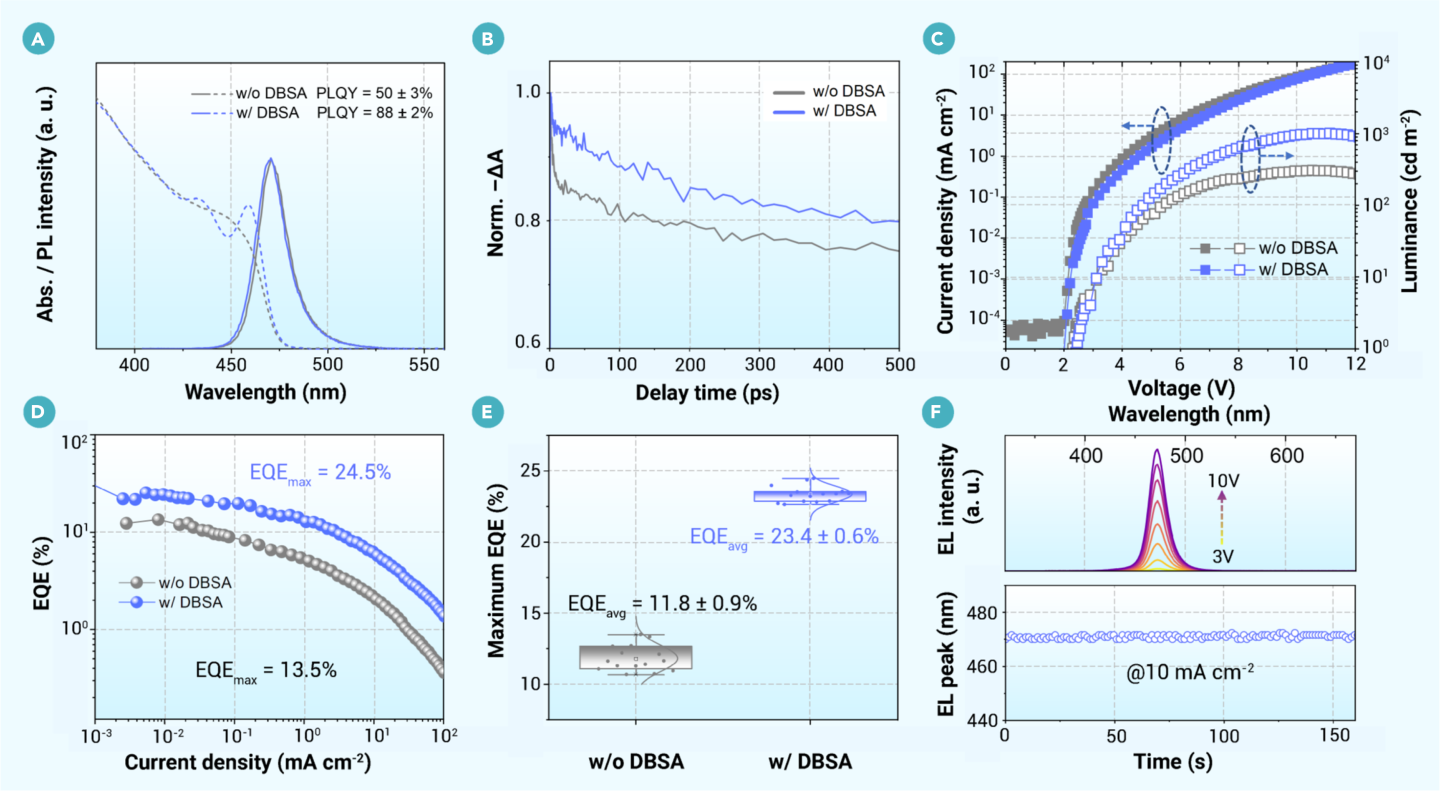

Figure 5. The performance comparison of the nanocrystals at an identical emission (470 nm), synthesized with or without DBSA (A) Absorption and PL spectra of the two nanocrystals. (B) Normalized ground-state bleach kinetics extracted from TA spectra. Laser excitation: 405 nm, 100 kHz, 3.36 μJ cm^{-2}. (C and D) Typical current density-voltage-luminance characteristics (C) and EQE-current density curves of the LEDs (D) based on the two nanocrystals. (E) Maximum EQE statistics of LEDs (15 devices each). (F) Voltage-dependent EL spectra and time-dependent EL peak of the LEDs based on DBSA-nanocrystal.

CONCLUSION

In summary, we have demonstrated the surface-ligand-triggered synthetic control of defects in nanocrystals. The strong acid ligand DBSA ionizes to DBS$^-$ and H$^+$ in low-polarity solvents and interacts with quaternary ammonium and halogens, respectively, thereby modulating the activity of halogen. This unveils a new functionality of acid ligand, which allows for controllable regulation of the nanocrystal composition, and thus suppresses the formation of halogen-related internal defects. Leveraging these insights, blue-emissive nanocrystals centered at 470 nm achieve a PLQY of ~90% and yield blue perovskite LEDs with champion EQEs of up to 24.5%, setting a new benchmark for pure-blue perovskite LEDs. Further investigations into surface ligands for controlling nanocrystal surfaces and internal defects are expected to offer exciting possibilities in advanced optoelectronic applications.

RESOURCE AVAILABILITY

Materials availability

All materials used in this study are commercially available or can be synthesized according to the procedures described in the materials and methods.

Data and code availability

The authors declare that the main data supporting the findings of this study are available within the paper and its supplemental information. Extra data are available upon reasonable request from the corresponding author.

FUNDING AND ACKNOWLEDGMENTS

This work was financially supported by the "Pioneer" and "Leading Goose" R&D Program of Zhejiang (2024C01191, X.D.), the Fundamental Research Funds for the Central Universities (2024QZJH10, X.D.), and the National Natural Science Foundation of China (U22A20133, Z.Y.). X.D. gratefully acknowledges the support of the Zhejiang University Education Foundation Qizhen Scholar Foundation.

AUTHOR CONTRIBUTIONS

X.D. and Q. Cao conceived the idea and designed the experiments. X.D. supervised the work. Q. Cao synthesized the nanocrystals, fabricated the device, and performed optical measurements. Y.F. and H.Z. assisted in composition and crystal structure characterizations. Q. Cai and Y.G. assisted in the synthesis of nanocrystals and optical measurements. D.Z. and Y.H. assisted in the device fabrication and characterization. X.W. assisted in the temperature-dependent PL measurements. M.Z., H.H., and Z.Y. provided helpful discussions. Q. Cao wrote the original draft of the manuscript. X.D. revised the manuscript. All authors contributed to the manuscript and approved the final version.

DECLARATION OF INTERESTS

The authors declare no competing interests.

SUPPLEMENTAL INFORMATION

It can be found online at https://doi.org/10.1016/j.xinn.2026.101273.

REFERENCES

1. Shamsi, J., Urban, A.S., Imran, M. et al. (2019). Metal Halide Perovskite Nanocrystals: Synthesis, Post-Synthesis Modifications, and Their Optical Properties. *Chem. Rev.* **119**:3296–3348. DOI:10.1021/acs.chemrev.8b00644
2. Almutlaq, J., Liu, Y., Mir, W.J. et al. (2024). Engineering colloidal semiconductor nanocrystals for quantum information processing. *Nat. Nanotechnol.* **19**:1091–1100. DOI:10.1038/s41565-024-01606-4
3. Jang, K.Y., Chang, S.E., Kim, D.H. et al. (2025). Nanocrystalline Perovskites for Bright and Efficient Light-Emitting Diodes. *Adv. Mater.* **37**:2415648. DOI:10.1002/adma.202415648
4. Liu, X.-K., Xu, W., Bai, S. et al. (2021). Metal halide perovskites for light-emitting diodes. *Nat. Mater.* **20**:10–21. DOI:10.1038/s41563-020-0784-7
5. Dey, A., Ye, J., De, A. et al. (2021). State of the Art and Prospects for Halide Perovskite Nanocrystals. *ACS Nano* **15**:10775–10981. DOI:10.1021/acsnano.0c08903
6. Yang, J.-N., Wang, J.-J., Yin, Y.-C. et al. (2023). Mitigating halide ion migration by resurfacing lead halide perovskite nanocrystals for stable light-emitting diodes. *Chem. Soc. Rev.* **52**:5516–5540. DOI:10.1039/d3cs00179b
7. Han, T.-H., Jang, K.Y., Dong, Y. et al. (2022). A roadmap for the commercialization of perovskite light emitters. *Nat. Rev. Mater.* **7**:757–777. DOI:10.1038/s41578-022-00459-4
8. Zhao, B., Vasilopoulou, M., Fakharuddin, A. et al. (2023). Light management for perovskite light-emitting diodes. *Nat. Nanotechnol.* **18**:981–992. DOI:10.1038/s41565-023-01482-4
9. Zheng, X., Yuan, S., Liu, J. et al. (2020). Chlorine Vacancy Passivation in Mixed Halide Perovskite Quantum Dots by Organic Pseudohalides Enables Efficient Rec. 2020 Blue Light-Emitting Diodes. *ACS Energy Lett.* **5**:793–798. DOI:10.1021/acsenergylett.0c00057

10. Akkerman, Q.A., Motti, S.G., Srimath Kandada, A.R. et al. (2016). Solution Synthesis Approach to Colloidal Cesium Lead Halide Perovskite Nanoplatelets with Monolayer-Level Thickness Control. *J. Am. Chem. Soc.* **138**:1010–1016. DOI:10.1021/jacs.5b12124
11. Wang, N., Cheng, L., Ge, R. et al. (2016). Perovskite light-emitting diodes based on solution-processed self-organized multiple quantum wells. *Nat. Photonics* **10**:699–704. DOI:10.1038/nphoton.2016.185
12. Dong, Y., Wang, Y.-K., Yuan, F. et al. (2020). Bipolar-shell resurfacing for blue LEDs based on strongly confined perovskite quantum dots. *Nat. Nanotechnol.* **15**:668–674. DOI:10.1038/s41565-020-0714-5
13. Bi, C., Yao, Z., Hu, J. et al. (2022). Suppressing Auger Recombination of Perovskite Quantum Dots for Efficient Pure-Blue-Light-Emitting Diodes. *ACS Energy Lett.* **8**:731–739. DOI:10.1021/acsenergylett.2c02613
14. Jang, K.Y., Hwang, S.Y., Woo, S.-J. et al. (2024). Efficient Deep-Blue Light-Emitting Diodes Through Decoupling of Colloidal Perovskite Quantum Dots. *Adv. Mater.* **36**:2404856. DOI:10.1002/adma.202404856
15. Song, J., Li, J., Li, X. et al. (2015). Quantum Dot Light-Emitting Diodes Based on Inorganic Perovskite Cesium Lead Halides ($CsPbX_3$). *Adv. Mater.* **27**:7162–7167. DOI:10.1002/adma.201502567
16. Karlsson, M., Yi, Z., Reichert, S. et al. (2021). Mixed halide perovskites for spectrally stable and high-efficiency blue light-emitting diodes. *Nat. Commun.* **12**:361. DOI:10.1038/s41467-020-20582-6
17. Nenon, D.P., Pressler, K., Kang, J. et al. (2018). Design Principles for Trap-Free $CsPbX_3$ Nanocrystals: Enumerating and Eliminating Surface Halide Vacancies with Softer Lewis Bases. *J. Am. Chem. Soc.* **140**:17760–17772. DOI:10.1021/jacs.8b11035
18. Gao, Y., Cai, Q., He, Y. et al. (2024). Highly efficient blue light-emitting diodes based on mixed-halide perovskites with reduced chlorine defects. *Sci. Adv.* **10**:eado5645. DOI:10.1126/sciadv.ado5645
19. Boles, M.A., Ling, D., Hyeon, T. et al. (2016). The surface science of nanocrystals. *Nat. Mater.* **15**:141–153. DOI:10.1038/nmat4526
20. Calvin, J.J., Brewer, A.S. and Alivisatos, A.P. (2022). The role of organic ligand shell structures in colloidal nanocrystal synthesis. *Nat. Synth.* **1**:127–137. DOI:10.1038/s44160-022-00025-4
21. Peng, X. (2009). An essay on synthetic chemistry of colloidal nanocrystals. *Nano Res.* **2**:425–447. DOI:10.1007/s12274-009-9047-2
22. Bera, S., Behera, R.K. and Pradhan, N. (2020). α-Halo Ketone for Polyhedral Perovskite Nanocrystals: Evolutions, Shape Conversions, Ligand Chemistry, and Self-Assembly. *J. Am. Chem. Soc.* **142**:20865–20874. DOI:10.1021/jacs.0c10688
23. Brown, A.A.M., Vashishtha, P., Hooper, T.J.N. et al. (2021). Precise Control of $CsPbBr_3$ Perovskite Nanocrystal Growth at Room Temperature: Size Tunability and Synthetic Insights. *Chem. Mater.* **33**:2387–2397. DOI:10.1021/acs.chemmater.0c04569
24. Long, Z., Liu, M., Wu, X.-g. et al. (2023). A reactivity-controlled epitaxial growth strategy for synthesizing large nanocrystals. *Nat. Synth.* **2**:296–304. DOI:10.1038/s44160-022-00210-5
25. Bai, Y., Hao, M., Ding, S. et al. (2022). Surface Chemistry Engineering of Perovskite Quantum Dots: Strategies, Applications, and Perspectives. *Adv. Mater.* **34**:2105958. DOI:10.1002/adma.202105958
26. Wang, Y.-K., Singh, K., Li, J.-Y. et al. (2022). In Situ Inorganic Ligand Replenishment Enables Bandgap Stability in Mixed-Halide Perovskite Quantum Dot Solids. *Adv. Mater.* **34**:2200854. DOI:10.1002/adma.202200854
27. Liang, S., Zhang, M., He, S. et al. (2023). Metal halide perovskite nanorods with tailored dimensions, compositions and stabilities. *Nat. Synth.* **2**:719–728. DOI:10.1038/s44160-023-00307-5
28. de Mello, J.C., Wittmann, H.F. and Friend, R.H. (2004). An improved experimental determination of external photoluminescence quantum efficiency. *Adv. Mater.* **9**:230–232. DOI:10.1002/adma.19970090308
29. Zhang, Z., Ye, Y., Pu, C. et al. (2018). High-Performance, Solution-Processed, and Insulating-Layer-Free Light-Emitting Diodes Based on Colloidal Quantum Dots. *Adv. Mater.* **30**:1801387. DOI:10.1002/adma.201801387
30. Bao, C. and Gao, F. (2022). Physics of defects in metal halide perovskites. *Rep. Prog. Phys.* **85**:096501. DOI:10.1088/1361-6633/ac7c7a
31. Dai, X., Zhang, Z., Jin, Y. et al. (2014). Solution-processed, high-performance light-emitting diodes based on quantum dots. *Nature* **515**:96–99. DOI:10.1038/nature13829
32. Song, J., Li, J., Xu, L. et al. (2018). Room-Temperature Triple-Ligand Surface Engineering Synergistically Boosts Ink Stability, Recombination Dynamics, and Charge Injection toward EQE-11.6% Perovskite QLEDs. *Adv. Mater.* **30**:1800764. DOI:10.1002/adma.201800764
33. Chen, F., Liu, Y., Zhang, D. et al. (2023). Bilayer phosphine oxide modification toward efficient and large-area pure-blue perovskite quantum dot light-emitting diodes. *Sci. Bull.* **68**:2354–2361. DOI:10.1016/j.scib.2023.09.014
34. Shynkarenko, Y., Bodnarchuk, M.I., Bernasconi, C. et al. (2019). Direct Synthesis of Quaternary Alkylammonium-Capped Perovskite Nanocrystals for Efficient Blue and Green Light-Emitting Diodes. *ACS Energy Lett.* **4**:2703–2711. DOI:10.1021/acsenergylett.9b01915
35. Zaccaria, F., Zhang, B., Goldoni, L. et al. (2022). The Reactivity of $CsPbBr_3$ Nanocrystals toward Acid/Base Ligands. *ACS Nano* **16**:1444–1455. DOI:10.1021/acsnano.1c09603
36. Li, D., Lyu, B., Sun, J. et al. (2024). Ligands Optimization Governed by Solubility Principles for Pure Blue Emission in Mixed-Halide Perovskite LEDs. *ACS Energy Lett.* **9**:3261–3268. DOI:10.1021/acsenergylett.4c00881
37. Yang, D., Li, X., Zhou, W. et al. (2019). $CsPbBr_3$ Quantum Dots 2.0: Benzenesulfonic Acid Equivalent Ligand Awakens Complete Purification. *Adv. Mater.* **31**:e1900767. DOI:10.1002/adma.201900767
38. Fang, T., Yuan, S., Li, X. et al. (2024). Sulfonate Additive Simultaneously Suppresses Interstitials and Vacancies Toward Efficient and Stable Perovskite Quantum Dot LEDs. *Adv. Opt. Mater.* **12**:2302253. DOI:10.1002/adom.202302253
39. Xu, B., Yuan, S., Wang, L. et al. (2025). Highly Efficient Blue Light-Emitting Diodes Enabled by Gradient Core/Shell-Structured Perovskite Quantum Dots. *ACS Nano* **19**:3694–3704. DOI:10.1021/acsnano.4c14276
40. Liu, Y., Li, Y., Hu, X. et al. (2023). Ligands for CsPbBr3 perovskite quantum dots: The stronger the better? *Chem. Eng. J.* **453**:139904. DOI:10.1016/j.cej.2022.139904
41. Allen, L.C. (1989). Electronegativity is the average one-electron energy of the valence-shell electrons in ground-state free atoms. *J. Am. Chem. Soc.* **111**:9003–9014. DOI:10.1021/ja00207a003
42. Thanh, N.T.K., Maclean, N. and Mahiddine, S. (2014). Mechanisms of Nucleation and Growth of Nanoparticles in Solution. *Chem. Rev.* **114**:7610–7630. DOI:10.1021/cr400544s
43. Akkerman, Q.A. (2022). Spheroidal Cesium Lead Chloride–Bromide Quantum Dots and a Fast Determination of Their Size and Halide Content. *Nano Lett.* **22**:8168–8173. DOI:10.1021/acs.nanolett.2c02601
44. Zhu, H., Tong, G., Li, J. et al. (2022). Enriched-Bromine Surface State for Stable Sky-Blue Spectrum Perovskite QLEDs With an EQE of 14.6%. *Adv. Mater.* **34**:2205092. DOI:10.1002/adma.202205092
45. Savenije, T.J., Ponseca, C.S., Kunneman, L. et al. (2014). Thermally Activated Exciton Dissociation and Recombination Control the Carrier Dynamics in Organometal Halide Perovskite. *J. Phys. Chem. Lett.* **5**:2189–2194. DOI:10.1021/jz500858a
46. Gao, Y. and Peng, X. (2015). Photogenerated Excitons in Plain Core CdSe Nanocrystals with Unity Radiative Decay in Single Channel: The Effects of Surface and Ligands. *J. Am. Chem. Soc.* **137**:4230–4235. DOI:10.1021/jacs.5b01314
47. Zhang, X., Huang, H., Jin, L. et al. (2023). Ligand-Assisted Coupling Manipulation for Efficient and Stable $FAPbI_3$ Colloidal Quantum Dot Solar Cells. *Angew. Chem. Int. Ed.* **62**:e202214241. DOI:10.1002/anie.202214241
48. Cho, H., Kim, J.S., Wolf, C. et al. (2018). High-Efficiency Polycrystalline Perovskite Light-Emitting Diodes Based on Mixed Cations. *ACS Nano* **12**:2883–2892. DOI:10.1021/acsnano.8b00409
49. Miao, Y., Ke, Y., Wang, N. et al. (2019). Stable and bright formamidinium-based perovskite light-emitting diodes with high energy conversion efficiency. *Nat. Commun.* **10**:3624. DOI:10.1038/s41467-019-11567-1
50. Xu, L., Li, J., Cai, B. et al. (2020). A bilateral interfacial passivation strategy promoting efficiency and stability of perovskite quantum dot light-emitting diodes. *Nat. Commun.* **11**:3902. DOI:10.1038/s41467-020-17633-3
51. Li, H., Feng, Y., Zhu, M. et al. (2024). Nanosurface-reconstructed perovskite for highly efficient and stable active-matrix light-emitting diode display. *Nat. Nanotechnol.* **19**:638–645. DOI:10.1038/s41565-024-01652-y
52. Duijnstee, E.A., Ball, J.M., Le Corre, V.M. et al. (2020). Toward Understanding Space-Charge Limited Current Measurements on Metal Halide Perovskites. *ACS Energy Lett.* **5**:376–384. DOI:10.1021/acsenergylett.9b02720
53. Ha, S.-T., Shen, C., Zhang, J. et al. (2016). Laser cooling of organic–inorganic lead halide perovskites. *Nat. Photonics* **10**:115–121. DOI:10.1038/nphoton.2015.243
54. Morozov, Y.V., Zhang, S., Brennan, M.C. et al. (2017). Photoluminescence Up-Conversion in $CsPbBr_3$ Nanocrystals. *ACS Energy Lett.* **2**:2514–2515. DOI:10.1021/acsenergylett.7b00902

The Innovation, Volume 7

Supplemental Information

Surface-ligand-triggered synthetic control of defects in nanocrystals toward high-efficiency blue electroluminescence

Qingli Cao, Qiuting Cai, Yifeng Feng, Xinyang Wang, Dingshuo Zhang, Yun Gao, Haoran Zhang, Meiyi Zhu, Yifan He, Haiping He, Zhizhen Ye, and Xingliang Dai

SUPPLEMENTAL INFORMATION

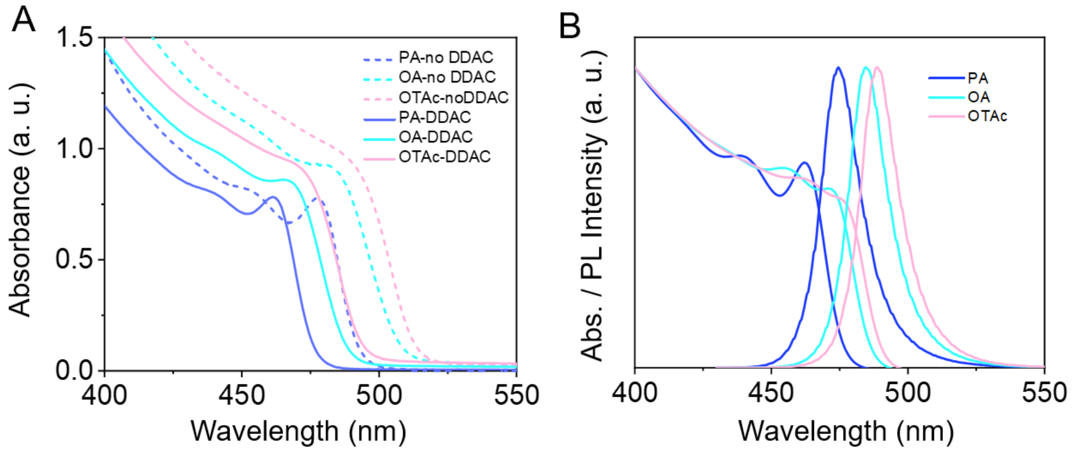

Figure S1. (A) Absorption spectra of nanocrystal crude solution before and after DDAC treatment. The nanocrystals were synthesized using cesium precursors dissolved in different acids (OA = oleic acid, OTAc = octanoic acid). (B) Absorption and PL spectra of purified nanocrystals using different acids under the same chlorine-bromine feeding ratio.

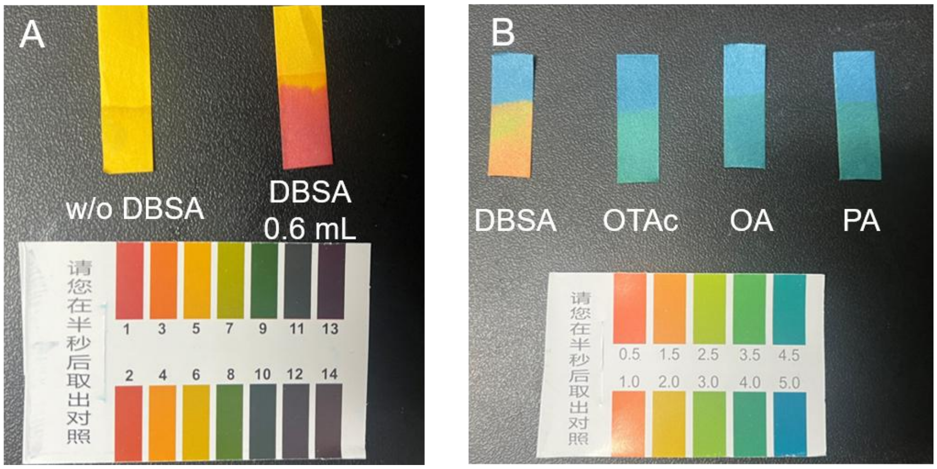

Figure S2. (A) Comparison of pH values before and after adding DBSA (1 g mL^{-1}) to the precursor solution. (B) Comparison of pH values of different acids diluted in toluene (0.2 M).

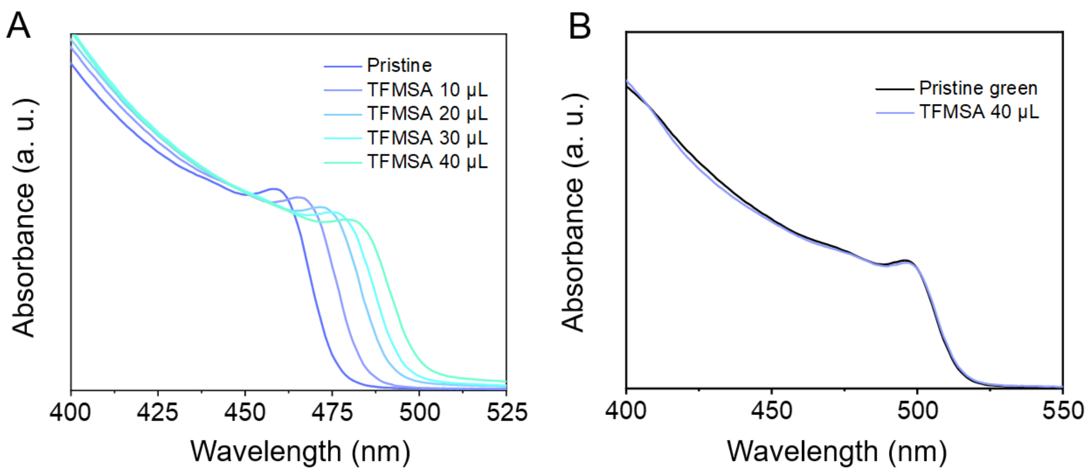

Figure S3. (A) Absorption spectra of different amounts of TFMSA added to the crude $CsPb(Br_xCl_{1-x})_3$ nanocrystal solution without purification. (B) Absorption spectra of crude $CsPbBr_3$ nanocrystal solution without purification before or after inclusion of 40 µL TFMAS.

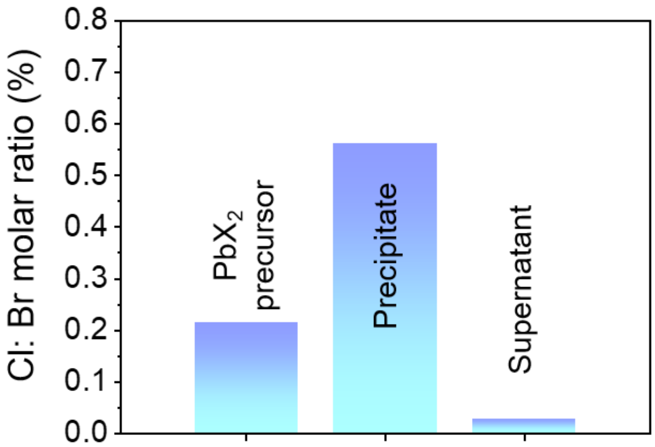

Figure S4. The chlorine-bromine ratio of the precursor solution, precipitate, and supernatant measured by ion chromatography.

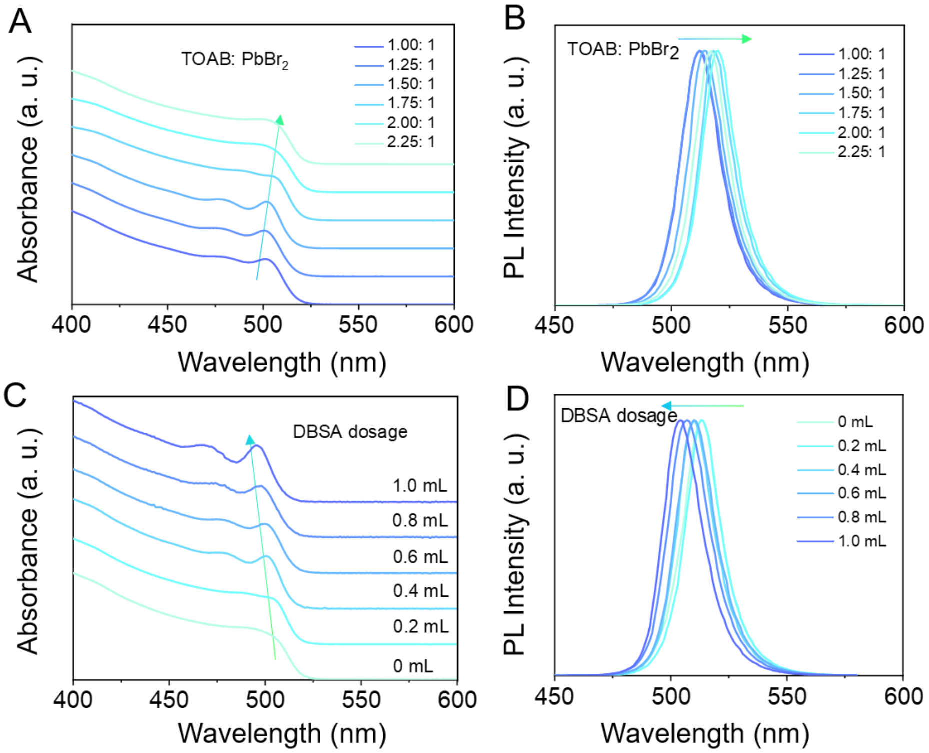

Figure S5. (A, B) Absorption and PL spectra of $CsPbBr_3$ nanocrystals synthesized with different TOAB dosages (DBSA dosage = 0.4 mL). (C, D) Absorption and PL spectra of $CsPbBr_3$ nanocrystals synthesized with different DBSA dosages (TOAB: $PbBr_2$ = 1: 1.5).

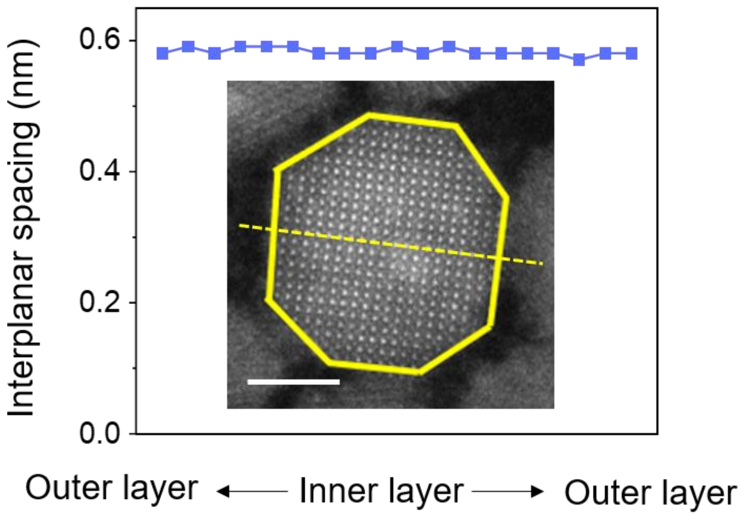

Figure S6. Interplanar spacing as a function of layer position in the crystal lattice of a nanocrystal. The insets correspond to a spherical aberration-corrected HAADF-STEM image of a nanocrystal. Scale bar: 5 nm.

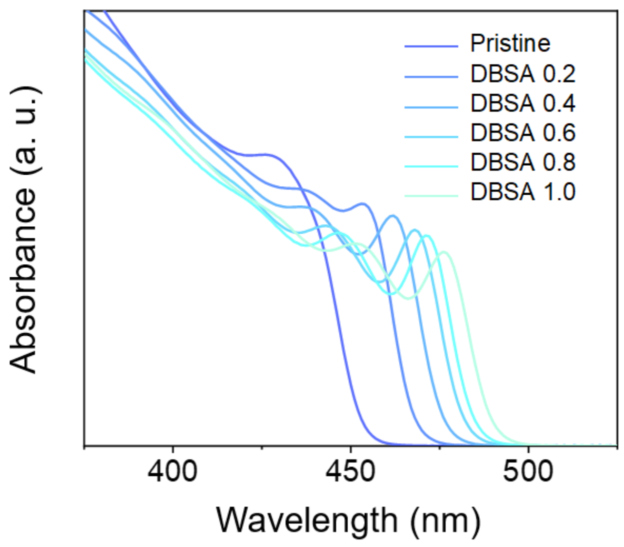

Figure S7. Absorption spectra of nanocrystals synthesized with different DBSA dosages added to the precursor solution before Cs-PA triggered the reaction.

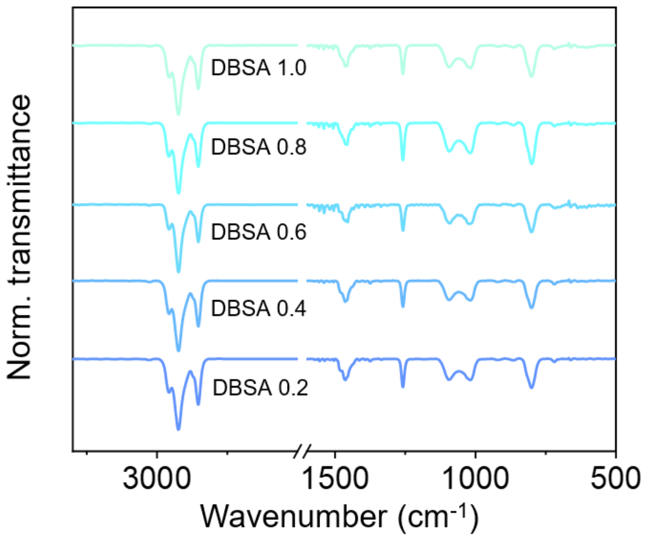

Figure S8. Fourier transform infrared spectra of the nanocrystals synthesized with different DBSA dosages.

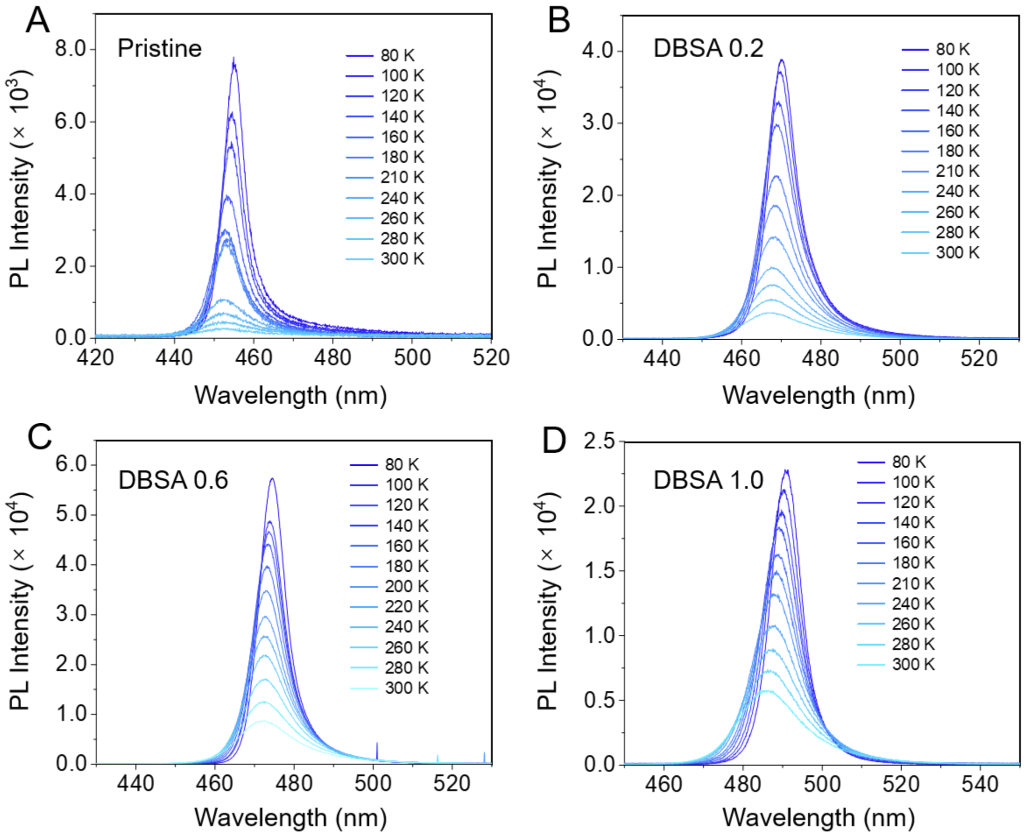

Figure S9. Temperature-dependent PL spectra of nanocrystals synthesized with different DBSA dosages from 80 K to 300 K with an excitation power density of about 5 mW cm^{-1}: pristine (A), DBSA 0.2 ml (B), DBSA 0.6 ml (C), DBSA 1.0 ml (D).

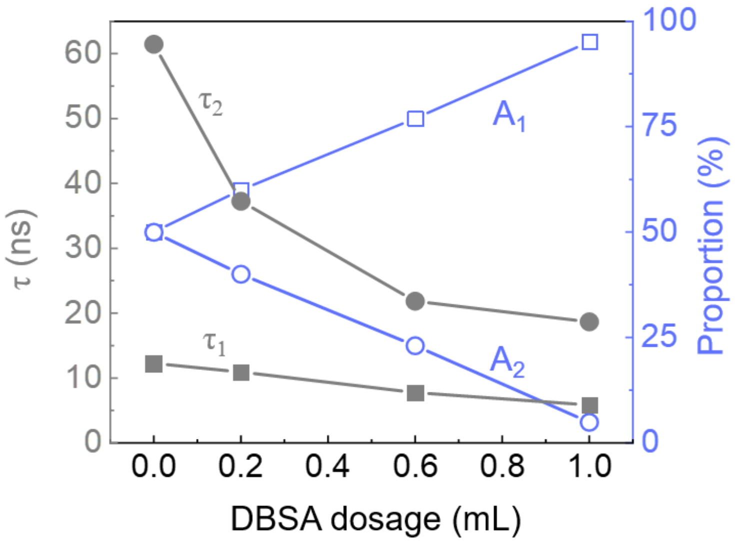

Figure S10. The extracted lifetimes (τ_1, τ_2) and proportions (A_1, A_2) fitted with a double exponential function from time-resolved PL decay spectra of nanocrystals synthesized with different DBSA dosages.

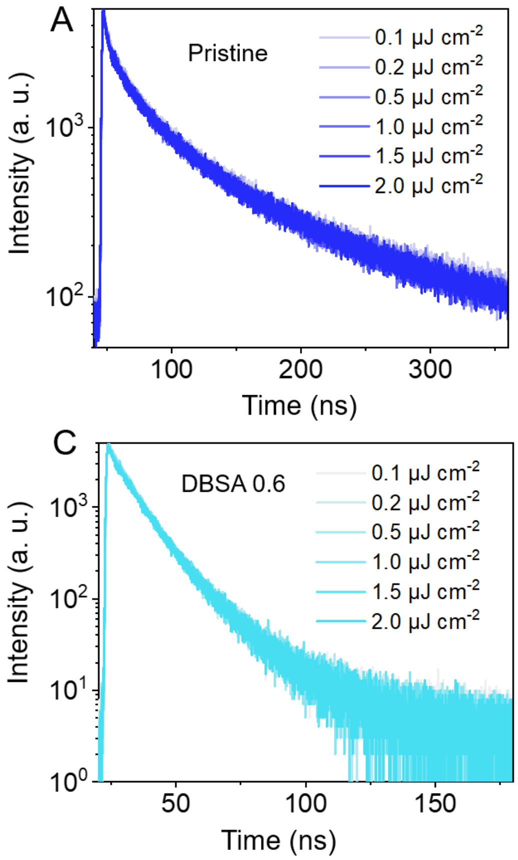

Figure S11. The excitation power density-dependent transient PL decay of the nanocrystals synthesized with different DBSA dosages: pristine (A), DBSA 0.2 mL (B), DBSA 0.6 mL (C), DBSA 1.0 mL (D).

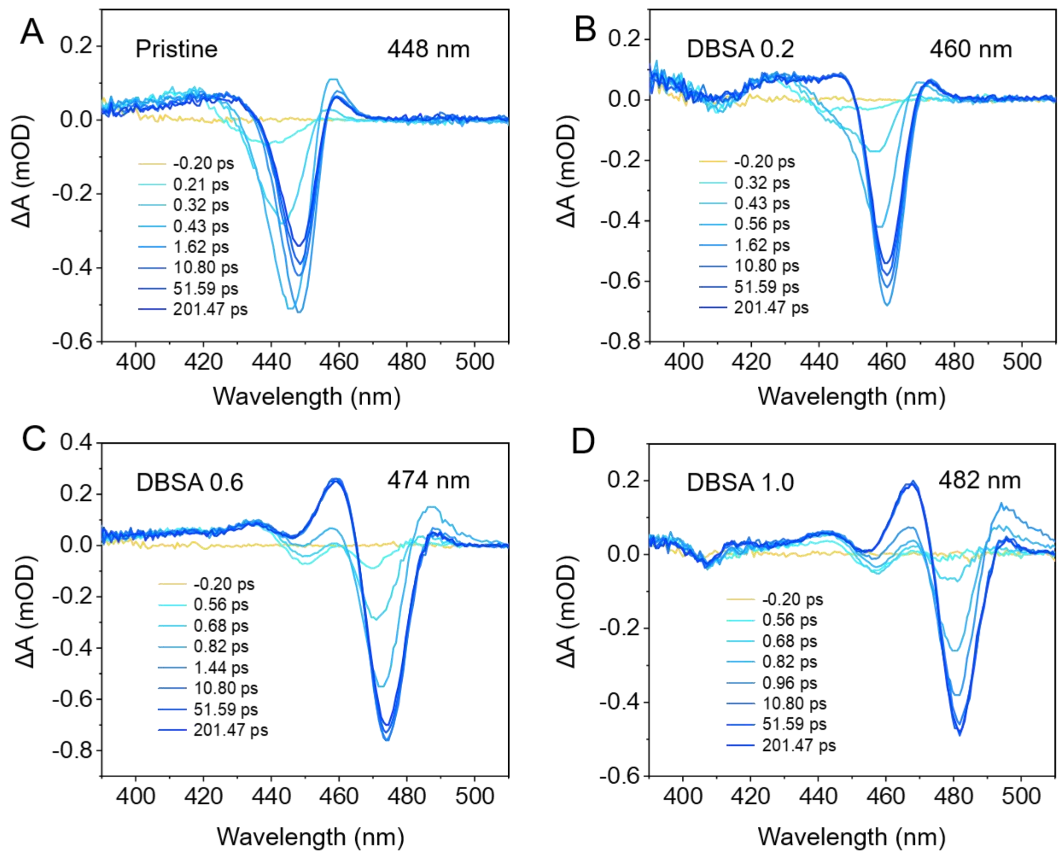

Figure S12. The typical transient absorption spectra of nanocrystals synthesized with different DBSA dosages: pristine (A), DBSA 0.2 mL (B), DBSA 0.6 mL (C), DBSA 1.0 mL (D).

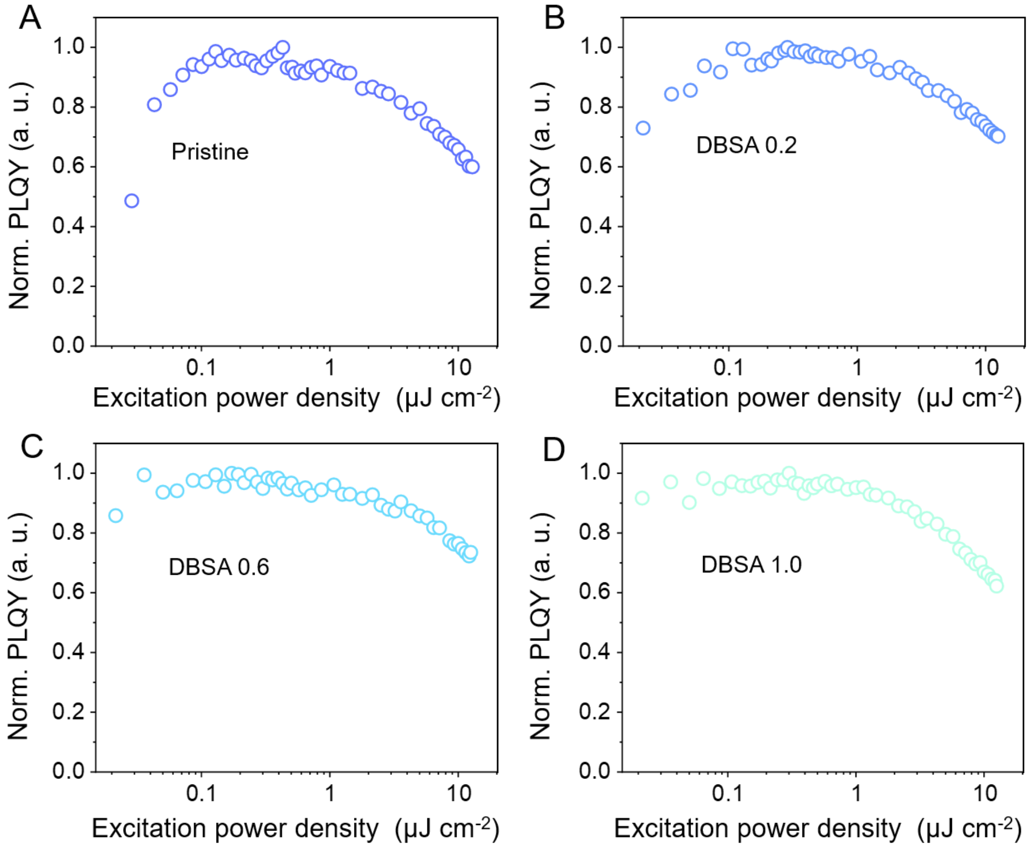

Figure S13. PLQY excitation power density dependence of nanocrystals with varying DBSA dosages: pristine (A), DBSA 0.2 mL (B), DBSA 0.6 mL (C), DBSA 1.0 mL (D). At low excitation power, the PLQY of the pristine sample increases with power density due to defect-state filling. Low carrier concentrations initially lead to defect-assisted recombination dominance (low PLQY), but higher power saturates defects, enhancing excitonic recombination and PLQY, indicating high defect density of pristine nanocrystals. This effect weakens with increased DBSA dosage, confirming defect passivation by DBSA. In intermediate power ranges, stable PLQY independent of excitation power across samples suggests excitonic recombination dominance,[1] aligned with large exciton binding energy of nanocrystals. While at high power, PLQY declines, which reflects Auger recombination dominance.

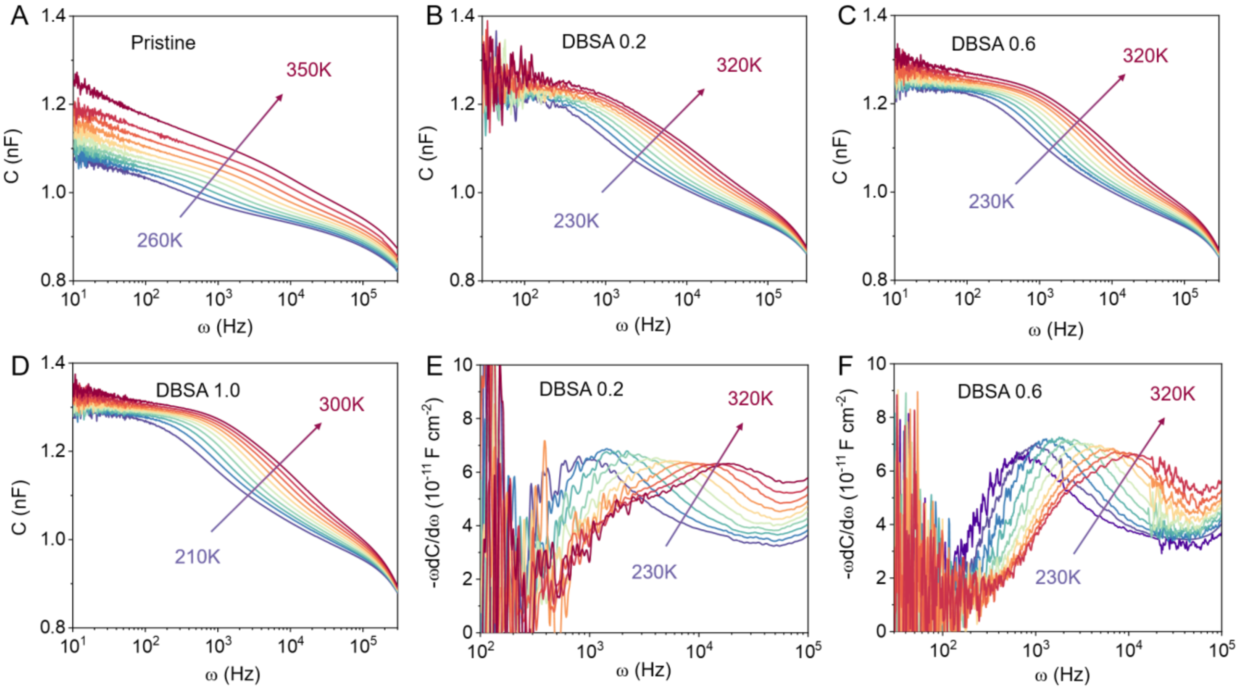

Figure S14. Temperature-dependence of capacitance versus frequency plots for devices based on nanocrystals synthesized with different DBSA dosages: pristine (A), DBSA 0.2 mL (B), DBSA 0.6 mL (C), DBSA 1.0 mL (D). Derivatives of temperature-dependent *C*-*f* plots for devices based on nanocrystals synthesized with DBSA 0.2 mL (E) and 0.6 mL (F).

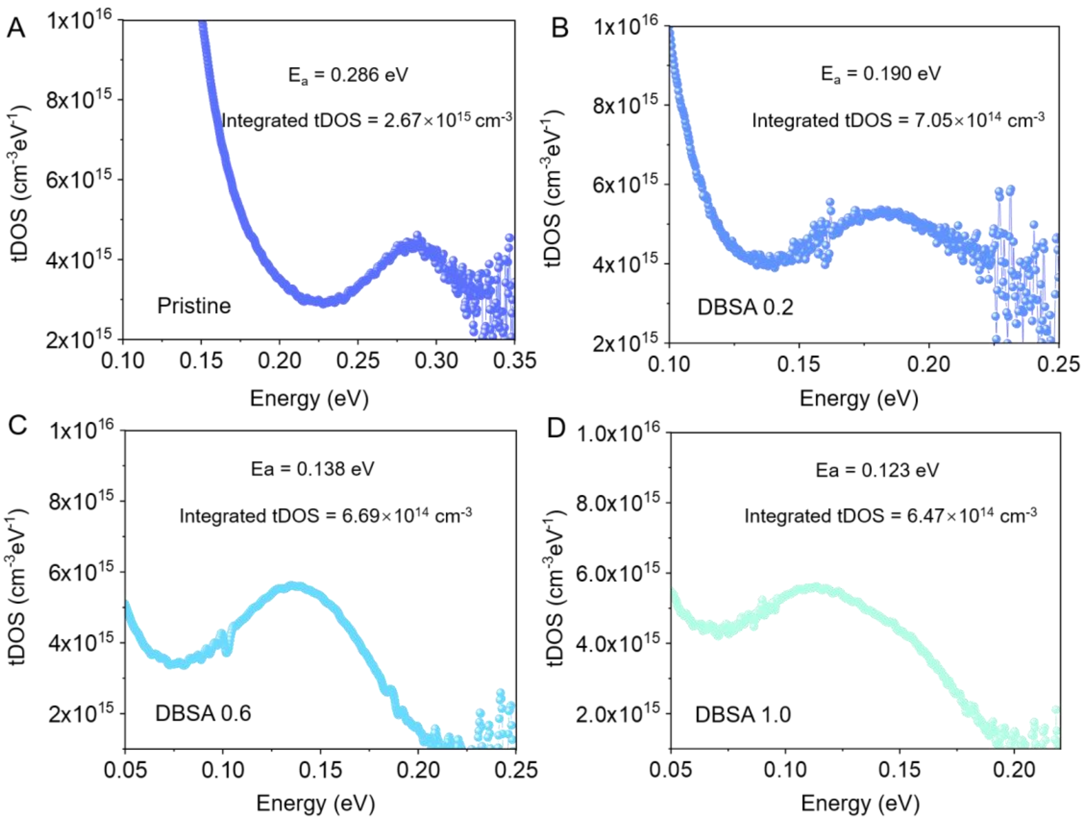

Figure S15. Density of defect states extracted from thermal admittance spectra for nanocrystals synthesized with different DBSA dosages: pristine (A), DBSA 0.2 mL (B), DBSA 0.6 mL (C), DBSA 1.0 mL (D).

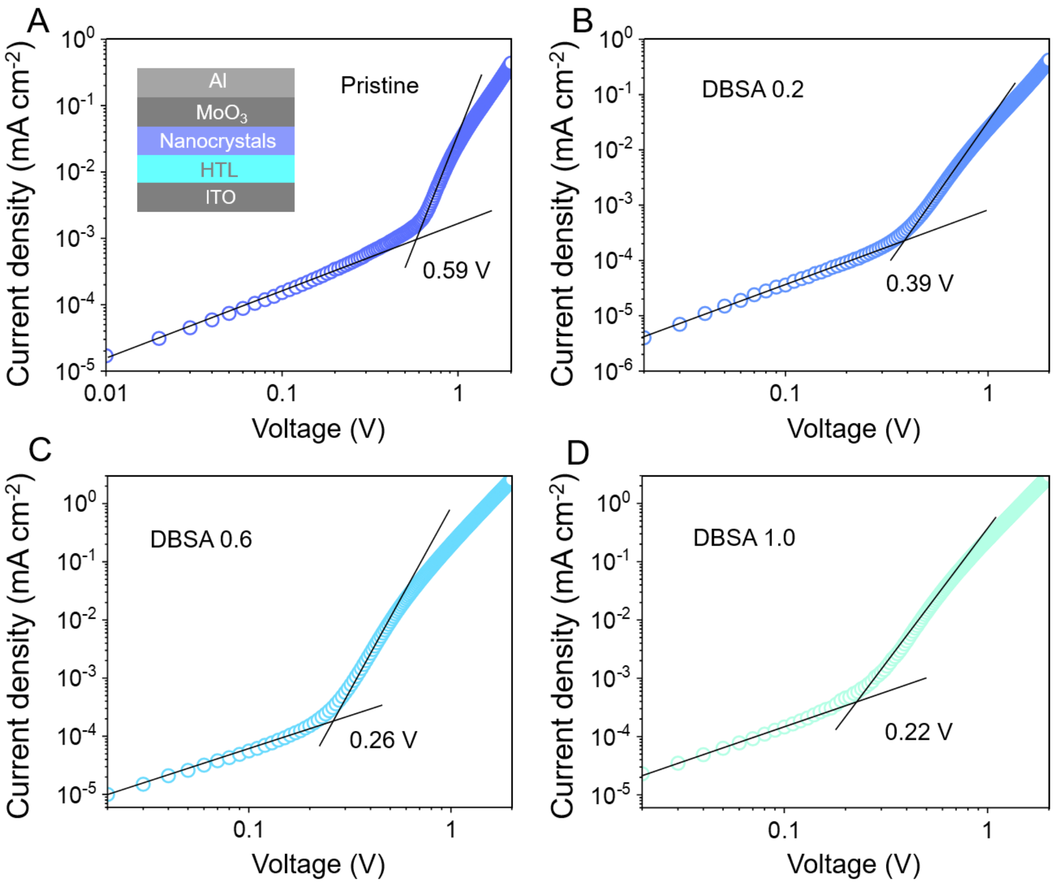

Figure S16. Current density-voltage curves of hole-only devices based on nanocrystals synthesized with different DBSA dosages: pristine (A), DBSA 0.2 mL (B), DBSA 0.6 mL (C), DBSA 1.0 mL (D). The inset shows the corresponding device structures. The extracted trap-filled limit voltages (V_{TFL}) were 0.59 V (Pristine), 0.39 V (DBSA 0.2 mL), 0.26 V (DBSA 0.6 mL), and 0.22 V (DBSA 1.0 mL). The calculated trap state densities derived from these measurements were 2.33×10^{17} cm^{-3}, 1.95×10^{17} cm^{-3}, 1.48×10^{17} cm^{-3}, and 1.25×10^{17} cm^{-3}, respectively.

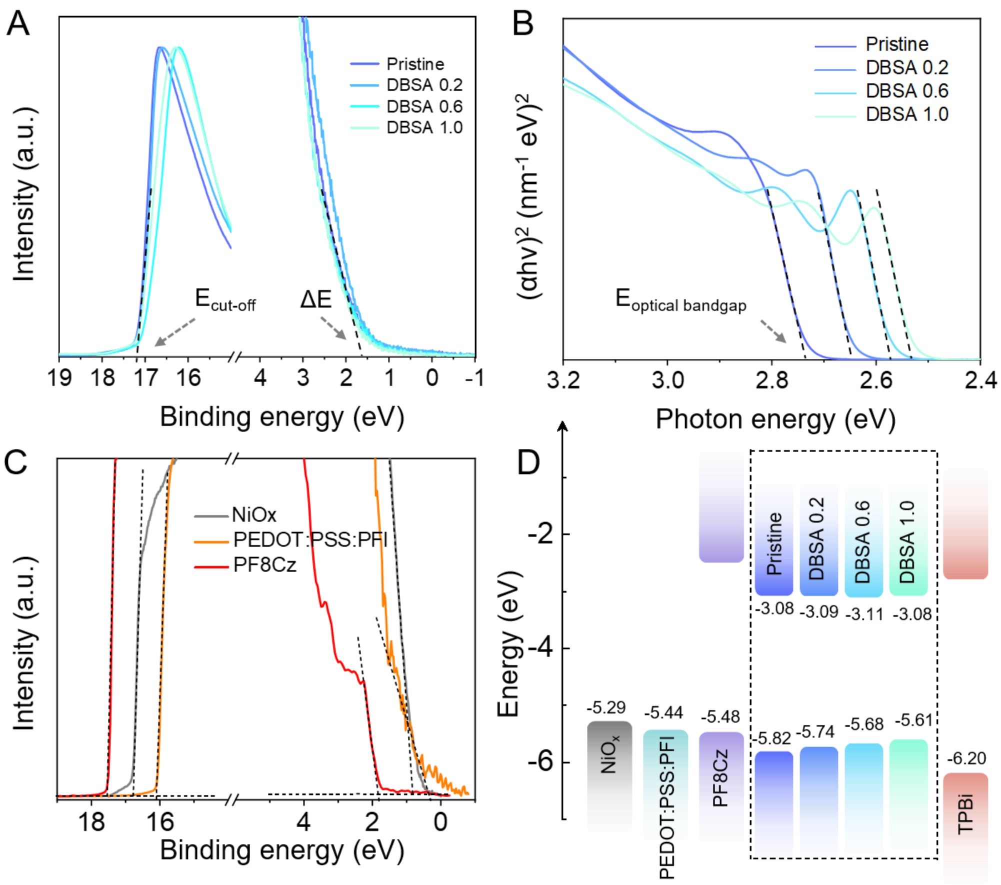

Figure S17. (A) UPS spectra of nanocrystals with different DBSA dosages: Photoemission cut-off region (left) and the valence-band-edge region (right). (B) The plots of $(\alpha h\nu)^2$ versus the photon energy calculated from the absorption measurement. (C) UPS spectra of NiO_x, PEDOT:PSS:PFI, and PF8Cz. (D) Flat-band energy level diagram. The black dashed outline highlights the flat band energy level of nanocrystals with different DBSA dosages. E_{VB} and E_{CB} are calculated with the formula: $E_{VB} = E_{cut\text{-}off} - 21.2 - \Delta E$; $E_{CB} = E_{optical\ bandgap} + E_{VB}$.

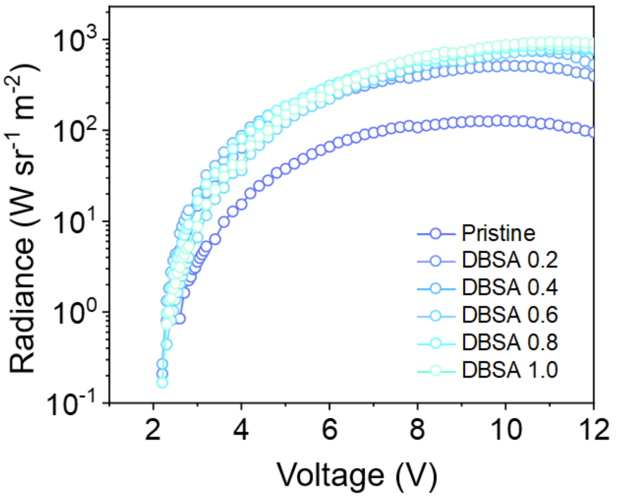

Figure S18. The radiance-voltage curves of the LEDs based on nanocrystals synthesized with different DBSA dosages.

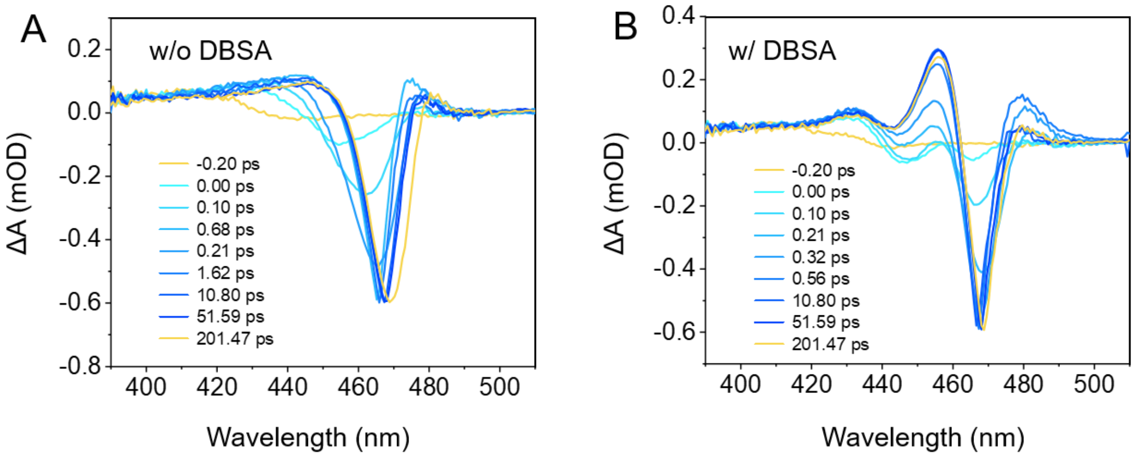

Figure S19. The typical transient absorption spectra of nanocrystals with (A) or without (B) DBSA treatment at the same emission wavelength centred at 470 nm.

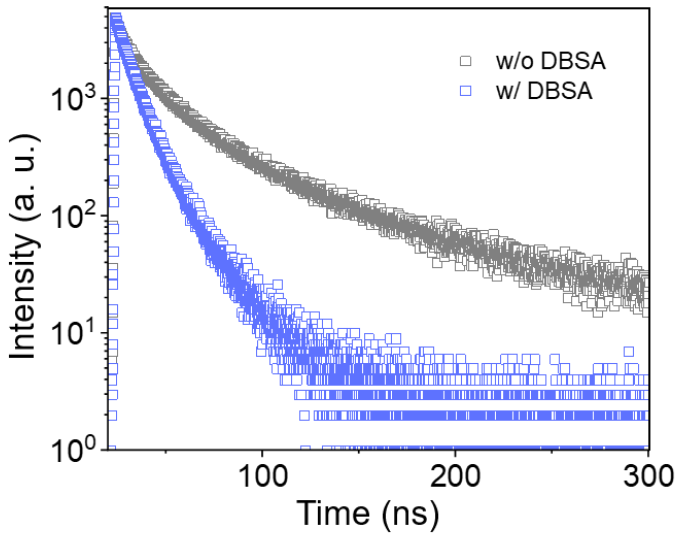

Figure S20. TRPL decay spectra of nanocrystals with or without DBSA treatment at the same emission wavelength centred at 470 nm.

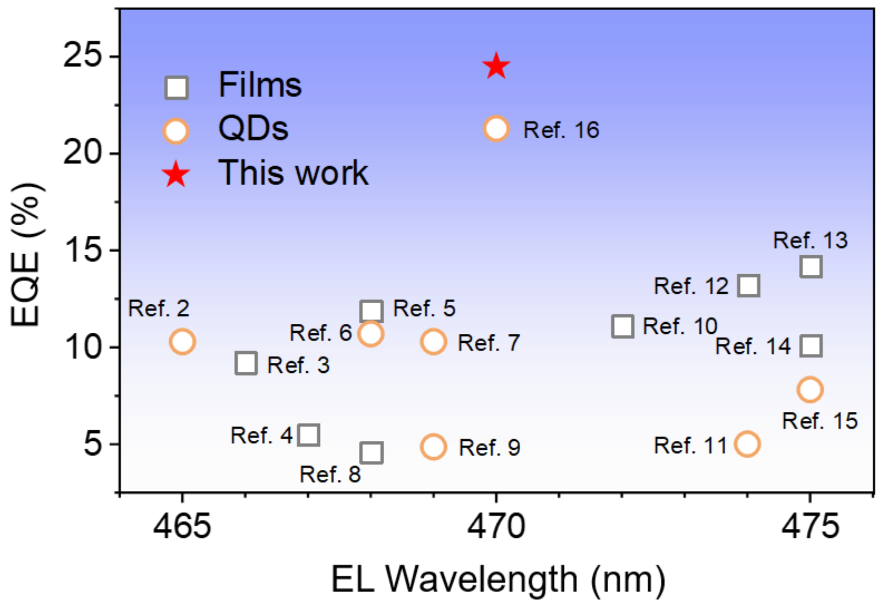

Figure S21. Reported peak EQE and EL wavelength of blue LEDs based on perovskite films or nanocrystals.[2-16]

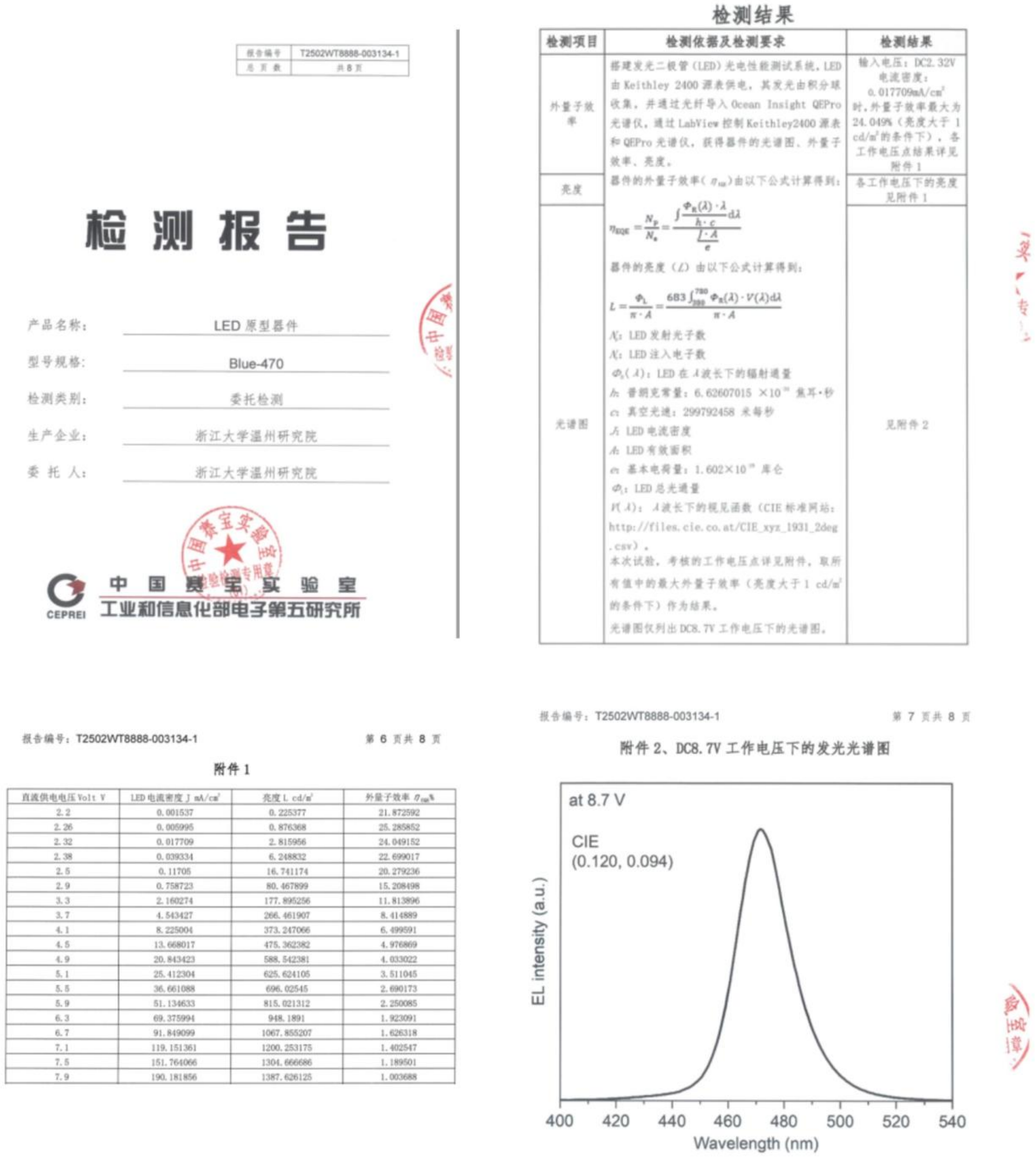

检测结果

检测项目	检测依据及检测要求	检测结果
外量子效率	搭建发光二极管（LED）光电性能测试系统，LED 由 Keithley 2400 源表供电，其发光由积分球收集，并通过光纤导入 Ocean Insight QEPro 光谱仪，通过 LabView 控制 Keithley2400 源表和 QEPro 光谱仪，获得器件的光谱图、外量子效率、亮度。	输入电压：DC2.32V 电流密度：0.017709mA/cm² 时，外量子效率最大为 24.049%（亮度大于 1 cd/m² 的条件下），各工作电压点结果详见附件 1
亮度	器件的外量子效率（η_{EQE}）由以下公式计算得到：	各工作电压下的亮度见附件 1
光谱图	$\eta_{EQE}=\frac{N_p}{N_e}=\frac{\int\frac{\Phi_R(\lambda)\cdot\lambda}{h\cdot c}d\lambda}{\frac{J\cdot A}{e}}$<br>器件的亮度（$L$）由以下公式计算得到：<br>$L=\frac{\Phi_L}{\pi\cdot A}=\frac{683\int_{380}^{780}\Phi_R(\lambda)\cdot V(\lambda)d\lambda}{\pi\cdot A}$<br>$N_p$：LED 发射光子数<br>$N_e$：LED 注入电子数<br>$\Phi_R(\lambda)$：LED 在 λ 波长下的辐射通量<br>h：普朗克常量：6.62607015 ×10[illegible] 焦耳·秒<br>c：真空光速：299792458 米每秒<br>J：LED 电流密度<br>A：LED 有效面积<br>e：基本电荷量：1.602×10[illegible] 库仑<br>Φ_L：LED 总光通量<br>$V(\lambda)$：λ 波长下的视见函数（CIE 标准网站：http://files.cie.co.at/CIE_xyz_1931_2deg.csv）。<br>本次试验，考核的工作电压点详见附件，取所有值中的最大外量子效率（亮度大于 1 cd/m² 的条件下）作为结果。<br>光谱图仅列出 DC8.7V 工作电压下的光谱图。	见附件 2

报告编号：T2502WT8888-003134-1 第 6 页共 8 页

附件 1

直流供电电压 Volt V	LED 电流密度 J mA/cm²	亮度 L cd/m²	外量子效率 η_{EQE}%
2.2	0.001537	0.225377	21.872592
2.26	0.005995	0.876368	25.285852
2.32	0.017709	2.815956	24.049152
2.38	0.039334	6.248832	22.699017
2.5	0.11705	16.741174	20.279236
2.9	0.758723	80.467899	15.208498
3.3	2.160274	177.895256	11.813896
3.7	4.543427	266.461907	8.414889
4.1	8.225004	373.247066	6.499591
4.5	13.668017	475.362382	4.976869
4.9	20.843423	588.542381	4.033022
5.1	25.412304	625.624105	3.511045
5.5	36.661088	696.02545	2.690173
5.9	51.134633	815.021312	2.250085
6.3	69.375994	948.1891	1.923091
6.7	91.849099	1067.855207	1.626318
7.1	119.151361	1200.253175	1.402547
7.5	151.764066	1304.666686	1.189501
7.9	190.181856	1387.626125	1.003688

报告编号：T2502WT8888-003134-1 第 7 页共 8 页

附件 2、DC8.7V 工作电压下的发光光谱图

Figure S22. Certification by CEPREI (China Electronic Product Reliability and Environmental Testing Research Institute) with a certified maximum EQE of 24.0%. To clarify the validity of EQE data at low voltages (preventing accidental jumps or outliers), a segmented voltage application method was adopted for testing. Smaller voltage steps (0.06 V) were used at lower voltages, while larger voltage steps (0.4 V) were applied at higher voltages.

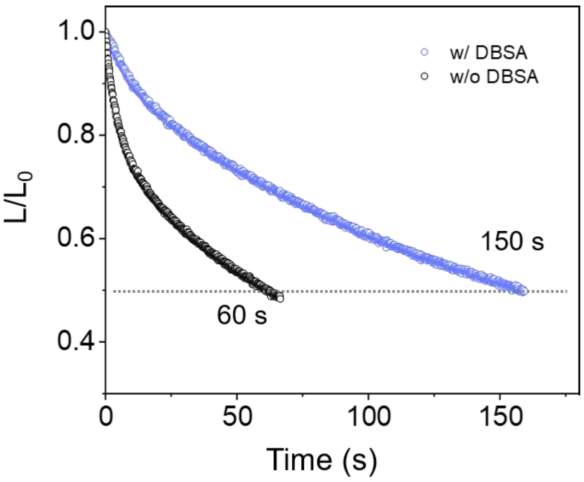

Figure S23. Normalized luminance as a function of working time for LEDs at an initial luminance of 100 cd m^{-2} with and without the DBSA group.

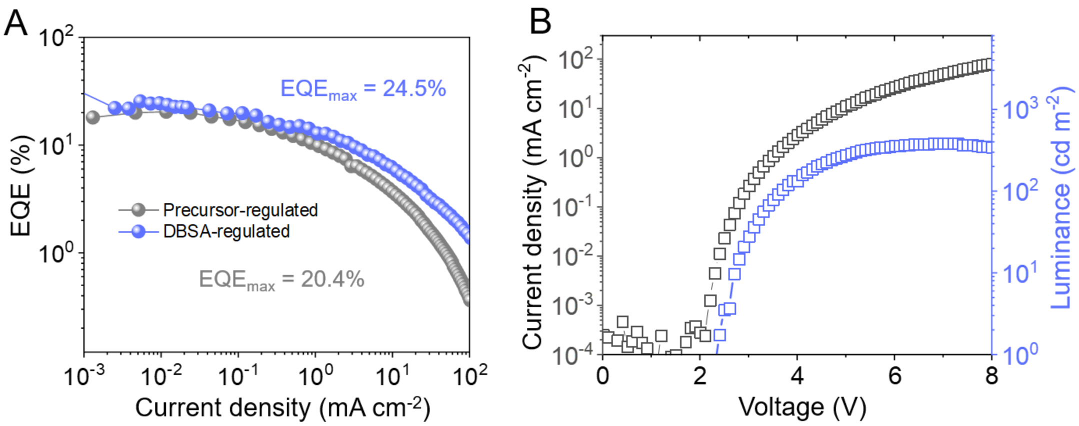

Figure S24 (A) EQE-current density curves of the LEDs based on nanocrystals with Cl/Br ratio regulated by precursor or DBSA. (B) Typical current density–voltage-luminance curves of the LED based on nanocrystals with a Cl/Br ratio regulated by precursor.

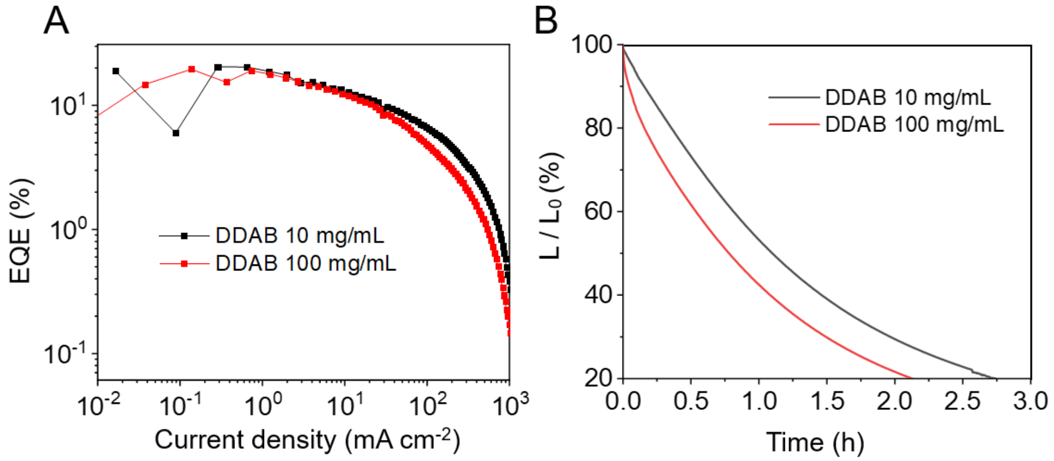

Figure S25 (A) EQE-current density curves of the LEDs based on pure-Br nanocrystals passivated by different dosages of DDAB and (B) normalized luminance as a function of working time for LEDs at an initial luminance of 100 cd m^{-2}.

Table S1. The precise halogen content in nanocrystals synthesized with different DBSA dosages measured by Ion chromatography.

**Nanocrystals**	**Cl^- (mg mL^{-1})**	**Cl^- (mmol mL^{-1})**	**Br^- (mg mL^{-1})**	**Br^- (mmol mL^{-1})**	**Cl: Br (molar ratio)**
Pristine	0.5028	0.01418	0.9773	0.01223	1.159
DBSA 0.2	0.4110	0.01159	1.3158	0.01647	0.7037
DBSA 0.4	0.2964	0.00836	1.4079	0.01762	0.4745
DBSA 0.6	0.2579	0.00728	1.4933	0.01869	0.3895
DBSA 0.8	0.2166	0.00611	1.5374	0.01924	0.3176
DBSA 1.0	0.2072	0.00584	1.7481	0.02188	0.2669

Table S2. Synthesis conditions of nanocrystals with or without DBSA at an emission wavelength of 470 nm (dissolved in 5 mL of toluene, with 0.5 mL of Cs-PA triggering the reaction).

**Nanocrystals**	**$PbBr_2$**	**$PbCl_2$**	**TOAB**	**DBSA ($1\ g\ mL^{-1}$)**	**DDAC ($0.1\ g\ mL^{-1}$)**	**DDAB ($0.1\ g\ mL^{-1}$)**
w/ DBSA	0.4 mmol	0.1 mmol	0.725 mL	0.6 mL	1.2 mL	0 mL
w/o DBSA	0.4 mmol	0.1 mmol	0.725 mL	0 mL	0.2 mL	1.0 mL

REFERENCES

1. Jiang Y., Cui M., Li S., et al. (2021). Reducing the impact of Auger recombination in quasi-2D perovskite light-emitting diodes. *Nat. Commun.* **12**:336. DOI:10.1038/s41467-020-20555-9

2. Jiang Y., Sun C., Xu J., et al. (2022). Synthesis-on-substrate of quantum dot solids. *Nature* **612**:679-684. DOI:10.1038/s41586-022-05486-3

3. Hu J., Li J., Lu G., et al. (2024). Monoammonium Modified Dion-Jacobson Quasi-2D Perovskite for High Efficiency Pure-Blue Light Emitting Diodes. *Small* **20**:2402786. DOI:10.1002/smll.202402786

4. Karlsson M., Yi Z., Reichert S., et al. (2021). Mixed halide perovskites for spectrally stable and high-efficiency blue light-emitting diodes. *Nat. Commun.* **12**:361. DOI:10.1038/s41467-020-20582-6

5. Liu Y., Wang S., Yu Z., et al. (2023). A Multifunctional Additive Strategy Enables Efficient Pure-Blue Perovskite Light-Emitting Diodes. *Adv. Mater.* **35**:2302161. DOI:10.1002/adma.202302161

6. Bi C., Yao Z., Hu J., et al. (2023). Suppressing Auger Recombination of Perovskite Quantum Dots for Efficient Pure-Blue-Light-Emitting Diodes. *ACS Energy Lett.* **8**:731-739. DOI:10.1021/acsenergylett.2c02613

7. Wei S., Hu J., Bi C., et al. (2024). Strongly-Confined CsPbBr3 Perovskite Quantum Dots with Ultralow Trap Density and Narrow Size Distribution for Efficient Pure-Blue Light-Emitting Diodes. *Small* **20**:2400885. DOI:10.1002/smll.202400885

8. Tong Y., Bi X., Xu S., et al. (2023). In Situ Halide Exchange of Cesium Lead Halide Perovskites for Blue Light-Emitting Diodes. *Adv. Mater.* **35**:2207111. DOI:10.1002/adma.202207111

9. Chen F., Liu Y., Zhang D., et al. (2023). Bilayer phosphine oxide modification toward efficient and large-area pure-blue perovskite quantum dot light-emitting diodes. *Sci. Bull.* **68**:2354-2361. DOI:10.1016/j.scib.2023.09.014

10. Jiang M., Zhang X. and Wang F. (2024). Efficient Perovskite Nanograin Light-Emitting Diodes in Green-to-Blue Gamut with Co-Additive Engineering. *Adv. Mater.* **36**:2400565. DOI:10.1002/adma.202400565

11. Gao L., Zhang Y., Gou L., et al. (2022). High efficiency pure blue perovskite quantum dot light-emitting diodes based on formamidinium manipulating carrier dynamics and electron state filling. *Light-Sci. Appl.* **11**:346. DOI:10.1038/s41377-022-00992-5

12. Yuan S., Dai L., Sun Y., et al. (2024). Efficient blue electroluminescence from reduced-dimensional perovskites. *Nat. Photon.* **18**:425-431. DOI:10.1038/s41566-024-01382-6

13. Zhang L., Jiang Y., Feng Y., et al. (2023). Manipulating Local Lattice Distortion for Spectrally Stable and Efficient Mixed-halide Blue Perovskite LEDs. *Angew. Chem. Int. Ed.* **62**:e202302184. DOI:10.1002/anie.202302184

14. Yang Y., Xu S., Ni Z., et al. (2021). Highly Efficient Pure-Blue Light-Emitting Diodes Based on Rubidium and Chlorine Alloyed Metal Halide Perovskite. *Adv. Mater.* **33**:2100783. DOI:10.1002/adma.202100783

15. Ma L., Li X., Li X., et al. (2024). Eliminating Chlorine Vacancies of Perovskite Nanocrystals Using Hydrazine Cations Enables Efficient Pure Blue Light-Emitting Diodes. *ACS Energy Lett.* **9**:1210-1218. DOI:10.1021/acsenergylett.4c00109

16. Gao Y., Cai Q., He Y., et al. (2024). Highly efficient blue light-emitting diodes based on mixed-halide perovskites with reduced chlorine defects. *Sci. Adv.* **10**:eado5645. DOI:10.1126/sciadv.ado5645
